# Identification of community structure-based brain states and transitions using functional MRI

**DOI:** 10.1016/j.neuroimage.2021.118635

**Published:** 2021-12-01

**Authors:** Lingbin Bian, Tiangang Cui, B.T. Thomas Yeo, Alex Fornito, Adeel Razi, Jonathan Keith

**Affiliations:** a School of Mathematics, Monash University, Australia; b Turner Institute for Brain and Mental Health, School of Psychological Sciences, Monash University, Australia; c Department of Electrical and Computer Engineering, National University of Singapore, Singapore; d Monash Biomedical Imaging, Monash University, Australia; e Wellcome Centre for Human Neuroimaging, University College London, United Kingdom; f CIFAR Azrieli Global Scholars Program, CIFAR, Toronto, Canada

**Keywords:** Dynamic functional connectivity, Change-point detection, Latent block model, Bayesian inference, Markov chain Monte Carlo

## Abstract

Brain function relies on a precisely coordinated and dynamic balance between the functional integration and segregation of distinct networks. Characterizing the way in which brain regions reconfigure their interactions to give rise to distinct but hidden brain states remains an open challenge. In this paper, we propose a Bayesian method for characterizing community structure-based latent brain states and showcase a novel strategy based on posterior predictive discrepancy using the latent block model to detect transitions between community structures in blood oxygen level-dependent (BOLD) time series. The set of estimated parameters in the model includes a latent label vector that assigns network nodes to communities, and also block model parameters that reflect the weighted connectivity within and between communities. Besides extensive in-silico model evaluation, we also provide empirical validation (and replication) using the Human Connectome Project (HCP) dataset of 100 healthy adults. Our results obtained through an analysis of task-fMRI data during working memory performance show appropriate lags between external task demands and change-points between brain states, with distinctive community patterns distinguishing fixation, low-demand and high-demand task conditions.

## Highlights

• Community-based detection of discrete brain states using stochastic latent block model.
• Bayesian change-point detection and model selection via posterior predictive discrepancy.
• Markov chain Monte Carlo methods for estimation of community memberships.
• Distinctive brain states for varying task demands in working memory task fMRI.

## Graphical abstract

**Figure:**
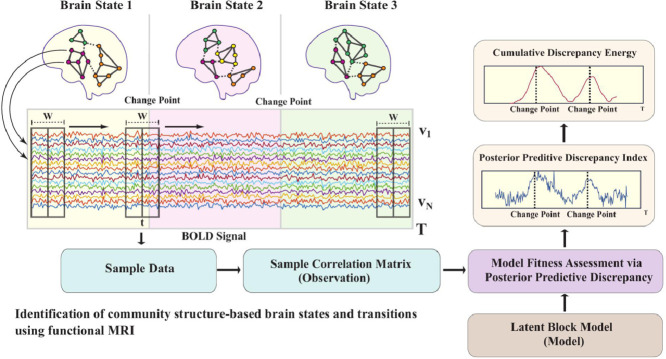

## 1 Introduction

Identifying changes in functional brain networks over time, under various cognitive states, can provide insights into dynamical organisation of the human brain. However, the definition of discrete brain states and the methods for identifying these states have not been commonly agreed (Kringelbach, Deco, 2020, Lurie, Kessler, Bassett, Betzel, Breakspear, Kheilholz, Kucyi, Liégeois, Lindquist, McIntosh, Poldrack, Shine, Thompson, Bielczyk, Douw, Kraft, Miller, Muthuraman, Pasquini, Razi, Vidaurre, Xie, Calhoun, 2020). Experiments targeting unconstrained spontaneous ‘resting-state’ fMRI (Allen, Damaraju, Plis, Erhardt, Eichele, Calhoun, 2014, Aquino, Fulcher, Parkes, Sabaroedin, Fornito, 2020, Calhoun, Miller, Pearlson, Adali, 2014, Friston, Fagerholm, Zarghami, Parr, Hipólito, Magrou, Razi, 2021, Friston, Kahan, Biswal, Razi, 2014, Hutchison, Womelsdorf, Allen, Bandettini, Calhoun, Corbetta, Penna, Duyn, Glover, Gonzalez-castillo, Handwerker, Keilholz, Kiviniemi, Leopold, Pasquale, Sporns, Walter, Chang, 2013, Lurie, Kessler, Bassett, Betzel, Breakspear, Kheilholz, Kucyi, Liégeois, Lindquist, McIntosh, Poldrack, Shine, Thompson, Bielczyk, Douw, Kraft, Miller, Muthuraman, Pasquini, Razi, Vidaurre, Xie, Calhoun, 2020, Parkes, Fulcher, Yücel, Fornito, 2018, Power, Plitt, Laumann, Martin, 2017, Razi, Friston, 2016, Razi, Kahan, Rees, Friston, 2015, Razi, Seghier, Zhou, McColgan, Zeidman, Park, Sporns, Rees, Friston, 2017) have limited ability to infer latent brain states or determine how the brain segues from one state to another, because the cognitive or vigilance states are unpredictable and there is no ground truth regarding the transient changes of cognition during resting state. A recent study with naturalistic movie stimuli used a hidden Markov model to explore dynamic jumps between discrete brain states and found that the variations in the sensory and narrative properties of the movie can evoke discrete brain processes (Meer et al., 2020). However, the dynamics of brain states and functional networks are not induced only by external stimuli, but also by unknown intrinsic latent mental processes (Lurie, Kessler, Bassett, Betzel, Breakspear, Kheilholz, Kucyi, Liégeois, Lindquist, McIntosh, Poldrack, Shine, Thompson, Bielczyk, Douw, Kraft, Miller, Muthuraman, Pasquini, Razi, Vidaurre, Xie, Calhoun, 2020, Taghia, Cai, Ryali, Kochalka, Nicholas, Chen, Menon, 2018). Task-fMRI studies with external stimuli have demonstrated that functional connectivity exhibits variation during motor learning (Bassett et al., 2011) and anxiety-inducing speech preparation (Cribben et al., 2012). Task-fMRI experiments can, to a large extent, delineate the external stimuli (e.g., the onset and duration of stimuli in experiments with block design), which can be used to validate methods for identifying latent discrete brain states. Although task-based fMRI constitutes reference points against which to identify changes in the observed signal, this information does not precisely determine the timing and duration of the latent brain state relative to psychological processes. For example, an emotional stimulus may trigger a latent cognitive response which is delayed relative to stimulus onset and which persists for some time after stimulus offset. Therefore, the development of noninvasive methods for identifying transitions of latent brain states during both task performance and task-free conditions is necessary for characterizing the spatiotemporal dynamics of brain networks.

Change-point detection in multivariate time series is a statistical problem that has clear relevance to identifying transitions in brain states, particularly in the absence of knowledge regarding the experimental design. Several change-point detection methods based on spectral clustering (Cribben, Yu, 2017, Luxburg, 2007) and dynamic connectivity regression (DCR) (Cribben et al., 2012) have been previously developed and applied to the study of fMRI time series, and these have enhanced our understanding of brain dynamics. However, change-point detection with spectral clustering only evaluates changes to the component eigenstructures of the networks but neglects the weighted connectivity between nodes, while the DCR method only focuses on the sparse graph but ignores the modules of the brain networks. Other change-point detection strategies include a frequency-specific method (Schröder and Ombao, 2019), applying a multivariate cumulative sum procedure to detect change-points using EEG data, and methods which focus on large scale network estimation in fMRI time series (Cho, Fryzlewicz, 2015, Frick, Munk, Sieling, 2014, Park, Friston, Pae, Park, Razi, 2018, Wang, Samworth, 2017). Many fMRI studies use sliding window methods for characterizing the time-varying functional connectivity in time series analysis (Allen, Damaraju, Plis, Erhardt, Eichele, Calhoun, 2014, Chang, Glover, 2010, Handwerker, Roopchansingh, Gonzalez-Castillo, Bandettini, 2012, Jeong, Pae, Park, 2016, Lurie, Kessler, Bassett, Betzel, Breakspear, Kheilholz, Kucyi, Liégeois, Lindquist, McIntosh, Poldrack, Shine, Thompson, Bielczyk, Douw, Kraft, Miller, Muthuraman, Pasquini, Razi, Vidaurre, Xie, Calhoun, 2020, Monti, Hellyer, Sharp, Leech, Anagnostopoulos, Montana, 2014, Zalesky, Fornito, Cocchi, Gollo, Breakspear, 2014). Methods based on hidden Markov models are also widely used to analyze transient brain states (Vidaurre, Abeysuriya, Becker, Quinn, Alfaro-Almagro, Smith, Woolrich, 2018, Vidaurre, Quinn, Baker, Dupret, Tejero-Cantero, Woolrich, 2016, Vidaurre, Smith, Woolrich, 2017).

A *community* is defined as a collection of nodes that are densely connected in a network. The problem of community detection is a topical area of network science (Jin, 2015, Sporns, Betzel, 2016, Wang, Bickel, 2017). How communities change or how the nodes in a network are assigned to specific communities is an important problem in the characterization of networks. Although many community detection problems in network neuroscience are based on modularity (Bassett, Porter, Wymbs, Grafton, Carlson, Mucha, 2013, Bassett, Wymbs, Porter, Mucha, Carlson, Grafton, 2011, Newman, 2006), recently a hidden Markov stochastic block model combined with a non-overlapping sliding window was applied to infer dynamic functional connectivity for networks, where edge weights were only binary and the candidate time points evaluated were not consecutive (Robinson et al., 2015). More general weighted stochastic block models (Aicher et al., 2015) have been used to infer structural connectivity for human lifespan analysis (Faskowitz et al., 2018) and to infer functional connectivity in the mesoscale architecture of drosophila, mouse, rat, macaque, and human connectomes (Betzel et al., 2018). However, these studies using the weighted stochastic block model only explore the brain network over the whole time course of the experiment and neglect dynamic properties of networks. Weighted stochastic block models (Aicher et al., 2015) are described in terms of exponential families (parameterized probability distributions), with the estimation of parameters performed using variational inference (Blei, Kucukelbir, McAuliffe, 2017, Hoffman, Blei, Wang, Paisley, 2013). Another relevant statistical approach introduces a fully Bayesian latent block model (Nobile, Fearnside, 2007, Wyse, Friel, 2012), which includes both a binary latent block model and a Gaussian latent block model as special cases. The Gaussian latent block model is similar to the weighted stochastic block model, but different methods have been used for parameter estimation, including Markov chain Monte Carlo (MCMC) sampling.

Although there is a broad literature exploring change-point detection, and also many papers that discuss community detection, relatively few papers combine these approaches, particularly from a Bayesian perspective. In this paper, we develop Bayesian methods which unify change-point detection and community detection to explore when and how the community structure of discrete brain state changes at different time points. The methods are validated using extensive synthetic data and working memory task fMRI data under different external demands. There are several advantages of our approach compared to existing change-point detection methods. Compared to the methods like spectral clustering (Cribben, Yu, 2017, Luxburg, 2007) and DCR (Cribben et al., 2012), which either ignore characterizing the weighted connectivity or the community patterns, the fully Bayesian framework and Markov chain Monte Carlo method provide flexible and powerful strategies that have been under-used for characterizing the latent properties of brain networks, including the dynamics of both the community memberships and weighted connectivity properties of the nodal community structures. The change-point detection method based on stochastic block model uses non-overlapping sliding windows and is applied only to binary brain networks (Robinson et al., 2015). In contrast to the binary latent block model used in time-varying network study (Bian et al., 2020), the Gaussian latent block model used in this paper considers the correlation matrix as an observation without imposing any arbitrary thresholds, so that all the information contained in the time series is preserved, resulting in more accurate detection of change-points. Moreover, unlike methods based on fixed community memberships over the time course (Ting et al., 2021), our methods consider both the community memberships and parameters related to the weighted connectivity to be time varying, which results in more flexible estimation of both community structure and connectivity patterns. Furthermore, our Bayesian change-point detection (BCPD) method uses overlapping sliding windows that assess *all* of the potential candidate change-points over the time course, which increases the resolution of the detected change-points compared to method using non-overlapping windows (Robinson et al., 2015). Finally, the proposed BCPD is computationally efficient, scaling to whole-brain networks potentially covering hundreds of nodes within a reasonable time frame in the order of tens of minutes.

Our paper presents four main contributions, namely: (i) we quantitatively characterize discrete brain states of community structure with weighted connectivity and time-dependent community memberships, using the latent block model within a temporal interval between two consecutive change-points; (ii) we propose a new Bayesian change-point detection method based on *posterior predictive discrepancy* (PPD) (Bian, Cui, Sofronov, Keith, 2020, Gelman, Meng, Stern, 1996) to estimate transition locations between brain states, using a Bayesian model fitness assessment; (iii) in addition to the locations of change-points, we also infer the community architectures of discrete brain states, which we show are distinctive for 2-back, 0-back, and fixation conditions in a working-memory task-based fMRI experiment, and; (iv) we further empirically find that the estimated change-points between brain states show appropriate lags compared to the external working memory task conditions.

## 2 Material and methods

### 2.1 The framework of Bayesian change-point detection

**Fig. 1:**
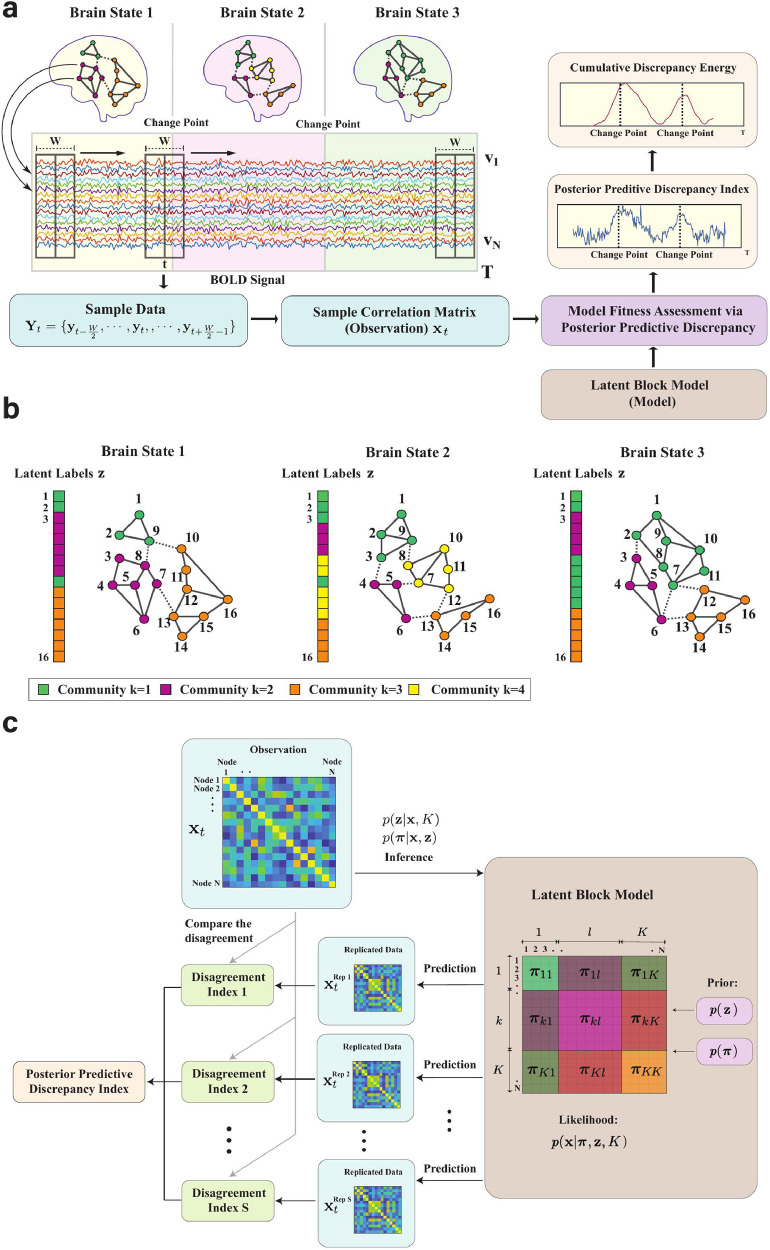
The framework for identifying brain states, transitions and communities. **a** Schematic of the proposed Bayesian change-point detection (BCPD) method. Three different background colors represent three brain states of individual subjects with different community architectures. The colors of the nodes represent community memberships. A sliding window of width W centered at t is applied to the time series. The different colored time series correspond to BOLD time series for each node. The sample correlation matrix $x_{t}$ (i.e., an observation for our Bayesian model) is calculated from the sample data $Y_{t}$ within the sliding window. We use the Gaussian latent block model to fit the observations and evaluate goodness of fit between model and the observations to obtain the posterior predictive discrepancy index (PPDI). We then calculate the cumulative discrepancy energy (CDE) from the PPDI and use the CDE as a scoring criterion to estimate the change-points of the community architectures. **b** Dynamic community memberships of networks with N = 16 nodes. A latent label vector z contains the labels (k) of specific communities for the nodes. Nodes of the same color are located in the same community. The dashed lines represent the (weighted) connectivity between communities and the solid lines represent the (weighted) connectivity within the communities. **c** Model fitness assessment. The observation is the realized adjacency matrix; different colors in the latent block model represent different blocks with the diagonal blocks representing the connectivity within a community and the off-diagonal blocks representing the connectivity between communities. To demonstrate distinct blocks of the latent block model, in this schematic we group the nodes in the same community adjacently and the communities are sorted. In reality, the labels of the nodes are mixed with respect to an adjacency matrix. The term $\pi_{kl}$ represents the model parameters in block kl.

An overview of the BCPD framework is shown in Fig. 1a. We consider a collection of N nodes $\left\{v_{1},\ldots ,v_{N}\right\}$ representing brain regions for a single subject, and suppose that we observe a collection of N time series $Y\in {ℜ}^{N\times T}$ where $Y=(y_{1},y_{2},\ldots ,y_{T})$, and T is the number of time points. Different background colors represent different latent network community architectures. The nodes in the networks are assumed to be clustered into communities and the different colors of the nodes represent the different community memberships. A more detailed example of changes in network architectures with 16 nodes is shown in Fig. 1b, where the community memberships are defined as a latent label vector z and K is the number of communities. A transition or change-point is defined as a time point at which the community structure changes. Correlations between time series suggest interactions between the corresponding brain regions; we therefore first process the time series to construct a sequence of graphs in which temporal correlations between time series are represented by an edge connecting the corresponding nodes.

We apply a sliding window of width W (even numbered) to the time series as shown in Fig. 1a. The sliding windows overlap and the centers of the windows are located at consecutive time points. Change-points may occur only at times $t\in \{\frac{W}{2}+1,\ldots ,T-\frac{W}{2}\}$ where $\frac{W}{2}$ is a margin size used to avoid computational and statistical complications. The advantage of using overlapping windows is that we can potentially detect transitions in network architecture at any time during the time course (except the margin area). For each time point $t\in \{\frac{W}{2}+1,\ldots ,T-\frac{W}{2}\}$, we define $Y_{t}=\left\{y_{t-\frac{W}{2}},\ldots ,y_{t},\ldots ,y_{t+\frac{W}{2}-1}\right\}$ as the data in the sliding window at time t and calculate a sample correlation matrix $x_{t}$ within this window. We interpret this correlation matrix as a weighted adjacency matrix. This means for each t, we obtain a sample adjacency matrix $x_{t}$. Subsequently, instead of time series Y, we use the sample adjacency matrix $x_{t}$ as the realized observation at time t.

Fig. 1 c provides a schematic illustrating the posterior predictive model fitness assessment. Specifically, we propose to use the Gaussian latent block model (Wyse and Friel, 2012) to quantify the likelihood of a network, and the MCMC allocation sampler (Nobile, Fearnside, 2007, Wyse, Friel, 2012) to infer a latent label vector z from a collapsed posterior distribution p(z|x,K) derived from this model. The model parameters π for each block are sampled from a posterior distribution p(π|x,z), conditional on the sampled latent label vector z. The proposed model fitness procedure draws parameters (both latent label vectors and model parameters) from posterior distributions and uses them to generate a replicated adjacency matrix $x^{rep}$. It then calculates a disagreement index to quantify the difference between the replicated adjacency matrix $x^{rep}$ and realized adjacency matrix x. To evaluate model fitness, we use the parameter-dependent statistic called the posterior predictive discrepancy index (PPDI) by averaging the disagreement index. More thorough discussion of PPDI is provided later in Section 2.5.

### 2.2 The latent block model

The latent block model (LBM) (Wyse and Friel, 2012) is a random process generating networks on a fixed number of nodes N. The model has an integer parameter K, representing the number of communities. Identifying a suitable value of K is a model fitting problem that will be discussed in Section 2.4; here we assume K is given. A schematic of a latent block model is shown in the brown box on the right side of Fig. 1c. A defining feature of the model is that nodes are partitioned into K communities, with interactions between nodes in the same community having a different (usually higher) probability than interactions between nodes in different communities. The latent block model first assigns the N nodes into the K communities resulting in $K^{2}$ blocks, which are symmetric, then generates edges with a probability determined by the community memberships. The diagonal blocks represent the connectivity within the communities and the off-diagonal blocks represent the connectivity between different communities. In our previous work, we developed the change-point detection algorithm based on stochastic block model where the edges are binary (Bian et al., 2020) and the block model parameter matrix only contains the blocks of mean. In this paper, we consider the edges between nodes to be weighted, so the model parameter matrix π consists of the means and variances that determine the connectivity in the blocks. We treat the correlation matrix as an observation, thus preserving more information from the BOLD time series than using binary edges. Given a sampled z we can draw π from the posterior directly. For mathematical illustration of the latent block model, see **SI Section 1.1 and 1.2**. Methods for sampling the latent label vector z will be discussed next.

### 2.3 Sampling from the posterior

The posterior predictive method we outline below involves sampling parameters from the posterior distribution. The sampled parameters are the latent label vector z and model parameter matrix π. There are several methods for estimating the latent labels and model parameters of a latent block model described in the literature. One method evaluated the model parameters by point estimation but considered the latent labels in z as having a distribution (Daudin et al., 2008), making this approach similar to an EM algorithm. Another method used point estimation for both the model parameters and latent labels (Zanghi et al., 2008). We sample the latent label vector z from the collapsed posterior p(z|x,K) (see detailed derivation of p(z|x,K) in **SI Section 1.3**). We use the Markov chain Monte Carlo (MCMC) (Hastings, 1970) method to sample the latent label vector from the posterior using Gibbs moves and M3 moves (see **SI Section 5** for details on the M3 move) (Nobile and Fearnside, 2007) for updating z. The details of the MCMC allocation sampler and the computational complexity are illustrated in **SI Section 1.4**. After sampling the latent label vector z, we then separately sample π from the density p(π|x,z) (See **SI Section 1.2** for the details).

### 2.4 Model fitting

Model fitting procedures are applied at two levels in this paper. At first, we perform BCPD, at an individual level, which we called *global fitting*. Then we perform *local fitting*, at the group level, which is used to estimate the community structure of discrete states. Next, we describe these two model fitting procedures in detail.

#### 2.4.1 Global fitting

*Global fitting* uses a latent block model to fit the adjacency matrix described by a (sliding) window for each time point over the entire time course for each individual. For global fitting, we consider the number of communities, K, in our latent block model to be fixed over the length of the whole experiment. We detect the change-points based on Bayesian model comparison using posterior predictive discrepancy, which does not determine whether the model is ‘true’ or not, but rather quantifies the preference for the model given the data. One can imagine the model as a moving ruler under the sliding window, and the observation at each time step as the object to be measured. The discrepancy increases significantly if there is a change-point located within the window. We repeat the inference with different values of K and compare the performance of our change-point detection method.

#### 2.4.2 Local fitting

*Local fitting* uses a latent block model to fit the adjacency matrix of a discrete brain state at the local minimum of a group-averaged CDE curve. Local fitting involves selecting a model (i.e., choosing a value of K) that best fits the group-averaged adjacency matrix of a discrete brain state. Then we estimate the community memberships that constitute the discrete brain state, which we call *local inference*. We treat K as constant for this local inference (see more details in Section 2.7). The number of communities K can potentially be inferred using the absorption/ejection move (Nobile and Fearnside, 2007) in the allocation sampler, an innovation that will be explored in future research.

### 2.5 Posterior predictive discrepancy

Given inferred values of z and π under the model K, one can draw a replicated adjacency matrix $x^{rep}$ from the predictive distribution $P(x^{rep}|z,\pi ,K)$ as shown in Fig. 1c. Note that the realized adjacency matrix (i.e., an observation) and the replicated adjacency matrix are conditionally independent,

(2.1) $$P\left(x,x^{rep}|z,\pi ,K\right)=P\left(x^{rep}|z,\pi ,K\right)P\left(x|z,\pi ,K\right).$$

Multiplying both sides of this equality by P(z,π|x,K)/P(x|z,π,K) gives

(2.2) $$P\left(x^{rep},z,\pi |x,K\right)=P\left(x^{rep}|z,\pi ,K\right)P\left(z,\pi |x,K\right).$$

Here we use a replicated adjacency matrix in the context of posterior predictive assessment (Gelman et al., 1996) to evaluate the fitness of a posited latent block model to a realized adjacency matrix. We generate a replicated adjacency matrix by first drawing samples (z, π) from the joint posterior P(z,π|x,K). Specifically, we sample the latent label vector z from p(z|x,K) and model parameter π from p(π|x,z) and then draw a replicated adjacency matrix from $P(x^{rep}|z,\pi ,K)$. We compute a discrepancy function to assess the averaged difference between the replicated adjacency matrix $x^{rep}$ and the realized adjacency matrix x, as a measure of model fitness.

In Gelman et al. (1996), the $\chi^{2}$ function is used as the discrepancy measure, where the observation is considered as a vector. However, in the latent block model, the observation is a weighted adjacency matrix and the sizes of the sub-matrices can vary. In this paper, we propose a new discrepancy index to compare adjacency matrices $x^{rep}$ and x. We define a disagreement index to evaluate the difference between the realized adjacency matrix and the replicated adjacency matrix. This disagreement index is denoted by $\gamma (x^{rep};x)$ and can be considered as a parameter-dependent statistic. In mathematical notation, the disagreement index γ is defined as

(2.3) $$\gamma \left(x^{rep};x\right)=\frac{\sum_{i=1,j=1}^{N}|x_{ij}-x_{ij}^{rep}|}{N^{2}},$$

For the evaluation of model fitness, we generate S replicated adjacency matrices and define the posterior predictive discrepancy index (PPDI) $\bar{\gamma }$ as follows.

(2.4) $$\bar{\gamma }=\frac{\sum_{i=1}^{S}\gamma \left(x^{rep^{i}};x\right)}{S}.$$

The computational cost of the posterior predictive discrepancy procedure in our method depends mainly on two aspects. The first is the iterated Gibbs and M3 moves used to update the latent label vectors. The computational cost of these moves is discussed in **SI Section 1.4**. The second aspect is the number of replications S needed for the predictive process. Posterior predictive assessment is not sensitive to the replication number S, but S linearly impacts the computational cost, that is, the computational complexity of model fitness assessment is O(S). There is a natural trade-off between increasing the replication number and reducing the computational speed.

### 2.6 Cumulative discrepancy energy

Our proposed strategy to detect network community change-points is to assess the fitness of a latent block model by computing the posterior predictive discrepancy index (PPDI) ${\bar{\gamma }}_{t}$ for each $t\in \{\frac{W}{2}+1,\ldots ,T-\frac{W}{2}\}$. The key insight here is that the fitness of the model is relatively worse when there is a change-point within the window used to compute $x_{t}$. If there is a change-point within the window, the data observed in the left and right segments are generated by different network architectures, resulting in poor model fit and a correspondingly high posterior predictive discrepancy index.

In practice, we find that the PPDI fluctuates severely. To identify the most plausible position of a change-point, we use another window with window size $W_{s}$ to accumulate the PPDI time series. We obtain the cumulative discrepancy energy (CDE) E(t), given by

(2.5) $$E\left(t\right)=\sum_{i=t-\frac{W_{s}}{2}}^{t+\frac{W_{s}}{2}-1}{\bar{\gamma }}_{i}.$$

We take the locations of change-points to be the local maxima of the cumulative discrepancy energy, where those maxima rise sufficiently high above the surrounding sequence. The change-point detection algorithm is summarized in **SI Section 6.1**.

Note that the posterior predictive discrepancy index and cumulative discrepancy energy for change-point detection are calculated under the conditions of global fitting. For group analysis, we average CDE curves across subjects to obtain the group-averaged CDE. The resulting group-averaged CDE may contain obvious false positives (FP) of local extrema, when multiple local minima or local maxima are located very close together. These false positives are removed using a time distance threshold τ (See **SI Section 6.2** for the proposed algorithm). There is a trade off between the value of τ and the number of false positives removed. A large value of τ can remove as many false positives, but may result in false negatives. In contrast, a small value of τ may not discard enough false positives. After discarding false positives, a change-point is taken to be at each local maximum and a discrete state is inferred from the data segment in a window whose center time point is located at each local minimum of group-averaged CDE.

### 2.7 Discrete brain state and local inference

In this paper, a discrete brain state is defined as a network of static community structure in a time interval between two estimated change-points. The issue is how to determine the correlation matrix corresponding to a discrete brain state? The correlation matrix, corresponding to a discrete brain state, can be calculated from the data segment between two change-points. However, different task conditions may have different block sizes (e.g., the length of fixation block is usually smaller than an n-back block in a working memory paradigm), hence the distance between two consecutive estimated change-points also varies. In order to make a fair comparison between inferred networks of regions corresponding to different task conditions, we define the data segments of task states to have the same length. Specifically, we use the data within a window $W_{l}$, whose center point is located at the estimated local minimum of the CDE curve. The reason for choosing a local minimum as the center of the window is that this time point represents the best fit of the model and data, since it reflects data generated from a putative single brain state. The length of the window $W_{l}$ is chosen to be the same as the length of the shortest block in the paradigm. In brief, we assume that every time point, between two consecutive change-points, is the observation of the same discrete brain state estimated from the data in $W_{l}$. We then use local fitting to select K using the latent block model for estimating community structure for each brain state.

### 2.8 Working memory task fMRI data processing

#### 2.8.1 tfMRI data acquisition

We used working memory task fMRI data from 100 unrelated adults participating in the Human Connectome Project (HCP) (Barch et al., 2013). All participants provided informed consent, and no additional institutional review board (IRB) approval is required. The whole brain echo-planar imaging (EPI) was acquired with a 32 channel head coil on a modified 3T Siemens Skyra with TR = 0.72 s, TE = 33.1 ms, flip angle = 52 degrees, BW = 2290 Hz/Px, in-plane FOV = 208 × 180 mm, 72 slices with isotropic voxels of 2 mm with a multi-band acceleration factor of 8. Two runs of the tfMRI were acquired (one right to left, the other left to right). The original experiment involved a version of an N-back task, used to assess working memory/cognitive control. In the working memory task, each block of tasks consisted of trials with pictures of faces, places, tools and body parts. A specific stimulus type was presented in each block within each run. In 2-back blocks, the subjects judged whether the current stimulus is the same as the stimulus previously presented “two back”. In 0-back blocks, the subjects were given a target cue at the beginning of each task block, and judged whether any stimulus during that block is the same as the target cue. There were 405 frames (with 0.72 s repetition time - TR) in the time course with four blocks of 2-back working memory tasks (each for 25 s), four blocks of 0-back working memory tasks (each for 25 s) and four fixation blocks (each for 15 s).

#### 2.8.2 tfMRI data preprocessing

The tfMRI data in HCP are minimally preprocessed including gradient unwarping, motion correction, fieldmap-based EPI distortion correction, brain-boundary-based registration of EPI to structural T1-weighted scan, non-linear (FNIRT) registration into MNI152 space, and grand-mean intensity normalization. The data analysis pipeline is based on FSL (FMRIB’s Software Library) (Smith et al., 2004). Further smoothing processing is conducted by Volume-based analysis and Grayordinates-based analysis, the details of which are illustrated in the corresponding sections of Barch et al. (2013).

#### 2.8.3 GLM analysis

The general linear model (GLM) analysis in this work includes 1st-level (individual scan run), 2nd-level (combining multiple scan runs for an individual participant) and 3rd-level (group analysis across multiple participants) analyses (Woolrich, Behrens, Beckmann, Jenkinson, Smith, 2004, Woolrich, Ripley, Brady, Smith, 2001). At 1st-level, fixed effects analyses are conducted to estimate the average effect size of runs within sessions, where the variation only contains the within-subject variance. At 2nd-level, we also use fixed effects analysis, averaging the two sessions within the individuals. At 3rd-level, mixed effects analyses are conducted, with the subject effect size considered to be random. The estimated mean effect size is across the population and the between subject variance is contained in the group level of GLM. We can set up different contrasts to compare the activation with respect to the memory load or stimulus type. We applied cluster-wise inference and set up the cluster defining threshold (CDT) to be Z = 3.1 (P = 0.001) to avoid cluster failure problems as described in Eklund et al. (2016), with a family-wise error-corrected threshold of P = 0.05.

#### 2.8.4 Time series extraction

We created spheres of binary masks with radius 6 mm (the center of each sphere corresponded to the coordinates of locally maximum z statistics, and the voxel locations of the centers were transferred from MNI coordinates in FSLeyes) and extracted the eigen time series of 35 regions of interest from the 4-D functional images. We obtained 100 sets of time series from 100 unrelated subjects using the same masks.

## 3 Results

Our proposed method is capable of identifying transitions between discrete brain states and infer the patterns of connectivity between brain regions that underlie those brain states by modeling time-varying dynamics in BOLD signal under different stimuli. In this section, we validate our proposed methodology by applying BCPD and network estimation to both synthetic data and real fMRI data. We first use synthetic multivariate Gaussian data for extensive validation and critically evaluate the performance of our change-point detection and sampling algorithms. For real data analysis, we use working memory task fMRI (WM-tfMRI) data from the HCP. We extracted the time series of 35 nodes whose MNI coordinates were determined by significant activations obtained via clusterwise inference using FSL (Smith et al., 2004).

### 3.1 Method validation using synthetic data

In this section, we perform a set of simulations with, (i) various signal to noise ratios (SNRs); (ii) various degrees of inter-individual variation (DIIV) and; (iii) haemodynamic response function (HRF) to validate BCPD and parameter estimation. Each dataset of these simulations is a collection of time series of 100 virtual subjects simulated from a generative model. The simulated states of segments between two true change-points in the synthetic data can be repeating or all different, depending on the setting of the parameters in the generative model. Firstly, we perform simulations with multivariate Gaussian data with different levels of SNR, but without considering the inter-individual variations of community structures between virtual subjects. In the second set of simulation, we use generative models that can characterize the inter-individual variations in community structures by setting up different degrees of inter-individual variation (DIIV) of true latent labels in the generative model. Here, DIIV is defined as the number of nodes that have different label assignments at the subject level. The last set of simulations is performed with multivariate Gaussian data with a haemodynamic response function. The details of the generative model, how to generate these three sets of simulations and, the definition of DIIV are illustrated in **SI Section 8**.

#### 3.1.1 Effect of SNR on Bayesian change-point detection

**Fig. 2:**
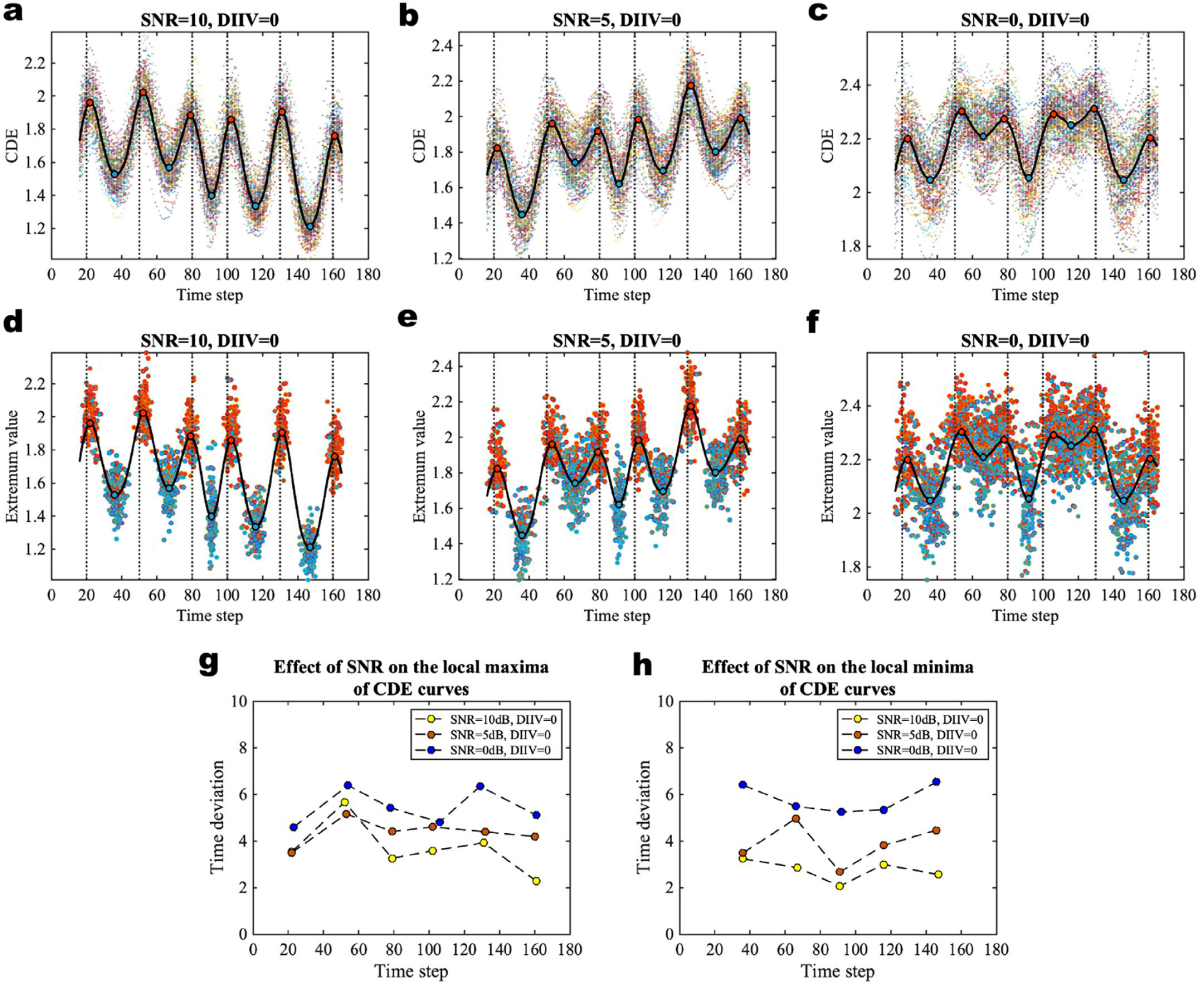
Effect of different levels of SNR on inter-individual variations of CDE curves. **a**–**c** CDE of the multivariate Gaussian data with SNR = 10 dB, 5 dB, and 0 dB respectively. Here, the degree of inter-individual variation (DIIV) of community structure is 0 and the dataset is simulated without HRF. The number of communities is K = 6 for all of the experiments in this figure and the black plot is the group-averaged CDE curve. **d**–**f** The extrema of the individual-level CDE curves with different levels of SNR. The red dots are the local maxima and the blue dots are the local minima of 100 virtual subjects. The black plot is the group-averaged CDE curve. **g** The time deviation of local maxima of individual-level CDE curves compared to the local maximum of the group-averaged CDE curve with different levels of SNR. **h** The time deviation of local minima of individual-level CDE curves compared to the local minimum of the group-averaged CDE curve with different levels of SNR. (For interpretation of the references to color in this figure legend, the reader is referred to the web version of this article.)

We first perform simulations with various SNRs (for further details see **SI Section 8.1**) to evaluate the effect of different levels of SNR on the performance of our BCPD algorithm. There is no inter-individual variation of community structure between subjects (DIIV = 0) and no HRF, so we can ensure that the performance of BCPD will only be affected by SNR in this experiment. We first apply the change-point detection algorithm to each subject to obtain the individual-level CDE curves via global fitting. The group-level CDE curve is calculated by averaging over the individual-level CDE curves. The resulting CDE curves using different levels of SNR and a latent block model with the number of communities K = 6 are shown in Fig. 2a–c. The multi-color scatter plots show the CDE of individuals and the black solid curve is the group-averaged CDE. The local maxima (red dots) of the group-averaged CDE indicate the locations of change-points and the local minima (blue dots) correspond to the center of the windows that form the distinct states that differ in their community architectures. The local maxima and local minima of individual-level CDE curves are shown in Fig. 2d–f with SNR = 10 dB, 5 dB, and 0 dB respectively. We find that there are obvious inter-individual variations between CDE curves and their local extrema for different levels of SNR. To quantify the effect of SNR on this variation, we calculated the time deviation between the individual-level local extrema and group-averaged local extremum. For each group-averaged local extremum (a local maximum or local minimum), we calculated the averaged horizontal time distance between individual extrema and the group-averaged local extremum in a segment between time points of two neighbouring extrema. Fig. 2g and h show the effect of SNR on the time deviations of local maxima and local minima respectively. We find that smaller SNR increases the inter-individual variations between CDE curves.

**Fig. 3:**
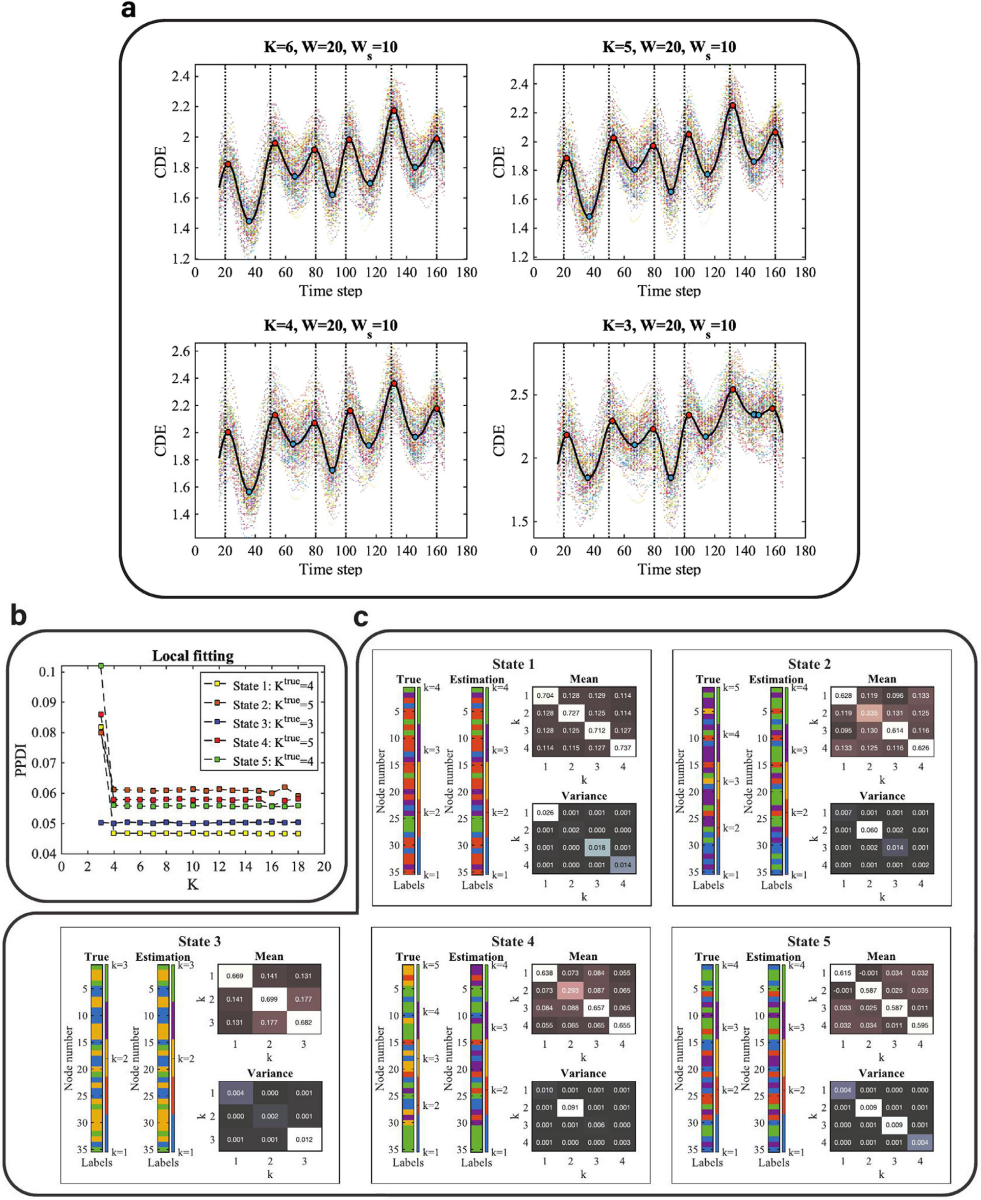
Results of change-point detection with different values of K and local inference. **a** CDE of the multivariate Gaussian data with SNR = 5 dB using different models (K = 6, 5, 4, and 3). The sliding window size for converting from time series to correlation matrices sequence is W = 20, whereas (for smoothing) the sliding window size for converting from PPDI to CDE is $W_{s}$ = 10. The vertical dashed lines are the locations of the true change-points (t = 20, 50, 80, 100, 130, and 160). The multi-color scatterplots in the figures are the CDEs of individual (virtual) subjects and the black curve is the group-level CDE (averaged CDE over 100 subjects). The red dots are the local maxima and the blue dots are the local minima. **b** Local fitting with different models (from K = 3 to 18) for synthetic data (SNR = 5 dB). Different colors represent the PPDI values of different states with the true number of communities $K^{true}$. **c** The estimation of community constituents for SNR = 5 dB at each discrete state: t = 36, 66, 91, 116, 146 for brain states 1 to 5, respectively. The estimations of the latent label vectors (**Estimation**) and the label vectors (**True**) that determine the covariance matrix in the generative model are shown as bar graphs. The strength and variation of the connectivity within and between communities are represented by the block mean and variance matrices within each panel. (For interpretation of the references to color in this figure legend, the reader is referred to the web version of this article.)

Next, we analyse the effects of choosing the number of communities K of latent block model on the performance of BCPD. We demonstrate the results with SNR = 5 dB in the main text. Further simulation results with SNR = 10 dB, SNR = 0 dB, and SNR = -5 dB are provided in **SI Figures 1, 2, and 3**. The resulting cumulative discrepancy energy (CDE) scores using models with different values of K are shown in Fig. 3a. We use a latent block model to fit the adjacency matrix at consecutive time points for change-point detection, which we call global fitting. We find that the local maxima (red dots) are located very close to the true change-points in all of the graphs (in Fig. 3a) which means that the global fitting has good performance for K = 3, 4, 5, and 6 at SNR = 5 dB. Here we clarify that global fitting is used to estimate the locations of the change-points or transitions of brain states, and local fitting is used to select a latent block model to estimate the community structures of discrete brain states (refer to Section 2.4 for the detailed explanation of global and local fitting).

Using the global fitting results, with K = 6 and W = 20, where W is the width of the sliding (rectangular) window, we find the local minima (the blue dots) locations to be t = {36, 66, 91, 116, 146}, where each location corresponds to a discrete state. Next, we use local fitting to select a model (i.e. K for local inference) to infer the community memberships and model parameters relating to the connectivity of the discrete states. For local inference, the group-averaged adjacency matrix is considered as the observation. We assess the goodness of fit between observation and a latent block model with various values of K (from K = 3, ... , 18) using posterior predictive discrepancy for each local minimum, as shown in Fig. 3b. We selected the value of K at which the curve starts to flatten as the preferred model. We find that the model assessment curves for states 1, 2, 4, and 5 flatten at K = 4, whereas the model assessment curve for state 3 is flat over the entire range (from K = 3 and up). Therefore the selected models are K = {4, 4, 3, 4, 4}for states 1 to 5, respectively.

To validate the MCMC sampling of the density p(z|x,K), we compare the estimate of the latent label vector to the ground truth of the node memberships. Fig. 3c shows the inferred community architectures of the discrete states including the estimated latent label vectors and the model parameters of block mean and variance. The true label vectors that determine the covariance matrix in the generative models are also included in this figure. We use the most frequent latent label vectors in the Markov chain after the burn-in steps as the estimate. Note that *label-switching* occurs in the MCMC sampling, which is a well-known problem in Bayesian estimation of mixture models (Stephens, 2000). In the results presented here, the node memberships have been relabelled to correct for label switching. The algorithm used for this purpose is described in **SI Section 7**. We find that the estimated latent label vectors are (largely) consistent with the ground truth of labels that determined the covariance matrix. The discrepant ‘True’ and ‘Estimation’ patterns with respect to states 2 and 4 are due to the bias induced by the selected model (K = 5 for the ground truth and K = 4 for the selected model). Although the colors of the labels in the ‘True’ and ‘Estimation’ patterns are discrepant, we can see that the values of the labels are largely consistent, with some labels of k = 5 missing in the ‘Estimation’ pattern compared to the ‘True’ pattern.

Given the estimated latent label vector, we then draw samples of the block mean and variance from the posterior p(π|x,z) conditional on the estimated latent label vector z. However, there is no ground truth for the block mean and variance when we generate the synthetic data. In order to evaluate the estimation of block mean and variance, we first simulate a synthetic adjacency matrix with ground truth of block mean and variance, then we estimate the block mean and variance by drawing samples from the posterior density to validate the sampling algorithm. The synthetic adjacency matrix and the validation of sampling model mean and variance are illustrated in **SI Figure 4**.

#### 3.1.2 Effects of inter-individual variations of community structures and HRF on Bayesian change-point detection

**Fig. 4:**
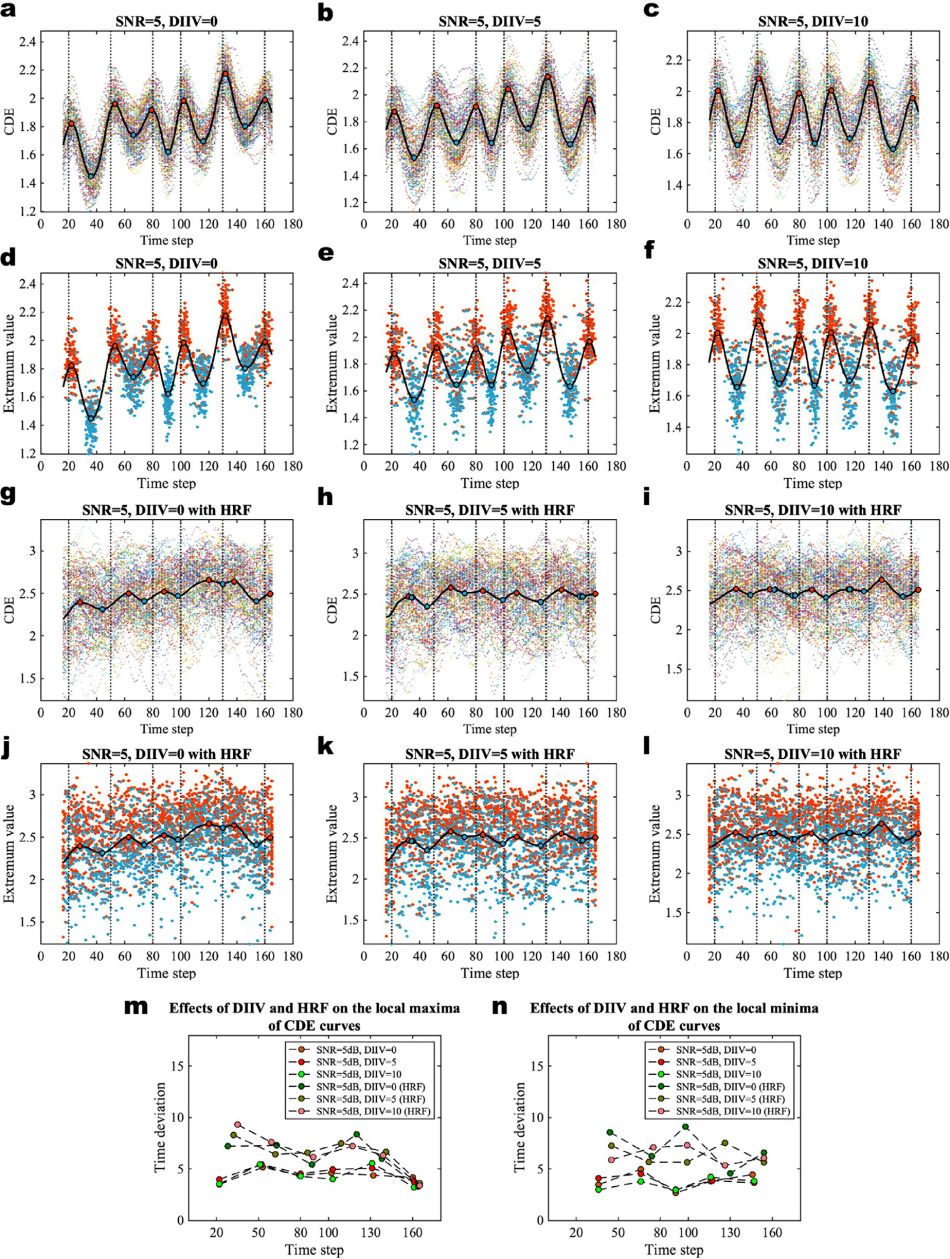
Effects of DIIV and HRF on the inter-individual variations of CDE curves. **a**–**c** CDE of the multivariate Gaussian data with DIIV = 0, 5, and 10 respectively. The SNR = 5 dB and the number of communities K = 6 for all experiments. **d**–**f** The extrema of the individual-level CDE curves with different levels of DIIV. The red dots are the local maxima and the blue dots are the local minima of 100 virtual subjects. **g**–**i** CDE curves of the multivariate Gaussian data applied with haemodynamic response function (HRF). **j**–**l** The extrema of the individual-level CDE curves with HRF. **m** The time deviation of local maxima of individual-level CDE curves compared to the local maximum of the group-averaged CDE curve with different levels of DIIV and HRF. **n** The time deviation of local minima of individual-level CDE curves compared to the local minimum of the group-averaged CDE curve with different levels of DIIV and HRF. (For interpretation of the references to color in this figure legend, the reader is referred to the web version of this article.)

In this section, we evaluate the effects of inter-individual variations of community structures and HRF on the variations of CDE curves. We use a set of experiments (by varying DIIV, but without HRF) simulated from the generative model with DIIV = 0, 5, and 10 respectively, and we take SNR = 5 dB for all of the experiments in this section. The simulation results of evaluating the effects of DIIV and HRF using datasets of SNR = 10 dB and 0 dB are provided in **SI Figures 5 and 6**. The CDE curves and local extrema are shown in Fig. 4a–c and d–f respectively. To imitate the empirical working memory task fMRI data, we perform another set of experiments to evaluate the effect of applying HRF, along with varying DIIV, on our inference method. The experimental results are shown in Fig. 4g–l. We also demonstrate the effect of HRF on the inter-individual variations in CDE by comparing time deviations of local extrema by varying DIIV without HRF and also by varying DIIV and with HRF in Fig. 4m and n.

Results obtained for simulations by varying DIIV, but without HRF, show that both the variations of CDE curves in Fig. 4a–c and the deviations of local extrema in Fig. 4d–f are very small. The results of the horizontal time deviation for SNR = 5 dB and by varying DIIV = 0, 5, and 10 also show very similar values for both local maxima in Fig. 4m and local minima in Fig. 4n. These results indicate that there is almost no effect of DIIV on the inter-individual variation of CDE curves.

For simulated experiments by varying DIIV and with HRF, we find that the CDE curves show appropriate lags in Fig. 4g–i, although some of the lags occur irregularly. The corresponding local extrema become more disperse as shown in Fig. 4j–l compared to the results for simulations in Fig. 4d–f. In Fig. 4m and n, we see that the time deviations of local extrema of experiments with HRF are larger than those without.

Therefore, we draw the conclusion that the inter-individual variations of CDE are mainly due to the different SNR levels and HRF. A smaller SNR and applying HRF will increase the inter-individual variations in CDE. Applying HRF to time series results in appropriate lags (sometimes the local extrema occur irregularly) of CDE curves, we find that there are almost no effects of inter-individual variations of community structures on the inter-individual variations in CDE.

**Fig. 5:**
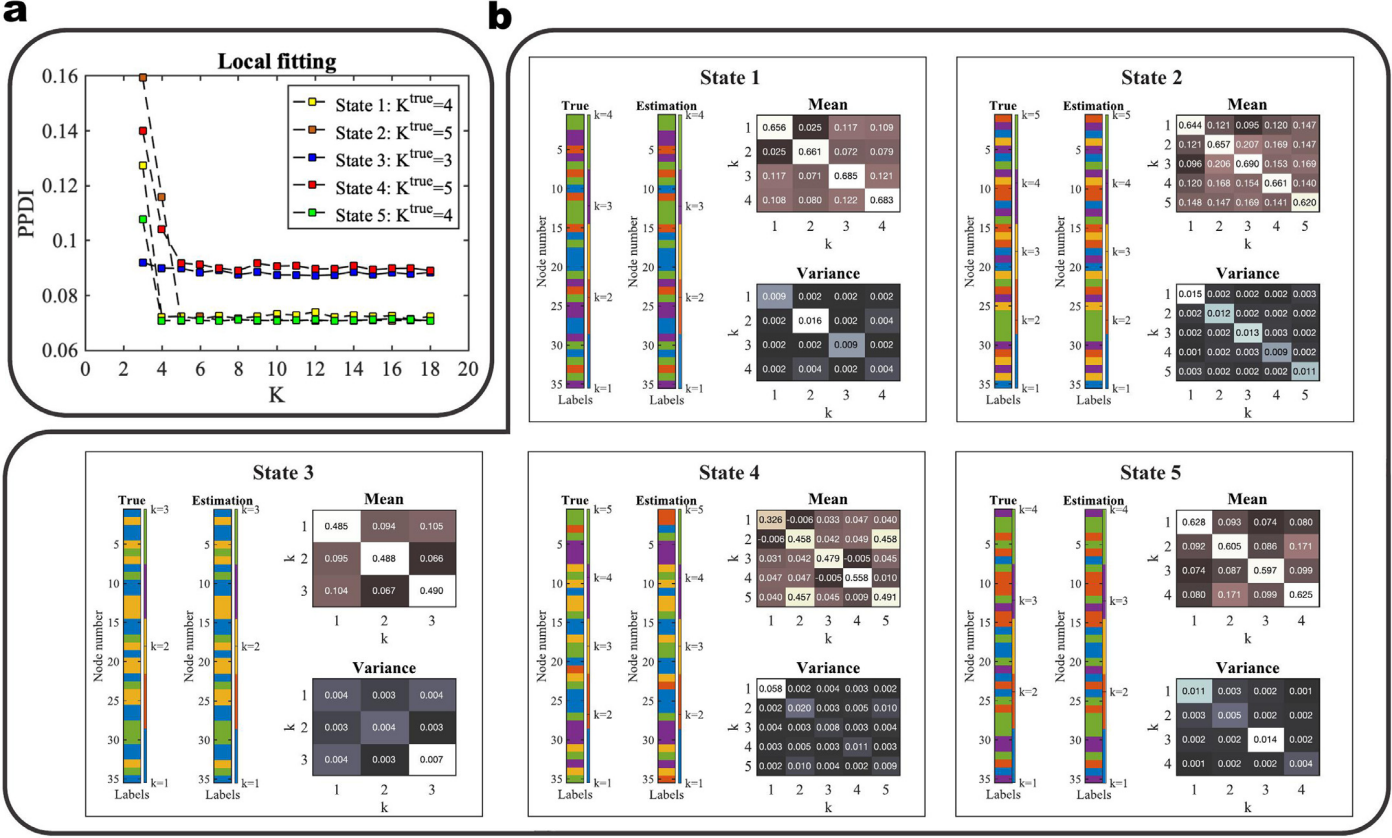
Results of local inference for the multivariate Gaussian data with HRF. **a** Local fitting with different models (from K = 3 to 18) for synthetic data (SNR = 5 dB) with HRF. Different colors represent the PPDI values of different states with the true number of communities $K^{true}$. **b** The estimation of community constituents for the data with HRF at each discrete state, the centres of the state window are t = 44, 74, 98, 130, 154 for brain states 1 to 5, respectively. The estimations of the latent label vectors (**Estimation**) and the label vectors (**True**) that determine the covariance matrix in the generative model are shown as colored bars. The strength and variation of the connectivity within and between communities are represented by the block mean and variance matrices within each panel.

Next, we evaluate whether the performance of local inference is affected by HRF. We demonstrate the results of local inference using SNR = 5 dB, DIIV = 0, and with HRF as shown in Fig. 5. We find that the estimation of K using local fitting in Fig. 5a reflects the ground truth $K^{true}$ accurately. The estimates of latent labels from states 1 to 5 are also largely consistent with the true label vectors in the generative models with synthetic data. These results indicate that applying HRF does not reduce the accuracy of local inference.

**Fig. 6:**
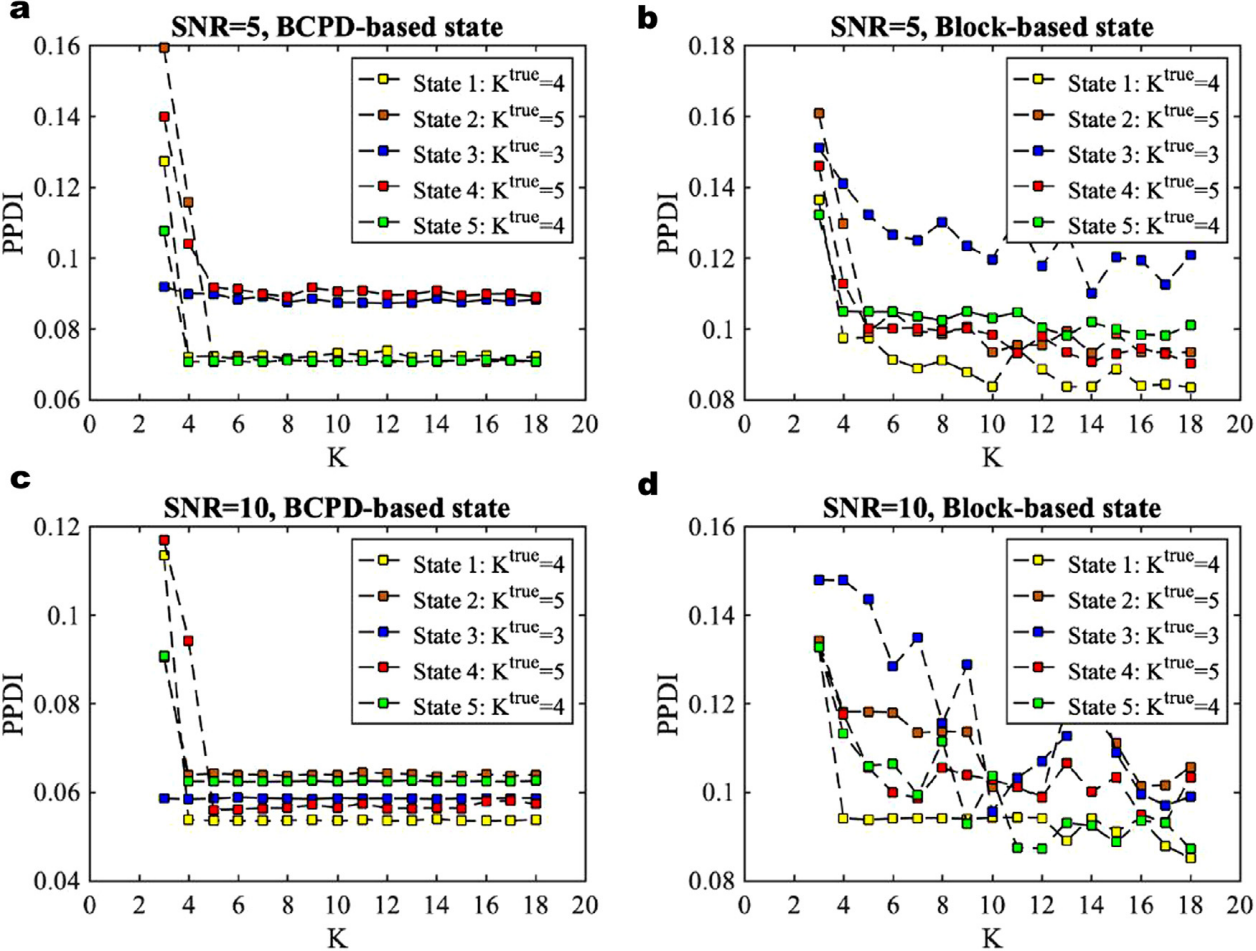
Comparison of the sensitivity of model fitness of BCPD-based states with that of block-based states using synthetic data. **a** Local fitting using the group-averaged adjacency matrix of BCPD-based discrete states (States 1 to 5 at time points t = 44, 74, 98, 130, 154) with HRF and SNR =5 dB. **b** Local fitting using the group-averaged adjacency matrix of block-based states (States 1 to 5 at time points t = 35, 65, 90, 115, 145) with HRF and SNR = 5 dB. **c** Local fitting using the group-averaged adjacency matrix of BCPD-based discrete states (States 1 to 5 at time points t = 43, 75, 98, 125, 154) with HRF and SNR =10 dB. **d** Local fitting using the group-averaged adjacency matrix of block-based states (States 1 to 5 at time points t = 35, 65, 90, 115, 145) with HRF and SNR = 10 dB. Different colors represent the PPDI values of different states with the true number of communities $K^{true}$. All experiments use the window $W_{l}$ = 20 for calculating the adjacency matrices.

For estimating the community structure in task block-designed experiment, one may consider to directly infer the community structure given the known time boundaries of the task blocks; i.e., we calculate the adjacency matrices within the blocks directly without Bayesian change-point detection. Although the block-designed task-based fMRI time series provides the time boundaries of the task blocks which can be considered as the reference points of the locations of the latent brain states, these time boundaries may not represent the exact latent states. For example, the delay induced by the HRF as shown in our previous experiments. The question is that it is not clear how much delay causes differences in the inter-regional correlations and thus community structure. As the community structure is (highly) dependent on the estimation of the number of communities, here we evaluate the sensitivity of the community structure due to the errors in detection of delay. For doing this, we compare the sensitivity of model fitness using group-averaged adjacency matrix of BCPD-based states with that using the adjacency matrix of block-based states. We evaluate the sensitivity of model fitness using synthetic data with SNR = 5 dB. The differences of estimation of K using BCPD-based states and block-based states where the adjacency matrix is calculated from the window $W_{l}$, whose center point is located at the middle of the block, are shown in Fig. 6a and b. We find that the estimation of K of BCPD-based states is K = {4, 5, 3, 5, 4}, which is consistent with the ground truth. Although the estimation of K of block-based states is also largely consistent with the ground truth, except State 3 which is not consistent enough, we can see that the overall values of PPDI in Fig. 6a are smaller than those in Fig. 6b, which means that the estimation of the number of communities using the BCPD-based discrete states is more accurate and less noisy than that using the block-based adjacency matrices. The results of the comparison using synthetic data with HRF (SNR = 10 dB) are shown in Fig. 6c and d. We observed a similar difference of sensitivity of model fitness as we did for SNR = 5 dB. Overall, we conclude that the estimation of the number of communities of the discrete states is more accurate by using BCPD than when using the block-based states. For block-based states, the data segment within the window located at the center of the task block may have been generated from two latent network architectures due to the error of delays caused by HRF, which will in turn result in the worse model fitness.

### 3.2 Method validation using working memory (WM) task-fMRI data

In this analysis, we used preprocessed working memory (WM)-tfMRI data obtained from 100 unrelated healthy adult subjects under a block designed paradigm, available from the Human Connectome Project (HCP) (Barch et al., 2013). We mainly focused on the working memory load contrasts of 2-back vs. fixation, 0-back vs. fixation, or 2-back vs. 0-back, and determine the brain regions of interest from the GLM analysis. After group-level GLM analysis, we obtained cluster activations with locally maximum Z statistics for different contrasts. The results in the form of thresholded local maximum Z statistic (Z-MAX) maps are shown in **SI Figure 7**. The light box views of thresholded local maximum Z statistic with different contrasts are provided in **SI Figure 8**. Significant activations obtained by clusterwise inference and the corresponding MNI coordinates with region names are shown in Table 1. We finally extracted the time series of 35 brain regions corresponding to the MNI coordinates. Refer to Section 2.8 for the details of experimental design, GLM analysis and time series extraction.

**Table 1:** Significant activations of cluster wise inference (cluster-corrected Z > 3.1, P < 0.05). Activations are described in terms of local maximum *Z* (*Z*-MAX) statistic within each cluster including the activations of all contrast maps among 2-back, 0-back, and fixation.

		MNI coordinates			Voxel locations			
Node number	*Z*-MAX	*x*	*y*	*z*	*x*	*y*	*z*	Region name
1	4.97	48	−58	22	21	34	47	Angular Gyrus
2	9.61	36	8	12	27	67	42	Central Opercular Cortex
3	8.27	−36	4	12	63	65	42	Central Opercular Cortex
4	6.48	40	34	−14	25	80	29	Frontal Orbital Cortex
5	7.83	−12	46	46	51	86	59	Frontal Pole
6	4.84	54	32	−4	18	79	34	Inferior Frontal Gyrus
7	6	52	38	10	19	82	41	Inferior Frontal Gyrus
8	4.38	−52	40	6	71	83	39	Inferior Frontal Gyrus
9	6.05	52	−70	36	19	28	54	Inferior Parietal Lobule PGp R
10	7.26	−48	−68	34	69	29	53	Inferior Parietal Lobule PGp L
11	6.18	44	−24	−20	23	51	26	Inferior Temporal Gyrus
12	9.54	36	−86	16	27	20	44	Lateral Occipital Cortex
13	8.04	−30	−80	−34	60	23	19	Left Crus I
14	7.6	−8	−58	−52	49	34	10	Left IX
15	6.9	−22	−48	−52	56	39	10	Left VIIIb
16	14.5	6	−90	−10	42	18	31	Lingual Gyrus
17	10.3	30	10	58	30	68	65	Middle Frontal Gyrus
18	6.61	66	−30	−12	12	48	30	Middle Temporal Gyrus
19	4.53	−68	−34	−4	79	46	34	Middle Temporal Gyrus
20	14.5	18	−88	−8	36	19	32	Occipital Fusiform Gyrus
21	5.06	−12	−92	−2	51	17	35	Occipital Pole
22	9.87	6	40	−6	42	83	33	Paracingulate Gyrus
23	12	42	−16	−2	24	55	35	Planum Polare
24	11.3	−40	−22	0	65	52	36	Planum Polare
25	9.03	38	−26	66	26	50	69	Postcentral Gyrus
26	8.31	−10	−60	14	50	33	43	Precuneus Cortex
27	5.7	46	−60	−42	22	33	15	Right Crus I
28	8.34	32	−80	−34	29	23	19	Right Crus I
29	10.9	32	−58	−34	29	34	19	Right Crus I
30	6.41	10	−8	−14	40	59	29	Right Hippocampus
31	6.19	32	−52	2	29	37	37	Right Lateral Ventricle
32	7.69	24	−46	16	33	40	44	Right Lateral Ventricle
33	6.13	0	10	−14	45	68	29	Subcallosal Cortex
34	10.7	48	−44	46	21	41	59	Supramarginal Gyrus
35	4.23	−50	−46	10	70	40	41	Supramarginal Gyrus

#### 3.2.1 Change-point detection for tfMRI time series

**Fig. 7:**
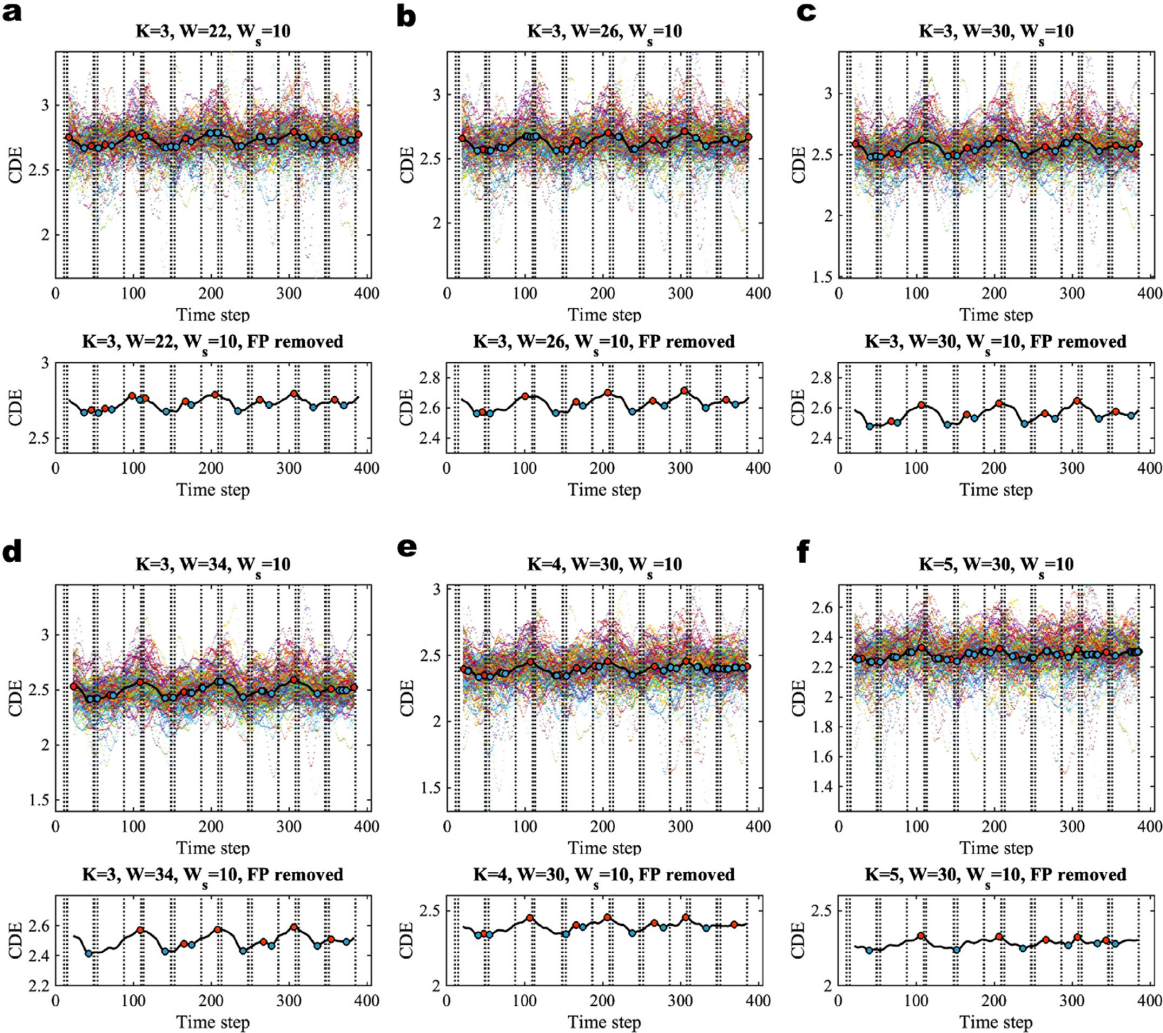
The results of BCPD for working memory tfMRI data (session 1, LR). The upper panels are the cumulative discrepancy energy (CDE) with different sliding window sizes (W = 22, 26, 30, and 34; **a**–**d** under the model K = 3) and different models (K = 3, 4, and 5; **c, e** and **f** using a sliding window of W = 30). $W_{s}$ is width of the sliding window used for converting from PPDI to CDE. The vertical dashed lines are the times of onset of the stimuli (which were provided in the EV.txt files in the released data). The multi-color scatter plots in the figures represent the CDEs of individual subjects and the black curve is the group-level CDE (averaged over 100 subjects). The red dots are the local maxima, which are taken to be the locations of change-points, and the blue dots are the local minima, which are used for local inference of the discrete brain states. The bottom panels show the estimated group-averaged CDE where false positives (FP) are removed using time distance threshold τ = 7. (For interpretation of the references to color in this figure legend, the reader is referred to the web version of this article.)

In the main text, we illustrate the results using the HCP working memory data of session 1, i.e. with the polarity of Left to Right (LR). The replication of results obtained by using session 2 (RL) are demonstrated in **SI Figures 12 to 17** and **SI Table 1**. We compare the brain states of different working memory loads for a specific kind of picture (tool, body, face, and place) involved in the experiments. As there is no repetition of task conditions in a single session, the estimated patterns of brain states do not recur in LR session. One can compare the LR and RL session for the recurrence of a specific task condition. To detect change-points in the extracted time series, we first converted each time series into a sequence of correlation matrices for each subject. We then modeled this sequence of correlation matrices for each subject using the latent block model and evaluated posterior predictive discrepancy (PPD) to assess the model fitness. Next, we converted the resulting PPD index (PPDI) to a CDE score for each subject. For group-level analysis, we averaged the resulting individual-level CDE scores over 100 subjects to obtain a sequence of group-averaged CDE as shown in Fig. 7 with different window sizes W = 22, 26, 30, 34 (Fig. 7a–d) and different values of K (Fig. 7c, e, and f). We chose the window size for converting from PPDI to CDE to be a constant $W_{s}$ = 10 for all of the assessments. In the upper panels, the multi-colored scatterplots in the figures are the individual-level CDE scores of 100 subjects, and the black curves are the group-averaged CDE. The bottom panels show the group-averaged CDE curve after removing false positives. With the same number of communities K = 3, we found there are more false positives with window size W = 22 compared to W = 26, W = 30 and W = 34. This is because there are fewer sample data contained in the sliding window if the window size is smaller. We also tried different models with K = 4 and K = 5. We found that there are more false positives with larger values of K. Larger values of K imply more blocks in the model, which gives rise to relatively better model fitness. In this situation, there will be less distinction between relatively static brain states and transition states with change-points in the window. The false positives among the local minima and local maxima are also influenced by the window size $W_{s}$ used for transforming from PPDI to CDE. A larger window size (for example $W_{s}$ = 30) reduces the accuracy of the estimates and results in false negatives. Too small a value of $W_{s}$ increases the false positive rate. We found that $W_{s}$ = 10 works well for all of the real data analyses. Here, we suggest a rule of thumb for choosing the window sizes W and $W_{s}$, and the model K for change-point detection in fMRI data analysis. The window W should be less than or equal to the task blocks, but as large as possible (usually, we select W to be the same as the length of the shortest task block in the paradigm). The window for converting from PPDI to CDE is set to be $W_{s}$ = 10. The model K = 3 is usually appropriate for BCPD for task fMRI data. We use the algorithm provided in **SI Section 6.2** with time distance threshold τ = 7 to remove any false positives. The change-point detection results after removing false positives are provided in the bottom panels. We find that there are still some unexpected local extrema remaining in Fig. 7a, b and e in the first task block and some false negatives in Fig. 7b, d, e and f. The extrema of Fig. 7c best align with the time boundaries of the task blocks.

After removing false positives, we note that the onsets of the stimuli precede the inferred local maxima, and the local minima also show appropriate lags (for example, about 10 frames, or 7 s as shown in Fig. 7c) compared to the mid-points of the working memory blocks. For fixation blocks, the local maxima show lags compared to the mid-points of the blocks. In our results obtained from simulations with HRF, we observed similar lags between the true time boundaries of Gaussian data segments and the time points of estimated extrema. We suggest that the lags, which are estimated using working memory task fMRI data, are likely due to the delay introduced by the haemodynamic response. Our data-driven method based on the latent block model, without any neurobiological constraint, appears sufficient to account for these lags. Nevertheless, the change-point detection algorithm, in combination with generative methods based on the dynamic and biophysical models that explicitly characterize neural dynamical systems (Lurie et al., 2020) might explain if such lags have any neurobiological explanation. Regarding the computational cost, the time spent to run the posterior predictive assessment on each subject (T = 405 frames, posterior predictive replication number S = 50, K = 3, and the window size W = 30) by using a 2.6 GHz Intel Core i7 processor unit was about 10 min.

**Fig. 8:**
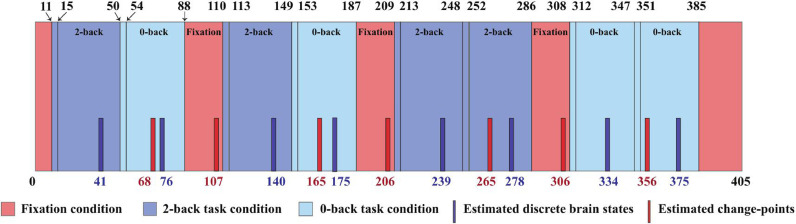
Detected change-points and locations of the windows regarding the brain states matching the task blocks for working memory tfMRI data (session 1, LR) with K = 3, and W = 30. The numbers at the top of rectangles are the boundaries of the external task demands, the rectangles with background colors are the different task conditions, and the blue and red bars with specified numbers are the estimated locations of the windows for the discrete brain states and change-points. (For interpretation of the references to color in this figure legend, the reader is referred to the web version of this article.)

The ‘local inference’ is defined as a way to estimate the discrete brain state via Bayesian modeling. The group-averaged dynamic functional networks were analyzed by performing ‘local inference’ as follows. In this experiment, we used results obtained by change-point detection for K = 3 and W = 30 (see Fig. 7c). The resulting estimated change-point locations (the time points of local maxima) are at t = {68, 107, 165, 206, 265, 306, 356}, and the estimated time points of the windows regarding the discrete brain states (the time points of local minima) are at t = {41, 76, 140, 175, 239, 278, 334, 375}. A summary of comparison between the detected change-points and condition blocks for working memory tfMRI data using W = 30 and K = 3 are shown in Fig. 8.

#### 3.2.2 Local inference for discrete brain states in tfMRI

**Fig. 9:**
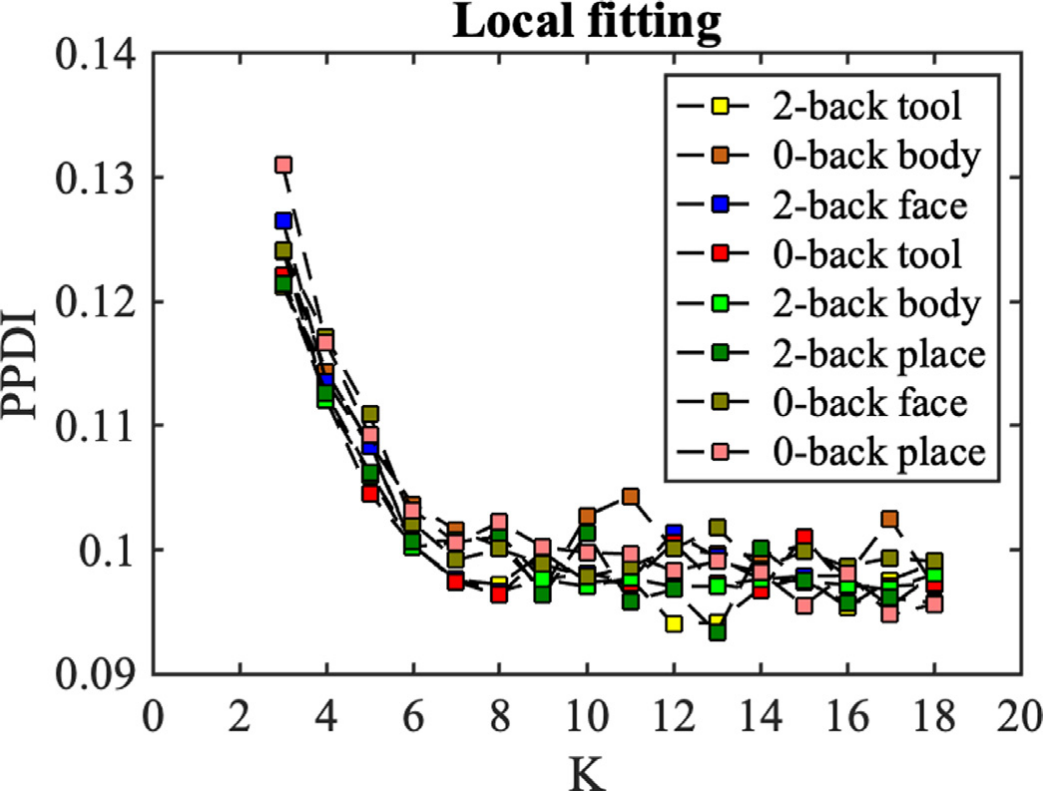
Local fitting between averaged adjacency matrix and models from K = 3 to 18. Different colors represent the PPDI values of different brain states.

For ‘local inference’, we first calculated the group-averaged adjacency matrix with a window of $W_{l}$ = 20, for all brain states. The center of the window is located at the time point of the local minimum value. We evaluated the goodness of fit for models with different values of K (Fig. 9). The results demonstrate that the goodness of fit trends to flat at K = 6. To avoid empty communities, K = 6 is then selected as the number of communities in local inference. Note that the value of K is unchanged in Markov chain Monte Carlo estimation, but an empty community containing no labels may take place. In the remainder of this section, we used the model with K = 6 for all brain states. The times spent to run the estimation for latent label vector and model parameters for a single discrete brain state (MCMC sampling number $S_{s}$ = 200, K = 6, and the window size $W_{l}$ = 20) by using a 2.6 GHz Intel Core i7 processor unit were about 1.85 and 1.25 s respectively.

**Fig. 10:**
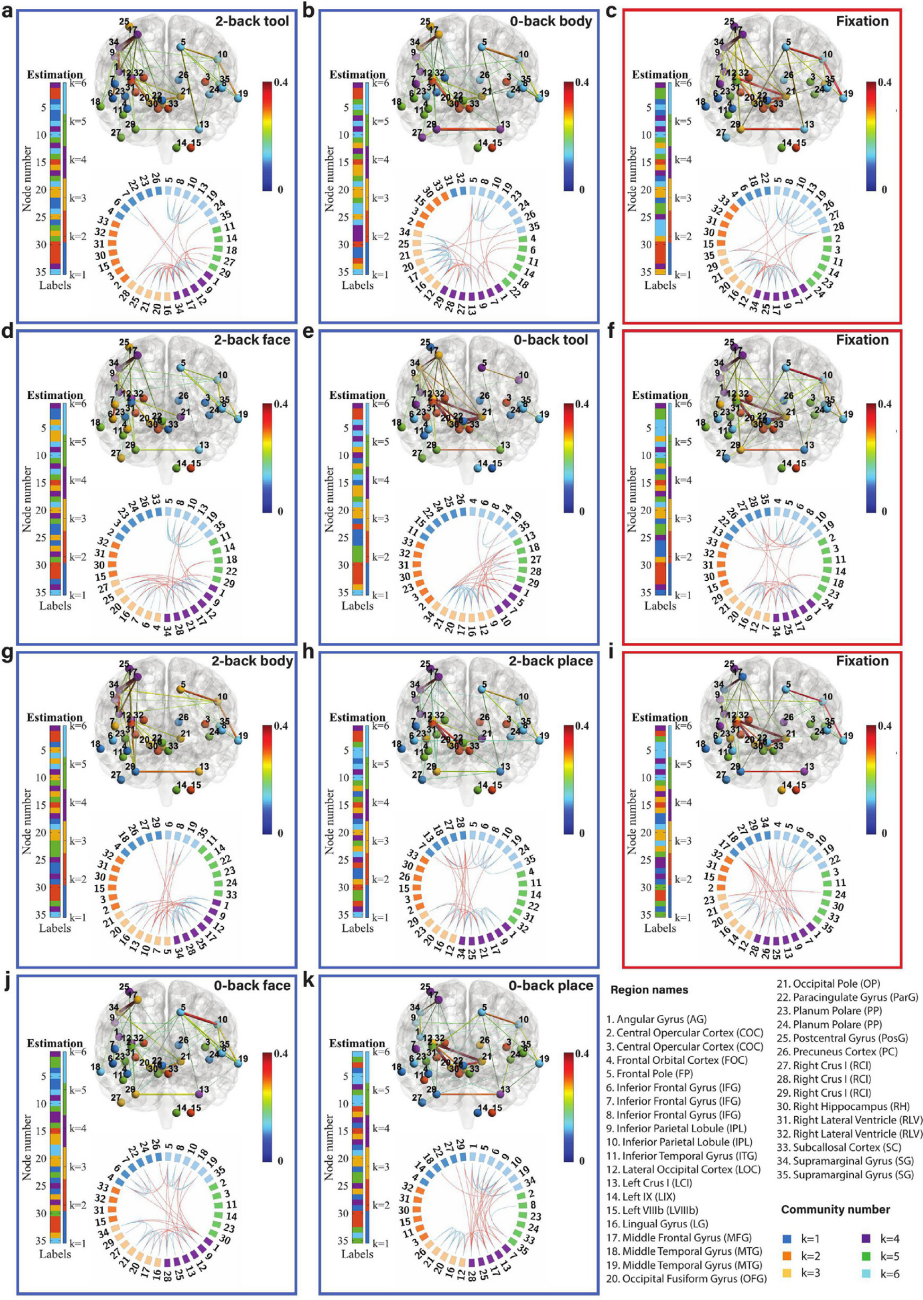
Community structure of the discrete brain states. The figures with blue frames represent brain states corresponding to working memory tasks (2-back tool at t = 41; 0-back body at t = 76; 2-back face at t = 140; 0-back tool at t = 175; 2-back body at t = 239; 2-back place at t = 278; 0-back face at t = 334; and 0-back place at t = 375 in **a**-**k**) and those with red frames represent brain states corresponding to fixation (fixation at t = 107, 206, and 306 in **c, f**, and **i**). Each brain state shows connectivity at a sparsity level of 10%. The different colors of the labels represent community memberships. The strength of the connectivity is represented by the colors shown in the bar at the right of each frame. In Circos maps, nodes in the same community are adjacent and have the same color. Node numbers and abbreviations of the corresponding brain regions are shown around the circles. In each frame, different colors represent different community numbers. The connectivity above the sparsity level is represented by arcs. The blue links represent connectivity within communities and the red links represent connectivity between communities. (For interpretation of the references to color in this figure legend, the reader is referred to the web version of this article.)

The inferred community structures are visualized using BrainNet Viewer (Xia et al., 2013) and Circos maps (Krzywinski et al., 2009) as shown in Fig. 10. Estimated latent label vectors are visualized using different colors to represent different communities. The community labels of nodes of different states also show inconsistencies (i.e., the label-switching phenomenon). Here, we used the relabelling algorithm as described in **SI Section 7** to reassign the labels across different states. The nodes are connected by weighted links at a sparsity level of 10% (we also visualized the brain states with sparsity levels of 20% and 30%: **SI Figures 9 and 10**). The density and variation of connectivity within and between communities are characterized by the estimated block mean matrix and block variance matrix in **SI Figure 11** for LR session and **SI Figure 17** for RL session. We first describe the working memory tasks involving the 2-back tool (Fig. 10a), 0-back tool (Fig. 10e), and fixation (Fig. 10c, f, i). The locations of fixation states are considered as the locations of the change-points at 107, 206, and 306 (we consider the fixation state as a transition buffer between two working memory blocks). We found that the connectivity between the inferior parietal lobule (node 9) and middle frontal gyrus (node 17), and the connectivity between the inferior parietal lobule (node 9) and supramarginal gyrus (node 34) are increased significantly both in 2-back and 0-back working memory compared to fixation.

For 2-back face (Fig. 10d) and 0-back face (Fig. 10j), The connectivity between inferior parietal lobule (node 9) and supramarginal gyrus (node 34) and the connectivity between angular gyrus (node 1) and supramarginal gyrus (node 34) are increased in 2-back compared to 0-back and fixation. There is reduced connectivity between the lateral occipital cortex (node 12), occipital fusiform gyrus (node 20), and occipital pole (node 21) in 2-back and 0-back compared to fixation.

For task blocks with body parts pictures (Fig. 10g and b), we found that the connectivity between inferior parietal lobule (node 9) and middle frontal gyrus (node 17), and the connectivity between inferior parietal lobule (node 9) and supramarginal gyrus (node 34) are increased significantly both in 2-back and 0-back working memory compared to fixation. The connectivity between angular gyrus (node 1) and supramarginal gyrus (node 34) is increased in 2-back compared to 0-back and fixation. There is reduced connectivity between the lateral occipital cortex (node 12), occipital fusiform gyrus (node 20), and occipital pole (node 21) in 2-back and 0-back compared to fixation.

Finally, we compare 2-back place (Fig. 10h), 0-back place (Fig. 10k), and fixation. We found that the connectivity between lateral occipital cortex (node 12) and occipital pole (node 21), and the connectivity between occipital fusiform gyrus (node 20) and occipital pole (node 21) are reduced in 2-back compared to 0-back and fixation.

It is clear from Fig. 10 that nodes are clustered into communities with different connectivity densities within and between communities. The mean and variance of the connectivity within and between communities are reported as block mean and variance matrices in **SI Figure 11**. We find that there are strong connections in communities k = 3, 4, and 6 and weak connections in communities k = 1, 2, and 5 for a majority of the states. The Circos map provides a different perspective on the community pattern of the brain state. We summarise the common community pattern for specific working memory load or fixation in Table 2.

**Table 2:** A table of community detection with session 1 (LR). This table summarises the nodes commonly located in a specific community k for all of the picture types in the working memory tasks.

2-back					0-back				Fixation				
Community	Node number				Community	Node number			Community	Node number			
k=1					k=1				k=1				
k=2	15	30			k=2				k=2	15	31	32	
k=3	16	20			k=3	16	20	21	k=3	12	16	20	21
k=4	1	9	17	34	k=4				k=4	1	9	25	
k=5	11	14			k=5				k=5	3	11	14	
k=6	8	19	35		k=6	19			k=6	5	8	10	19

## 4 Discussion

We proposed a Bayesian change-point detection method for identifying transitions and characterising brain states between two consecutive transitions. The method is validated by extensive simulations and block designed task fMRI data with known time boundaries of task blocks. The transitions between brain states identified by the BCPD method exhibit consistency and appropriate lags compared to the external task demands. This indicates the feasibility of BCPD, and also a significant difference between the temporal boundaries of external task demands and the transitions of latent brain states. We also estimated the community memberships of brain regions that interact with each other to give rise to the brain states. Furthermore, we showed that the estimated patterns of community architectures show distinct networks for 2-back and 0-back working memory load and fixation. After validation using working memory task-based fMRI data, the BCPD can be applied to fMRI with naturalistic stimuli (e.g. movie-watching, music listening) where the time boundaries may be uncertain and indistinguishable, or the resting state fMRI which has relatively less distinct switching of brain states. In movie-watching experiment, the sensory and narrative cues may be considered as referential information, while there is no referential information in resting-state fMRI data. The BCPD was just applied to locate the relatively static brain states occurring in block designed task fMRI data. In future work, we aim to apply the method to explore the dynamic characteristics of event-related task fMRI, where applying a sliding window approach may be difficult, as the changes of the states will be the pulses.

The dynamics of the brain states are not only induced by external stimuli, but also the latent mental process, such as motivation, alertness, fatigue, and momentary lapse (Taghia et al., 2018). Crucially, for task-based fMRI, directly using the temporal boundaries (onsets and duration) associated with predefined task conditions to infer the functional networks may not be sufficiently rigorous and accurate. The boundaries of the task demand are not the timing and duration of the latent brain state. The estimated change-points in our experiments are consistent with the working memory task demands but show a delay relative to the onsets of the task blocks or the mid-points of fixation blocks. These results reflect the delay involved due to the haemodynamic response. The delay may also arise from other factors such as neurobiologically plausible delays introduced when performing certain tasks, or recording of the data using the fMRI scanner, and between signal emission and reception.

We defined a new criterion named cumulative discrepancy energy (CDE) to estimate locations of these change-points or transitions. The main idea underlying this novel strategy is to recognize that the goodness-of-fit between the model and observation is reduced if there is a change-point located within the current sliding window (the sample data in the window can be considered as being generated from two latent brain network architectures in this case), resulting in a significant increase in CDE. The individual-level CDE curves are extremely noisy due to the high level of noise in fMRI dataset. In this paper, the group-averaged CDE is proposed as a core criterion to infer the change-points. The unpredictable intrinsic mental process in task-free experiment may have severe effect on the dynamics of brain networks. Averaging over CDE at group level may alleviate the effect of intrinsic cognitive process, but may also neglect the variability between subjects. In future, the hierarchical Bayesian modeling will be proposed for change-point detection, which will evaluate the variability of dynamics of individual latent brain networks for both task-based and task-free fMRI designs.

The posterior predictive discrepancy (PPD) based on model fitness assessment combined with sliding window analysis is proposed to detect change-points in various functional brain networks and to infer the dynamics when a brain changes state. Posterior predictive assessment is a method based on Bayesian model comparison. Other Bayesian model comparison methods including Bayes factors (Kass, Raftery, 1995, West, 1986), the Bayesian information criterion (BIC) (Neath and Cavanaugh, 2012), and Kullback–Leibler divergence (Kullback and Leibler, 1951) are also widely used in mixture modeling. One advantage of the posterior predictive assessment is that the computation for the assessment is a straightforward byproduct of the posterior sampling required in the conventional Bayesian estimation.

We used overlapping, rectangular, and fixed size sliding windows so that all of the time points are included. Although fixed sliding windows are used in this paper, there is another method based on the adaptive forgetting windows which may yield improved performance when applied to change-point detection. In this adaptive windowing method, a time varying window can be learnt automatically (Monti et al., 2017a). We will explore different shapes of windows (e.g., Gaussian windows, tapered window etc), and adaptive windowing in future research.

The results of the task fMRI data analysis show that the change-point detection algorithm is sensitive to the choice of model. We found that a less complex model (with smaller K) for global fitting gave fewer false positives, so it had better change-point detection performance than models with larger K. Selecting a suitable window size W is also very important for our method. Too small a window size results in too little information being extracted from the data within the window, causing the calculated CDE to fluctuate more, making it difficult to discriminate local maxima and local minima in the CDE score time series. Too large a window size (larger than the task block length) reduces the resolution at which the change-points can be distinguished. In the working memory task fMRI dataset, the length of the task block is around 34 frames and the fixation is about 20 frames. Therefore, we made the sliding window size W at most 34 frames to ensure all potential change-points can be distinguished, and at least 20 frames to ensure the effectiveness of the posterior predictive assessment. In our experiments, we used window sizes W = 22, 26, 30, and 34, which were all larger than the length of the fixation block. This means it was not possible to detect the two change-points at both ends of fixation blocks, so we consider the whole fixation block as a single change-point (i.e., a buffer between two task blocks).

Empirical fMRI datasets have no ground truth regarding the locations of latent transitions of the brain states and network architectures. Although the task data experiments include the timings of stimuli, the exact change-points between discrete latent brain states are uncertain. Here, we used the multivariate Gaussian model to generate synthetic data (ground truth) to validate our proposed algorithms by comparing ground truth to the estimated change-points and latent labels. With extensive experiments using synthetic data, we demonstrated the very high accuracy of our method. The multivariate Gaussian generative model can characterize the community patterns via determining the memberships of the elements in the covariance matrix, but it is still an unrealistic benchmark. In the future, we will integrate the clustering method into the dynamic causal modeling (Friston, Harrison, Penny, 2003, Friston, Preller, Mathys, Cagnan, Heinzle, Razi, Zeidman, 2019) to simulate more biologically realistic synthetic data to validate the algorithm.

The latent block model provides a flexible approach to modeling and estimating the dynamical assignment of nodes to a community. Note that the latent block model was fitted to the adjacency matrix of each individual subject in global fitting, and was fitted to the group-averaged adjacency matrix in the local fitting. Different choices of π can generate different connection patterns in the adjacency matrix. The likelihood is Gaussian and the connectivity is weighted, both of which facilitate treating the correlation matrix as an observation, without losing much relevant information from the time series. We treat both the latent label vector and block model parameters as quantities to be estimated. Changes in community memberships of the nodes are reflected in changes in the latent labels, and changes in the densities and variations in functional connectivity are reflected in changes in the model parameters. For modeling brain networks, we used non-informative prior in the latent block model. In the future, we will explore the empirical Bayesian framework (Mejia et al., 2018) for the latent block model at the subject level where the prior is estimated from the data.

There are still some limitations of the MCMC allocation sampler (Nobile, Fearnside, 2007, Wyse, Friel, 2012) which we use to infer the latent label vectors. When Markov chains are generated by the MCMC algorithm, the latent label vectors typically get stuck in local modes. This is in part because the Gibbs moves in the allocation sampler only update one element of the latent label vector at a time. Although the M3 move updates multiple elements of the latent label vector, the update is conditional on the probability ratio of a single reassignment, which results in similar problems to the Gibbs move. Improving the MCMC allocation sampler so that it can jump between different local modes, without changing the value of K, is a topic worth exploring. Currently, we use an MCMC sampler with a Gibbs move and an M3 move for local inference as well, keeping K constant. In future work, we will extend the sampler using an absorption/ejection move, which is capable of sampling K along with latent labels directly from the posterior distribution. The label-switching phenomenon does not happen frequently if the chain is stuck in a local mode. However, the estimated labels in the latent label vector do switch in some experiments. To correct for label switching, we permute the labels in a post-processing stage.

Next, we discuss the results of the brain states inferred from the WM-tfMRI data and discuss the estimated patterns of connectivity for different blocks of working memory tasks after local inference. We find that there are distinct connectivity differences between 2-back, 0-back, and fixation. We first compare the working memory and the fixation conditions, with particular reference to the middle frontal gyrus (node 17) and inferior parietal lobule (node 9) which includes the angular gyrus (node 1) and supramarginal gyrus (node 34). The middle frontal gyrus is related to manipulation, distractor resistance, refreshing, selection for action and monitoring, and the inferior parietal lobule is related to focus recognition and long-term recollection (Nee et al., 2013). In our results, we find that the connectivity between the middle frontal gyrus and inferior parietal lobule is increased in the working memory tasks compared to the fixation state. The connectivity between the lateral occipital cortex (node 12) and occipital fusiform cortex (node 21) is strong and stable in fixation compared to the working memory tasks, and a higher working memory load may increase the instability of this connectivity.

Regarding the difference between 2-back and 0-back working memory tasks, we focus on the angular gyrus and supramarginal gyrus. In our experimental results, we find that there is increased connectivity between the angular gyrus (node 1) and supramarginal gyrus (node 34) in 2-back compared to 0-back working memory task blocks. The angular gyrus is located in the posterior part of the inferior parietal lobule. The inferior parietal cortex, including the supramarginal gyrus and the angular gyrus, is part of a “bottom-up” attentional subsystem that mediates the automatic allocation of attention to task-relevant information (Seghier, 2013). Previous work has shown that activation of the inferior parietal lobe is involved in the shifting of attention towards particular stimuli (Gottlieb, 2007). The right inferior parietal lobule including angular gyrus is related to attention maintaining and salient event encoding in the environment (Singh-Curry and Husain, 2009). These research findings are consistent with and justify our results.

The visualization of discrete brain states in this paper demonstrated the group-averaged connectivity and community structure of brain networks. There are several existing methods that are able to characterize the differences across subjects rather than over time (Monti, Anagnostopoulos, Montana, 2017b, Monti, Hyvärinen, 2018). Evaluating the variability between subjects as well as over time in dynamic functional connectivity is an important topic. In this paper, we only treat the group-averaged adjacency matrix as an observation in local inference, which neglects variation between subjects (Betzel, Bertolero, Gordon, Gratton, Dosenbach, Bassett, 2019, Friston, Litvak, Oswal, Razi, Stephan, Van Wijk, Ziegler, Zeidman, 2016). As a future work, we propose to use hierarchical Bayesian modeling to estimate the community architecture at the group level. In the local inference, we will model the individual adjacency matrix using the latent block model, and infer the number of communities along with the latent label vectors via an absorption/ejection strategy. At the group level, we will model the estimated number of communities of the subjects using a Poisson-Gamma conjugate pair and model the estimated latent label vectors using a Categorical–Dirichlet pair. The posterior distribution of the number of communities will be modeled using a Gamma distribution and the posterior distribution of the latent label vector will be modelled using a Dirichlet distribution. The estimated label assignment probability matrix of the Dirichlet posterior distribution will characterize the brain networks at the group level.

## Code availability

The code for GLM analysis (Shell script), Bayesian change-point detection (MATLAB), and brain network visualization (MATLAB, Perl) is available at: https://github.com/LingbinBian/BCPD1.0.

The pre-processed working memory task fMRI data from Human Connectome Project is available at: https://www.humanconnectome.org/.

## CRediT authorship contribution statement

**Lingbin Bian:** Conceptualization, Methodology, Data curation, Visualization, Software, Formal analysis, Investigation, Validation, Writing – original draft. **Tiangang Cui:** Investigation, Supervision, Writing – review & editing. **B.T. Thomas Yeo:** Writing – review & editing. **Alex Fornito:** Writing – review & editing. **Adeel Razi:** Conceptualization, Methodology, Investigation, Validation, Funding acquisition, Project administration, Resources, Supervision, Writing – review & editing. **Jonathan Keith:** Conceptualization, Methodology, Investigation, Validation, Funding acquisition, Project administration, Resources, Supervision, Writing – review & editing.

## Appendix A Supplementary materials

Supplementary Data S1 Supplementary Raw Research Data. This is open data under the CC BY license http://creativecommons.org/licenses/by/4.0/ Supplementary Data S1
