# Supplementary material for: Identification of community structure-based brain states and transitions using functional MRI
Source: Neuroimage. 2021 Dec 1;244:118635. doi: 10.1016/j.neuroimage.2021.118635 (PMC8905300; doi:10.1016/j.neuroimage.2021.118635)
Supplement: Supplementary Data S1 — Supplementary Raw Research Data. This is open data under the CC BY license http://creativecommons.org/licenses/by/4.0/ [file mmc1.pdf]

Supplementary information:

Identification of community structure-based brain states and transitions using functional MRI

Lingbin Bian [a,b,*], Tiangang Cui [a], B.T. Thomas Yeo[c], Alex Fornito [b,d], Adeel Razi [b,d,e,f,+,*] and Jonathan Keith [a,+]

[a]School of Mathematics, Monash University, Australia
[b]Turner Institute for Brain and Mental Health, School of Psychological Sciences, Monash University, Australia
[c]Department of Electrical and Computer Engineering, National University of Singapore, Singapore
[d]Monash Biomedical Imaging, Monash University, Australia
[e]Wellcome Centre for Human Neuroimaging, University College London, United Kingdom
[f]CIFAR Azrieli Global Scholars Program, CIFAR, Toronto, Canada
[+]Joint senior authors
[*]Corresponding authors: Lingbin Bian (lingbin.bian@monash.edu) and Adeel Razi (adeel.razi@monash.edu)

1 Bayesian modelling for functional connectivity

1.1 Clustering with latent block model

Mathematically, we denote the community memberships (also called the latent labels) of the nodes as a vector $\mathbf{z} = (z_1, \ldots, z_N)$ such that $z_i \in \{1, \cdots, K\}$ denotes the community containing node i. Each z_i independently follows the categorical (one-trial multinomial) distribution:

$$z_i \sim \text{ Categorical}(1; \mathbf{r} = \{r_1, \cdots, r_K\}), \tag{1.1}$$

where r_k is the probability of a node being assigned to community k and $\sum_{k=1}^{K} r_k = 1$. The categorical probability can be expressed using the indicator function $I_k(z_i)$ as

$$p(z_i|\mathbf{r}, K) = \prod_{k=1}^{K} r_k^{I_k(z_i)}, \text{where } I_k(z_i) = \begin{cases} 1, & \text{if } z_i = k \\ 0, & \text{if } z_i \neq k \end{cases} . \tag{1.2}$$

This implies that the N dimensional vector $\mathbf{z}$ is generated with probability

$$p(\mathbf{z}|\mathbf{r}, K) = \prod_{k=1}^{K} r_k^{m_k(\mathbf{z})}, \tag{1.3}$$

where $m_k(\mathbf{z}) = \sum_{i=1}^{N} I_k(z_i)$. The latent allocation parameter vector $\mathbf{r} = (r_1, \cdots, r_K)$ is assumed to have a K-dimensional Dirichlet prior with density

$$p(\mathbf{r}|K) = N(\boldsymbol{\alpha}) \prod_{k=1}^{K} r_k^{\alpha_k - 1}, \tag{1.4}$$

where the normalization factor is $N(\boldsymbol{\alpha}) = \frac{\Gamma(\sum_{k=1}^{K} \alpha_k)}{\prod_{k=1}^{K} \Gamma(\alpha_k)}$. In this work we suppose $\alpha_k = 1$ for $k = 1, \ldots, K$, so that the prior for $\mathbf{r}$ is uniform on the K-simplex. Edges between nodes are represented using an adjacency matrix $\mathbf{x} \in \Re^{N \times N}$. We define a block $\mathbf{x}_{kl}$ comprised of weighted edges connecting the nodes in community k to the nodes in community l. The likelihood of the latent block model can be expressed as

$$p(\mathbf{x}|\boldsymbol{\pi}, \mathbf{z}, K) = \prod_{k.l} p(\mathbf{x}_{kl}|\pi_{kl}, \mathbf{z}, K), \tag{1.5}$$

and the likelihood in specific blocks can be expanded as

$$p(\mathbf{x}_{kl}|\pi_{kl}, \mathbf{z}, K) = \prod_{\{i|z_i=k\}} \prod_{\{j|z_j=l\}} p(x_{ij}|\pi_{kl}, \mathbf{z}, K), \tag{1.6}$$

where $\boldsymbol{\pi} = \{\pi_{kl}\}$ is a $K \times K$ model parameter matrix.

1.2 The latent block model with weighted edges

The block model parameter in block kl is $\pi_{kl} = (\mu_{kl}, \sigma_{kl}^2)$ and each x_{ij} in the block kl follows a Gaussian distribution conditional on $\mathbf{z}$ under the model K, that is

$$x_{ij}|\pi_{kl}, \mathbf{z}, K \sim \mathcal{N}(\mu_{kl}, \sigma_{kl}^2).$$

The parameter vectors $\pi_{kl} = (\mu_{kl}, \sigma_{kl}^2)$ are assumed to independently follow the conjugate Normal-Inverse-Gamma (NIG) prior $\pi_{kl} \sim \text{NIG}(\xi, \kappa^2\sigma_{kl}^2, \nu/2, \rho/2)$. That is, $\mu_{kl} \sim \mathcal{N}(\xi, \kappa^2\sigma_{kl}^2)$ and $\sigma_{kl}^2 \sim \text{IG}(\nu/2, \rho/2)$. The density of the Inverse-Gamma distribution $\text{IG}(\alpha, \beta)$ has the general formula $p(x) = \frac{\beta^\alpha}{\Gamma(\alpha)} x^{-(\alpha+1)} e^{(\frac{-\beta}{x})}$, where α and β are hyper-parameters.

We define $s_{kl}(\mathbf{x})$ to be the sum of the edge weights in the block kl and $q_{kl}(\mathbf{x})$ to be the sum of squares as follows:

$$s_{kl}(\mathbf{x}) = \sum_{i:z_i=k} \sum_{j:z_j=l} x_{ij}, \tag{1.7}$$

and

$$q_{kl}(\mathbf{x}) = \sum_{i:z_i=k}\sum_{j:z_j=l} x_{ij}^2. \quad (1.8)$$

We also define $w_{kl}(\mathbf{z}) = m_k(\mathbf{z})m_l(\mathbf{z})$ to be the number of elements in the block, where m_k and m_l are the numbers of nodes in community k and l respectively. The prior and the likelihood in the above expression is the NIG-Gaussian conjugate pair. With this conjugate pair, we can calculate the posterior distribution for each model block, which is also a Normal-Inverse-Gamma distribution $\mu_{kl} \sim \mathcal{N}(\xi_n, \kappa_n^2\sigma_{kl}^2)$ and $\sigma_{kl}^2 \sim \mathrm{IG}(\nu_n/2, \rho_n/2)$, where

$$\nu_n = \nu + w_{kl}, \quad (1.9)$$

$$\kappa_n^2 = \frac{\kappa^2}{1 + w_{kl}\kappa^2}, \quad (1.10)$$

$$\xi_n = \frac{\xi + s_{kl}\kappa^2}{1 + w_{kl}\kappa^2}, \quad (1.11)$$

$$\rho_n = \frac{\xi^2}{\kappa^2} + q_{kl} + \rho - \frac{(\xi + s_{kl}\kappa^2)^2}{1/\kappa^2 + w_{kl}}. \quad (1.12)$$

Details of the derivation of this $\mathrm{NIG}(\xi_n, \kappa_n^2\sigma_{kl}^2, \nu_n/2, \rho_n/2)$ distribution are provided in **SI Section** 2. The posterior density of the whole model is a product of such terms for all blocks, as follows.

$$p(\boldsymbol{\pi}|\mathbf{x}, \mathbf{z}) = \prod_{k,l} p(\pi_{kl}|\mathbf{x}_{kl}, \mathbf{z}). \quad (1.13)$$

Given a sampled $\mathbf{z}$ we can draw $\boldsymbol{\pi}$ from the above posterior directly. Methods for sampling the latent vector $\mathbf{z}$ will be discussed later in the paper.

1.3 The collapsed posterior of latent label vector

In this model, a change-point corresponds to a change in community architecture i.e., a change in the latent label vector $\mathbf{z}$ and the parameter matrix $\boldsymbol{\pi}$. For the sake of computational efficiency, it is convenient to construct the collapsed posterior distribution $p(\mathbf{z}|\mathbf{x}, K)$. We can obtain the collapsed posterior by integrating out the nuisance parameters (MacDaid et al., 2012, Wyse and Friel, 2012). In this section, we discuss the details of collapsing the latent block model when the edge weights are continuously valued.

Given K, the joint density of $\mathbf{x}$, $\boldsymbol{\pi}$, $\mathbf{z}$, and $\mathbf{r}$ is

$$p(\mathbf{x}, \boldsymbol{\pi}, \mathbf{z}, \mathbf{r}|K) = p(\mathbf{z}, \mathbf{r}|K)p(\mathbf{x}, \boldsymbol{\pi}|\mathbf{z}). \quad (1.14)$$

The parameters $\mathbf{r}$ and $\boldsymbol{\pi}$ can be integrated out (collapsed) to obtain the marginal density $p(\mathbf{x}, \mathbf{z}|K)$.

$$p(\mathbf{z}, \mathbf{x}|K) = \int p(\mathbf{z}, \mathbf{r}|K)d\mathbf{r} \int p(\mathbf{x}, \boldsymbol{\pi}|\mathbf{z})d\boldsymbol{\pi}, \quad (1.15)$$

so that the posterior for the block-wise model can be expressed as

$$p(\mathbf{z}|\mathbf{x}, K) \propto p(\mathbf{z}, \mathbf{x}|K) = \int p(\mathbf{z}, \mathbf{r}|K)d\mathbf{r} \prod_{k,l} \int p(\mathbf{x}_{kl}, \pi_{kl}|\mathbf{z})d\pi_{kl}. \quad (1.16)$$

The first integral $p(\mathbf{z}|K) = \int p(\mathbf{z}, \mathbf{r}|K)d\mathbf{r}$, where the integral is over the K-simplex, can be evaluated as follows:

$$\int p(\mathbf{z}, \mathbf{r}|K)d\mathbf{r} = \frac{\Gamma(\sum_{k=1}^K \alpha_k)}{\Gamma(\sum_{k=1}^K (\alpha_k + m_k(\mathbf{z}))} \quad (1.17)$$

$$\times \prod_{k=1}^K \frac{\Gamma(\alpha_k + m_k(\mathbf{z}))}{\Gamma(\alpha_k)}. \quad (1.18)$$

The details of this derivation are in **SI Section** 3 below. The integral of the form $\int p(\mathbf{x}_{kl}, \pi_{kl}|\mathbf{z})d\pi_{kl}$ can be evaluated as

$$\begin{aligned}\int p(\mathbf{x}_{kl}, \pi_{kl}|\mathbf{z})d\pi_{kl} &= \frac{\rho^{\nu/2}\Gamma\{(w_{kl}+\nu)/2\}}{\pi^{w_{kl}/2}\Gamma(\nu/2)(w_{kl}\kappa^2+1)^{1/2}} && (1.19)\\ &\quad\times(-\frac{\kappa^2(s_{kl}+\xi/\kappa^2)^2}{w_{kl}\kappa^2+1}+\frac{\xi^2}{\kappa^2} && (1.20)\\ &\quad+q_{kl}+\rho)^{-(w_{kl}+\nu)/2} && (1.21)\end{aligned}$$

The derivation is in **SI Section** 4.

1.4 Sampling from the collapsed posterior

We use a Markov chain Monte Carlo (MCMC) method to sample the latent label vector from the posterior with proposal moves $p(\mathbf{z} \to \mathbf{z}^*)$ similar to those of the allocation sampler (Nobile and Fearnside, 2007) to update $\mathbf{z}$. In the Metropolis-Hastings algorithm (Hastings, 1970), a candidate latent label vector $\mathbf{z}^*$ is accepted with probability $\min\{1, r\}$, where

$$r = \frac{p(K, \mathbf{z}^*, \mathbf{x})p(\mathbf{z}^* \to \mathbf{z})}{p(K, \mathbf{z}, \mathbf{x})p(\mathbf{z} \to \mathbf{z}^*)}. \quad (1.22)$$

In each iteration of the sampler, we perform either a Gibbs move or an M3 move, with equal probability (0.5) of each. Each Gibbs move updates the latent label vector $\mathbf{z}$ by drawing from the collapsed posterior $p(\mathbf{z}|\mathbf{x}, K)$. At each iteration, one entry z_i is randomly selected and updated by drawing from

$$p(z_i^*|z_{-i}, \mathbf{x}, K) = \frac{1}{C}p(z_1, \cdots, z_{i-1}, z_i^* = k, z_{i+1}, \cdots, z_n|\mathbf{x}), \quad (1.23)$$

where $k \in \{1, \cdots, K\}$, z_{-i} represents the elements in $\mathbf{z}$ apart from z_i and the normalization term

$$C = p(z_{-i}|\mathbf{x}, K) = \sum_{k=1}^{K} p(z_1, \cdots, z_{i-1}, z_i^* = k, z_{i+1}, \cdots, z_n|\mathbf{x}). \quad (1.24)$$

For a Gibbs move within a Metropolis-Hastings sampler, the ratio r always equals one. The computational complexity of a Gibbs move depends on the cost of calculating the probability of the reassignment of a specific entry. Each probability takes $O(K^2 + N^2)$ time to calculate. There are K possible reassignments so that each Gibbs move takes $O(K^3 + KN^2)$ time.

The details of the M3 move are provided in **SI Section** 5. The computational complexity of the M3 move depends on the cost of calculating the ratio of posterior density and proposal density. The time cost of calculating this ratio is $O(K^2 + N^2)$, and calculating the proposal ratio takes $O(N + L^2)$ time, so the M3 move takes $O(K^2 + N^2 + L^2)$ time.

2 The likelihood and posterior of the latent block model with weighted edges

Likelihood: The likelihood of the block kl with weighted edges is

$$\begin{aligned}
p(\mathbf{x}_{kl}|\pi_{kl},\mathbf{z},K) &= \prod_{\{i|z_i=k\}}\prod_{\{j|z_j=l\}} p(x_{ij}|\mu_{kl},\sigma^2_{kl},\mathbf{z},K) \\
&= (2\pi\sigma^2_{kl})^{-w_{kl}/2}\exp\{-\frac{1}{2\sigma^2_{kl}}\sum_{i:z_i=k}\sum_{j:z_j=l}(x_{ij}-\mu_{kl})^2\} \\
&= (2\pi\sigma^2_{kl})^{-w_{kl}/2} \\
&\quad \times\exp\{-\frac{1}{2\sigma^2_{kl}}(\sum_{i:z_i=k}\sum_{j:z_j=l}x^2_{ij}-2\sum_{i:z_i=k}\sum_{j:z_j=l}x_{ij}\mu_{kl} \\
&\quad +\sum_{i:z_i=k}\sum_{j:z_j=l}\mu^2_{kl})\} \\
&= (2\pi\sigma^2_{kl})^{-w_{kl}/2}\exp\{-\frac{1}{2\sigma^2_{kl}}(q_{kl}-2\mu_{kl}s_{kl}+w_{kl}\mu^2_{kl})\}, \qquad (2.1)
\end{aligned}$$

where w_{kl} is the number of elements in block kl, s_{kl} is the sum of the weights and q_{kl} is the sum of squares of the weights in the block kl.

Posterior: We derive the posterior of the model parameter π_{kl} with prior $\mu_{kl}\sim\mathcal{N}(\xi,\kappa^2\sigma^2_{kl})$ and $\sigma^2_{kl}\sim \text{IG}(\nu/2,\rho/2)$ as follows.

$$\begin{aligned}
p(\pi_{kl}|\mathbf{x}_{kl},\mathbf{z},K) &\propto p(\pi_{kl})p(\mathbf{x}_{kl}|\pi_{kl},\mathbf{z},K) \\
&= p(\mu_{kl})p(\sigma^2_{kl})\prod_{\{i|z_i=k\}}\prod_{\{j|z_j=l\}} p(x_{ij}|\mu_{kl},\sigma^2_{kl},\mathbf{z},K) \\
&= (2\pi\kappa^2\sigma^2_{kl})^{-1/2}\exp\{-\frac{1}{2\kappa^2\sigma^2_{kl}}(\mu_{kl}-\xi)^2\} \\
&\quad \times\frac{(\rho/2)^{\nu/2}}{\Gamma(\nu/2)}\sigma_{kl}^{-2(\nu/2+1)}\exp\{-\rho/2\sigma^2_{kl}\} \\
&\quad \times(2\pi\sigma^2_{kl})^{-w_{kl}/2}\exp\{-\frac{1}{2\sigma^2_{kl}}(q_{kl}-2\mu_{kl}s_{kl}+w_{kl}\mu^2_{kl})\} \\
&= \frac{(\rho/2)^{\nu/2}}{\Gamma(\nu/2)}(2\pi\kappa^2)^{-1/2}(2\pi)^{-w_{kl}/2}\sigma^{-1}_{kl}\sigma_{kl}^{-\nu-2-w_{kl}} \\
&\quad \times\exp\{-\frac{1}{2\sigma^2_{kl}}[(\frac{1}{\kappa^2}+w_{kl})\mu^2_{kl}-2(\frac{1}{\kappa^2}\xi+s_{kl})\mu_{kl} \\
&\quad +\frac{1}{\kappa^2}\xi^2+q_{kl}+\rho]\} \qquad (2.2)
\end{aligned}$$

The posterior of the Gaussian model is also a Normal-Inverse-Gamma distribution which can be denoted as $\mu_{kl}\sim\mathcal{N}(\xi_n,\kappa^2_n\sigma^2_{kl})$ and $\sigma^2_{kl}\sim\text{IG}(\nu_n/2,\rho_n/2)$. The posterior density can be expressed as

$$\begin{aligned}
p(\pi_{kl}|\mathbf{x}_{kl},\mathbf{z},K) &= (2\pi\kappa^2_n\sigma^2_{kl})^{-1/2}\exp\{-\frac{1}{2\kappa^2_n\sigma^2_{kl}}(\mu_{kl}-\xi_n)^2\} \\
&\quad \times\frac{(\rho_n/2)^{\nu_n/2}}{\Gamma(\nu_n/2)}\sigma_{kl}^{-2(\nu_n/2+1)}\exp\{-\rho_n/2\sigma^2_{kl}\} \\
&= \frac{(\rho_n/2)^{\nu_n/2}}{\Gamma(\nu_n/2)}(2\pi\kappa^2_n)^{-1/2}\sigma^{-1}_{kl}\sigma_{kl}^{-\nu_n-2} \\
&\quad \times\exp\{-\frac{1}{2\sigma^2_{kl}}(\frac{1}{\kappa^2_n}\mu^2_{kl}-\frac{2\xi_n}{\kappa^2_n}\mu_{kl}+\frac{\xi^2_n}{\kappa^2_n}+\rho_n)\}. \qquad (2.3)
\end{aligned}$$

Comparing the terms and coefficients with respect to μ_{kl}^2, μ_{kl} and σ_{kl}^2,

$$-\nu_n - 2 = -\nu - 2 - w_{kl}, \tag{2.4}$$

$$\frac{1}{\kappa_n^2} = \frac{1}{\kappa^2} + w_{kl}, \tag{2.5}$$

$$\frac{2\xi_n}{\kappa_n^2} = 2(\frac{1}{\kappa^2}\xi + s_{kl}), \tag{2.6}$$

$$\frac{\xi_n^2}{\kappa_n^2} + \rho_n = \frac{1}{\kappa^2}\xi^2 + q_{kl} + \rho. \tag{2.7}$$

In summary, the parameters of the posterior density are given by

$$\nu_n = \nu + w_{kl}, \tag{2.8}$$

$$\kappa_n^2 = \frac{\kappa^2}{1 + w_{kl}\kappa^2}, \tag{2.9}$$

$$\xi_n = \frac{\xi + s_{kl}\kappa^2}{1 + w_{kl}\kappa^2}, \tag{2.10}$$

$$\rho_n = \frac{\xi^2}{\kappa^2} + q_{kl} + \rho - \frac{(\xi + s_{kl}\kappa^2)^2}{1/\kappa^2 + w_{kl}}. \tag{2.11}$$

We can directly sample π_{kl} from $\mathrm{NIG}(\xi_n, \kappa_n^2\sigma_{kl}^2, \nu_n/2, \rho_n/2)$.

3 Collapse r in latent block model

We show the calculation of $p(\mathbf{z}|K) = \int p(\mathbf{z}, \mathbf{r}|K)d\mathbf{r}$. Given the K-dimensional Dirichlet prior with density $p(\mathbf{r}|K) = N(\boldsymbol{\alpha})\prod_{k=1}^K r_k^{\alpha_k - 1}$, where $\boldsymbol{\alpha} = \{\alpha_1, \cdots, \alpha_K\}$, $N(\boldsymbol{\alpha}) = \frac{\Gamma(\sum_{k=1}^K \alpha_k)}{\prod_{k=1}^K \Gamma(\alpha_k)}$; and the likelihood $p(\mathbf{z}|\mathbf{r}, K) = \prod_{k=1}^K r_k^{m_k(\mathbf{z})}$, we can collapse $\mathbf{r}$ as follows:

$$\begin{aligned}
p(\mathbf{z}|K) &= \int p(\mathbf{z}, \mathbf{r}|K)d\mathbf{r} \\
&= \int p(\mathbf{r}|K)p(\mathbf{z}|\mathbf{r}, K)d\mathbf{r} \\
&= \int \frac{\Gamma(\sum_{k=1}^K \alpha_k)}{\prod_{k=1}^K \Gamma(\alpha_k)} \prod_{k=1}^K r_k^{\alpha_k - 1} \prod_{k=1}^K r_k^{m_k} d\mathbf{r} \\
&= \frac{\Gamma(\sum_{k=1}^K \alpha_k)}{\prod_{k=1}^K \Gamma(\alpha_k)} \frac{\prod_{k=1}^K \Gamma(\alpha_k + m_k)}{\Gamma(\sum_{k=1}^K(\alpha_k + m_k))} \\
&\quad \times \int \frac{\Gamma(\sum_{k=1}^K(\alpha_k + m_k))}{\prod_{k=1}^K \Gamma(\alpha_k + m_k)} \prod_{k=1}^K r_k^{\alpha_k + m_k - 1} d\mathbf{r} \\
&= \frac{\Gamma(\sum_{k=1}^K \alpha_k)}{\Gamma(\sum_{k=1}^K(\alpha_k + m_k))} \prod_{k=1}^K \frac{\Gamma(\alpha_k + m_k)}{\Gamma(\alpha_k)} \qquad (3.1)
\end{aligned}$$

4 Collapse π_{kl} in latent block model with weighted edges

The collapsed posterior of the latent block model is described in the work by (Wyse and Friel, 2012), but the details of the collapsing procedure are not described there. We elaborate the collapsing procedure of the Gaussian latent block model. We collapse μ_{kl} and σ^2_{kl} respectively to get the integral.

$$\begin{aligned}\int p(\mathbf{x}_{kl}, \pi_{kl}|\mathbf{z})d\pi_{kl} &= \int\int p(\mathbf{x}_{kl}, \mu_{kl}, \sigma^2_{kl}|\mathbf{z})d\mu_{kl}d\sigma^2_{kl} \\ &= \int\int p(\mu_{kl})p(\sigma^2_{kl})p(\mathbf{x}_{kl}|\mu_{kl}, \sigma^2_{kl}, \mathbf{z})d\mu_{kl}d\sigma^2_{kl} \qquad (4.1)\end{aligned}$$

To facilitate integrating with respect to μ_{kl}, we denote

$$I_{\mu_{kl}} = \int p(\mathbf{x}_{kl}, \mu_{kl}, \sigma^2_{kl}|\mathbf{z})d\mu_{kl}, \qquad (4.2)$$

then

$$\begin{aligned}I_{\mu_{kl}} &= \frac{(\rho/2)^{\nu/2}}{\Gamma(\nu/2)}(2\pi\kappa^2)^{-1/2}(2\pi)^{-w_{kl}/2}\sigma^{-1}_{kl}\sigma^{-\nu-2-w_{kl}}_{kl} \\ &\times\int \exp\{-\frac{1}{2\sigma^2_{kl}}[(\frac{1}{\kappa^2}+w_{kl})\mu^2_{kl} - 2(\frac{1}{\kappa^2}\xi + s_{kl})\mu_{kl} \\ &+\frac{1}{\kappa^2}\xi^2 + q_{kl} + \rho]\}du_{kl}. \qquad (4.3)\end{aligned}$$

Let

$$M = \frac{(\rho/2)^{\nu/2}}{\Gamma(\nu/2)}(2\pi\kappa^2)^{-1/2}(2\pi)^{-w_{kl}/2}\sigma^{-1}_{kl}\sigma^{-\nu-2-w_{kl}}_{kl}, \qquad (4.4)$$

so that

$$I_{\mu_{kl}} = M \times \int \exp\{-\frac{1}{2\sigma^2_{kl}}[\lambda(\mu_{kl}-m)^2 - \lambda m^2 + \frac{1}{\kappa^2}\xi^2 + q_{kl} + \rho]\}du_{kl}, \qquad (4.5)$$

where

$$\lambda = \frac{1}{\kappa^2} + w_{kl}, \qquad (4.6)$$

and

$$m = \frac{\frac{1}{\kappa^2}\xi + s_{kl}}{\frac{1}{\kappa^2} + w_{kl}}. \qquad (4.7)$$

Then

$$\begin{aligned}I_{\mu_{kl}} &= M \times (2\pi\frac{\sigma^2_{kl}}{\lambda})^{1/2}\int(2\pi\frac{\sigma^2_{kl}}{\lambda})^{-1/2}\exp\{-\frac{1}{2\sigma^2_{kl}}\lambda(\mu_{kl}-m)^2\} \\ &\times\exp\{-\frac{1}{2\sigma^2_{kl}}(-\lambda m^2 + \frac{1}{\kappa^2}\xi^2 + q_{kl} + \rho)\}du_{kl} \\ &= M \times (2\pi\frac{\sigma^2_{kl}}{\lambda})^{1/2} \times \exp\{-\frac{1}{2\sigma^2_{kl}}(-\lambda m^2 + \frac{1}{\kappa^2}\xi^2 + q_{kl} + \rho)\} \\ &= (2\pi)^{-w_{kl}/2}\frac{(\rho/2)^{\nu/2}}{\Gamma(\nu/2)}\sigma^{-\nu-w_{kl}-2}_{kl}(w_{kl}\kappa^2+1)^{-1/2} \\ &\times\exp\{-\frac{1}{2\sigma^2_{kl}}[-\frac{(\frac{1}{\kappa^2}\xi + s_{kl})^2}{\frac{1}{\kappa^2}+w_{kl}} + \frac{1}{\kappa^2}\xi^2 + q_{kl} + \rho]\}. \qquad (4.8)\end{aligned}$$

To facilitate integration with respect to σ^2_{kl}, we first rewrite $I_{\mu_{kl}}$ as follows

$$I_{\mu_{kl}} = (2\pi)^{-w_{kl}/2}\frac{(\rho/2)^{\nu/2}}{\Gamma(\nu/2)}(w_{kl}\kappa^2+1)^{-1/2}\frac{\Gamma(\alpha)}{\beta^\alpha}\frac{\beta^\alpha}{\Gamma(\alpha)}(\sigma^2_{kl})^{-(\alpha+1)}e^{(\frac{-\beta}{\sigma^2_{kl}})}, \qquad (4.9)$$

where

$$\alpha = \frac{1}{2}\nu + \frac{1}{2}w_{kl}, \tag{4.10}$$

and

$$\beta = \frac{1}{2}[-\frac{(\frac{1}{\kappa^2}\xi + s_{kl})^2}{\frac{1}{\kappa^2} + w_{kl}} + \frac{1}{\kappa^2}\xi^2 + q_{kl} + \rho]. \tag{4.11}$$

This can be integrated as follows

$$\begin{aligned}
\int I_{\mu_{kl}} d\sigma^2_{kl} &= (2\pi)^{-w_{kl}/2}\frac{(\rho/2)^{\nu/2}}{\Gamma(\nu/2)}(w_{kl}\kappa^2+1)^{-1/2}\frac{\Gamma(\alpha)}{\beta^\alpha} \\
&= (2\pi)^{-w_{kl}/2}\frac{(\rho/2)^{\nu/2}}{\Gamma(\nu/2)}(w_{kl}\kappa^2+1)^{-1/2} \\
&\quad \times \frac{\Gamma(\frac{1}{2}\nu + \frac{1}{2}w_{kl})}{(\frac{1}{2}[-\frac{(\frac{1}{\kappa^2}\xi+s_{kl})^2}{\frac{1}{\kappa^2}+w_{kl}} + \frac{1}{\kappa^2}\xi^2 + q_{kl} + \rho])^{(\frac{1}{2}\nu+\frac{1}{2}w_{kl})}} \\
&= \frac{\rho^{\nu/2}\Gamma\{(w_{kl}+\nu)/2\}}{\pi^{w_{kl}/2}\Gamma(\nu/2)(w_{kl}\kappa^2+1)^{1/2}} \\
&\quad \times(-\frac{\kappa^2(s_{kl}+\xi/\kappa^2)^2}{w_{kl}\kappa^2+1} + \frac{\xi^2}{\kappa^2} + q_{kl} + \rho)^{-(w_{kl}+\nu)/2}.
\end{aligned} \tag{4.12}$$

In summary,

$$\begin{aligned}
\int p(\mathbf{x}_{kl}, \pi_{kl}|\mathbf{z}) d\pi_{kl} &= \int\int p(\mathbf{x}_{kl}, \mu_{kl}, \sigma^2_{kl}|\mathbf{z}) d\mu_{kl} d\sigma^2_{kl} \\
&= \frac{\rho^{\nu/2}\Gamma\{(w_{kl}+\nu)/2\}}{\pi^{w_{kl}/2}\Gamma(\nu/2)(w_{kl}\kappa^2+1)^{1/2}} \\
&\quad \times(-\frac{\kappa^2(s_{kl}+\xi/\kappa^2)^2}{w_{kl}\kappa^2+1} + \frac{\xi^2}{\kappa^2} + q_{kl} + \rho)^{-(w_{kl}+\nu)/2}.
\end{aligned} \tag{4.13}$$

5 The M3 move

In a Gibbs move, only one entry in $\mathbf{z}$ is updated at each iteration. An alternative is the M3 move (Nobile and Fearnside, 2007), which updates multiple entries of $\mathbf{z}$ simultaneously. In M3, two communities in $\mathbf{z}$ are randomly selected and denoted as k_1 and k_2. Each element z_i in the selected communities is reassigned to k_1 or k_2 with probability $P^i_{k_1}$ and $P^i_{k_2}$ respectively, to form the updated $\mathbf{z}^*$. The collection of elements of $\mathbf{z}$ with labels k_1 or k_2 may be indexed by the set $I = \{i : z_i = k_1 \text{ or } z_i = k_2\}$. Let the number of such elements be L. The remaining elements of $\mathbf{z}$ are collected into a subvector denoted as $\widetilde{\mathbf{z}}$. For the update, one element z_i with $i \in I$ is randomly selected and updated to z^*_i according to a reassignment probability. The updated element is added to $\widetilde{\mathbf{z}}$. The size of I thus becomes $L - 1$. This procedure is repeated until all the elements of I are processed (the length of I becomes 0) and the resulting vector $\widetilde{\mathbf{z}}$ becomes the proposed move $\mathbf{z}^*$. We define a sub-adjacency matrix $\widetilde{\mathbf{x}}$ as the observations corresponding to $\widetilde{\mathbf{z}}$ and the observations $\mathbf{x}^{*i}$ corresponding to the updated z^*_i. The probabilities of the reassignment satisfy $P^i_{k_1} + P^i_{k_2} = 1$ and the ratio

$$\begin{aligned}
\frac{P^i_{k_1}}{P^i_{k_2}} &= \frac{p(z^*_i = k_1|\widetilde{\mathbf{z}}, \widetilde{\mathbf{x}}, \mathbf{x}^{*i}, K)}{p(z^*_i = k_2|\widetilde{\mathbf{z}}, \widetilde{\mathbf{x}}, \mathbf{x}^{*i}, K)} \\
&= \frac{p(z^*_i = k_1, \widetilde{\mathbf{z}}, \widetilde{\mathbf{x}}, \mathbf{x}^{*i}|K)}{p(z^*_i = k_2, \widetilde{\mathbf{z}}, \widetilde{\mathbf{x}}, \mathbf{x}^{*i}|K)} \\
&= \frac{p(z^*_i = k_1, \widetilde{\mathbf{z}}|K)}{p(z^*_i = k_2, \widetilde{\mathbf{z}}|K)}\frac{p(\widetilde{\mathbf{x}}, \mathbf{x}^{*i}|z^*_i = k_1, \widetilde{\mathbf{z}}, K)}{p(\widetilde{\mathbf{x}}, \mathbf{x}^{*i}|z^*_i = k_2, \widetilde{\mathbf{z}}, K)}.
\end{aligned} \tag{5.1}$$

The first term of this ratio is given by

$$\begin{aligned}\frac{p(z_i^* = k_1, \widetilde{\mathbf{z}}|K)}{p(z_i^* = k_2, \widetilde{\mathbf{z}}|K)} &= \frac{\Gamma(\alpha_{k_1} + \widetilde{m}_{k_1}(\widetilde{\mathbf{z}}) + 1)}{\Gamma(\alpha_{k_1} + \widetilde{m}_{k_1}(\widetilde{\mathbf{z}}))} \frac{\Gamma(\alpha_{k_2} + \widetilde{m}_{k_2}(\widetilde{\mathbf{z}}))}{\Gamma(\alpha_{k_2} + \widetilde{m}_{k_2}(\widetilde{\mathbf{z}}) + 1)} \\ &= \frac{\alpha_{k_1} + \widetilde{m}_{k_1}(\widetilde{\mathbf{z}})}{\alpha_{k_2} + \widetilde{m}_{k_2}(\widetilde{\mathbf{z}})}, \end{aligned} \tag{5.2}$$

where $\widetilde{m}_{k_1}(\widetilde{\mathbf{z}})$ and $\widetilde{m}_{k_2}(\widetilde{\mathbf{z}})$ are the numbers of nodes in community k_1 and k_2 in $\widetilde{\mathbf{z}}$. The second term of the ratio is given by

$$\begin{aligned}\frac{p(\widetilde{\mathbf{x}}, \mathbf{x}^{*i}|z_i^* = k_1, \widetilde{\mathbf{z}}, K)}{p(\widetilde{\mathbf{x}}, \mathbf{x}^{*i}|z_i^* = k_2, \widetilde{\mathbf{z}}, K)} &= \frac{p(\mathbf{x}^{*i}|\widetilde{\mathbf{x}}, z_i^* = k_1, \widetilde{\mathbf{z}}, K)}{p(\mathbf{x}^{*i}|\widetilde{\mathbf{x}}, z_i^* = k_2, \widetilde{\mathbf{z}}, K)} \\ &= \frac{p(\mathbf{x}^{*i}|\widetilde{\mathbf{x}}^{k_1}, z_i^* = k_1, \widetilde{\mathbf{z}}, K)}{p(\mathbf{x}^{*i}|\widetilde{\mathbf{x}}^{k_2}, z_i^* = k_2, \widetilde{\mathbf{z}}, K)} \\ &= \frac{p(\widetilde{\mathbf{x}}^{k_1}, \mathbf{x}^{*i}|z_i^* = k_1, \widetilde{\mathbf{z}})}{p(\widetilde{\mathbf{x}}^{k_1}|z_i^* = k_1, \widetilde{\mathbf{z}})} \frac{p(\widetilde{\mathbf{x}}^{k_2}|z_i^* = k_2, \widetilde{\mathbf{z}})}{p(\widetilde{\mathbf{x}}^{k_2}, \mathbf{x}^{*i}|z_i^* = k_1, \widetilde{\mathbf{z}})}. \end{aligned} \tag{5.3}$$

Finally, the reassignment probability is given by

$$\frac{P_{k_1}^i}{1 - P_{k_1}^i} = \frac{\alpha_{k_1} + \widetilde{m}_{k_1}(\widetilde{\mathbf{z}})}{\alpha_{k_2} + \widetilde{m}_{k_2}(\widetilde{\mathbf{z}})} \frac{p(\widetilde{\mathbf{x}}^{k_1}, \mathbf{x}^{*i}|z_i^* = k_1, \widetilde{\mathbf{z}})}{p(\widetilde{\mathbf{x}}^{k_1}|z_i^* = k_1, \widetilde{\mathbf{z}})} \frac{p(\widetilde{\mathbf{x}}^{k_2}|z_i^* = k_2, \widetilde{\mathbf{z}})}{p(\widetilde{\mathbf{x}}^{k_2}, \mathbf{x}^{*i}|z_i^* = k_1, \widetilde{\mathbf{z}})}. \tag{5.4}$$

and the proposal ratio is given by

$$\frac{p(\mathbf{z}^* \to \mathbf{z})}{p(\mathbf{z} \to \mathbf{z}^*)} = \prod_{i \in I} \frac{P_{z_i}^i}{P_{z_i^*}^i}. \tag{5.5}$$

6 Summary of the algorithms

6.1 Bayesian change-point detection by posterior predictive discrepancy

Algorithm 1 Bayesian change-point detection by posterior predictive discrepancy

Input: Time series $\mathbf{Y}$ of one subject, length of time course T, window size W, number of communities K.

For $t = \frac{W}{2} + 1, \cdots, T - \frac{W}{2}$
    Calculate $\mathbf{Y}_t \to \mathbf{x}_t$ where $\mathbf{x}_t$ is the correlation matrix.
    Draw samples $\{\mathbf{z}^i, \boldsymbol{\pi}^i\}$ $(i = 1, \cdots, S)$ from the posterior $P(\mathbf{z}, \boldsymbol{\pi} | \mathbf{x}, K)$.
    Simulate replicated adjacency matrix $\mathbf{x}^{rep^i}$ from the predictive distribution $P(\mathbf{x}^{rep} | \mathbf{z}, \boldsymbol{\pi}, K)$.
    Calculate the disagreement index $\gamma(\mathbf{x}^{rep^i}; \mathbf{x})$.
    Calculate the posterior predictive discrepancy index $\overline{\gamma}_t = \frac{\sum_{i=1}^{S} \gamma(\mathbf{x}^{rep^i}; \mathbf{x})}{S}$.
End
For $t = \frac{W}{2} + \frac{W_s}{2} + 1, \cdots, T - \frac{W}{2} - \frac{W_s}{2}$
    Calculate cumulative discrepancy energy $E(t) = \sum_{I=t-\frac{W_s}{2}}^{t+\frac{W_s}{2}-1} \overline{\gamma}_I$.
End

6.2 Removing false positives for local extrema in CDE

Algorithm 2 Removing false positives for local extrema in CDE

Input: Group-averaged CDE time series $E(t)$.

Calculate the time points of local extrema in $E(t)$: $\{t_1, t_2, \cdots, t_m, \cdots, t_M\}$, where M is the number of local extrema.
Create empty storage $C = \{c_{ij}\}$, a threshold of time distance τ.
Initiation of indicator indices $i = 1$, $j = 1$.
For $m = 1, \cdots, M - 1$
    If $|t_m - t_{m+1}| < \tau$,
        $c_{ij} = t_m$.
        $i = i + 1$.
    Else if $|t_m - t_{m+1}| \geq \tau$,
        $c_{ij} = t_m$.
        $j = j + 1$.
        $i = 1$.
    End
End
$c_{ij} = t_M$.
Create empty storage $C' = \{c'_l\}$.
For $l = 2 : j - 1$
    If $\min\{E(c_{1l}), \cdots, E(c_{il})\} > \max\{E(c_{1(l-1)}), \cdots, E(c_{i(l-1)}), E(c_{1(l+1)}), \cdots, E(c_{i(l+1)})\}$,
        $c'_l = \arg\max_c \{E(c_{1l}), \cdots, E(c_{il})\}$.
    Else if $\max\{E(c_{1l}), \cdots, E(c_{il})\} < \min\{E(c_{1(l-1)}), \cdots, E(c_{i(l-1)}), E(c_{1(l+1)}), \cdots, E(c_{i(l+1)})\}$,
        $c'_l = \arg\min_c \{E(c_{1l}), \cdots, E(c_{il})\}$.
    Else
        Remove c'_l.
    End
End
Output: C', the estimated locations of change-points and discrete states after removing false positives.

7 Label switching

For the latent block model, we set $\alpha_k = 1$ with $\{k = 1, \cdots, K\}$, and constant values of ξ, κ^2, ν and ρ for all of the blocks kl, so the prior is symmetric with respect to permutations of community labels. Permutations of community labels do not change the likelihood, which means the distributions with respect to blocks are not identifiable. Therefore, the posterior is also invariant to permutations of community labels. In the Markov chain, the labels of the latent label vector switch occasionally: this effect is known as the label switching phenomenon (Stephens, 2000, Nobile and Fearnside, 2007, Wyse and Friel, 2012). For global fitting, label switching does not affect the results of posterior predictive discrepancy. However, for local inference, we need to assign the labels to the communities unequivocally to estimate the memberships of the nodes.

We define a distance indicating the difference of coordinates between two latent label vectors $\mathbf{z}$ and $\mathbf{z}'$,

$$D(\mathbf{z}, \mathbf{z}') = \sum_{i=1}^{N} I(z_i \neq z_i'), \tag{7.1}$$

where I is the indicator function. We define

$$\boldsymbol{\sigma} = \{\sigma(1), \cdots, \sigma(k), \cdots, \sigma(K)\} \tag{7.2}$$

as a permutation of a labelling $\{1, \cdots, k, \cdots, K\}$. Let $\mathbf{Q} = \{\mathbf{z}^j(\boldsymbol{\sigma}^j), j = 1, \cdots, J\}$ be a collection of latent label vectors with respect to a sequence of permutations $\{\boldsymbol{\sigma}^j, j = 1, \cdots, J\}$. We want to minimize the sum of all distances between the vectors

$$\sum_{j=1}^{J-1} \sum_{l=j+1}^{J} D(\mathbf{z}^j(\boldsymbol{\sigma}^j), \mathbf{z}^l(\boldsymbol{\sigma}^l)). \tag{7.3}$$

The solution of this minimization can be considered as a sequential optimization problem of the square assignment. For each vector $\mathbf{z}^j$, if the vectors that have already been processed (relabelled) up to $j-1$ are $\{\mathbf{z}^t, t = 1, \cdots, j-1\}$, we define the element of a cost matrix

$$C(k_1, k_2) = \sum_{t=1}^{j-1} \sum_{i=1}^{N} D(z_i^t \neq k_1, z_i^j = k_2). \tag{7.4}$$

We use the square assignment algorithm (Carpaneto and Toth, 1980) returning a permutation $\boldsymbol{\sigma}^j$ which minimizes the total cost $\sum_{k=1}^{K} C(k, \sigma(k))$ for each $\mathbf{z}^j$. Finally, we permute the labels in the vector $\mathbf{z}^j$ according to $\boldsymbol{\sigma}^j$.

8 Generative model, synthetic data and parameter settings

To validate our Bayesian change-point detection algorithm, we use the multivariate Gaussian generative model to simulate the synthetic data. Specifically, we generate D segments of Gaussian time series from D different network architectures. The synthetic data contains the ground truth of $D-1$ change-points over the time course. The positions of the true change-points are denoted as a row vector $\mathbf{p} = [p_1, \cdots, p_{D-1}]$. Within each of D segments, we suppose nodes are assigned to K^{true} communities, the value of which differs in different segments. The true number of communities in the segments can be denoted as a vector $\mathbf{K}^{true} = [K_1^{true}, \cdots, K_D^{true}]$. We generate three types of dataset: data with various SNRs, data with various degree of inter-individual variations of community structures, and data with HRF.

8.1 Simulations with varying SNR

This set of simulations is used to evaluate the effect of SNR on the performance of the proposed Bayesian change-point detection method. For generating this synthetic data, we set the label vectors

that determine the form of the covariance matrices in the generative model to be $\{\mathbf{z}_1, \mathbf{z}_2, \cdots, \mathbf{z}_D\}$. A same collection of label vectors is used for each virtual subject such that there are no inter-individual variations of community structures between subjects at a same data segment. These label vectors are generated using the Dirichlet-Categorical conjugate pair. The component weights $\{\mathbf{r}_1, \mathbf{r}_2, \cdots, \mathbf{r}_D\}$ are first drawn from a uniform distribution on the $\mathbf{K}^{true}$ simplex and then nodes are assigned to the communities by drawing from the corresponding Categorical distributions. Time series data in $\Re^N$ are then simulated from

$$Y = f(\mathbf{z}, a, b) + \boldsymbol{\epsilon} \tag{8.1}$$

for $t = 1, \cdots, T$ by drawing $f(\mathbf{z}, a, b) \sim \mathcal{N}(\mathbf{0}, \mathbf{\Sigma}(\mathbf{z}, a, b))$, with

$$\Sigma_{ij} = \begin{cases} 1, & \text{if } i = j \\ a, & \text{if } i \neq j \text{ and } z_i = z_j \\ b, & \text{if } i \neq j \text{ and } z_i \neq z_j \end{cases} \tag{8.2}$$

where $a \sim U(0.8, 1)$ and $b \sim U(0, 0.2)$ are uniformly distributed, and $\boldsymbol{\epsilon} \sim \mathcal{N}(\mathbf{0}, \sigma^2\mathbf{I})$ is the additive Gaussian noise. A same sample of $\{a, b\}$ is used in the generative model for simulating the dataset of each subject. The resulting covariance matrices for D segments are denoted as $\{\mathbf{\Sigma}_1, \mathbf{\Sigma}_2, \cdots, \mathbf{\Sigma}_D\}$. For each virtual subject, the simulated data $\mathbf{Y} \in \Re^{N \times T}$ can be separated into D segments which are $\{\mathbf{Y}_1, \mathbf{Y}_2, \cdots, \mathbf{Y}_D\}$.

8.2 Simulations with varying DIIV

This set of simulations is used to evaluate the performance of Bayesian change-point detection for capturing the inter-individual variations of community structures. The generative models have different settings of $\{\mathbf{z}_1, \mathbf{z}_2, \cdots, \mathbf{z}_D\}$ for different subjects. Samples of $\{a, b\}$ are drawn independently from the uniform distribution for simulating the datasets of different subjects. The variation of the community structure is determined by a parameter called degree of inter-individual variations (DIIV). The DIIV is defined as the number of nodes that have different label assignments between subjects in a group. If DIIV $= n$, there are n nodes having different label assignments. In this case, n nodes are randomly selected from N nodes and each selected node is reassigned a label randomly drawn from the collection $\{1, \cdots, K\}$ for each subject. Therefore, the set of simulations in the previous section, with no inter-individual variations, is a special case for this set of experiments when DIIV $= 0$.

8.3 Simulations with HRF

This set of simulations is performed by convolving the multivariate Gaussian data with a canonical haemodynamic response function (HRF) as has been implement in SPM (using spm_hrf.m function). The parameters of HRF are set as follows. To imitate the real working memory task fMRI data, the scan repeat time of HRF is set to be 0.72 s; the delay of response (relative to onset) is 6 s; the delay of undershoot (relative to onset) is 16 s; the dispersion of response is 1 s; the dispersion of undershoot is 1 s; the ratio of response to undershoot is 6 s; the onset is at 0 s; and the length of kernel is 32 s.

8.4 Parameter settings

For validation, we first generate 100 instances (as virtual subjects) of synthetic multivariate time series for a network with $N = 35$ nodes and $T = 180$ time points to imitate the scenario of real data. We set the true change-points at $\{20, 50, 80, 100, 130, 160\}$ and the numbers of communities in the segments to be $\{3, 4, 5, 3, 5, 4, 3\}$. Here we define the signal-to-noise ratio (SNR) as $\frac{\Sigma_{ii}}{\sigma^2}$, and set different values of σ to control SNR ($\sigma = 0.3162$ for SNR = 10 dB, $\sigma = 0.5623$ for SNR = 5 dB, $\sigma = 1$ for SNR = 0 dB, and $\sigma = 1.7783$ for SNR = -5 dB). For simulations with various SNRs, we set SNR = 10 dB, 5 dB, 0 dB, and -5 dB respectively and DIIV = 0. For simulations with various DIIV, we set DIIV = 0, 5, and 10 respectively and SNR = 5 dB. For simulations with HRF, we apply HRF to the multivariate Gaussian data with SNR = 5 dB, and DIIV = 0, 5, and 10 respectively.

For global fitting, the posterior prediction replication number is set as $S = 50$ for all of our experiments. For local inference, we draw $S_s = 200$ samples from the posterior densities for both latent label vectors and model parameters. We set the prior to be $\mathrm{NIG}(\xi, \kappa^2\sigma_{kl}^2, \nu/2, \rho/2)$ with $\xi = 0$, $\kappa^2 = 1$, $\nu = 3$ and $\rho = 0.02$, which is non-informative.

SI Table 1:

2-back		0-back		Fixation	
Community	Node number	Community	Node number	Community	Node number
k=1		k=1	18	k=1	
k=2	11 30 31 32	k=2	11 31 32	k=2	11 30 31 32
k=3		k=3	16 20	k=3	12 16 20 21
k=4	1 9 17 34	k=4	9 17 34	k=4	1 7
k=5	2 23 24	k=5	23 24	k=5	3 24
k=6	8	k=6	5 10 26	k=6	5

SI Table 1. A table of community detection with session 2 (RL). This table summarises the nodes commonly located in a specific community k for all of picture types in the working memory tasks.

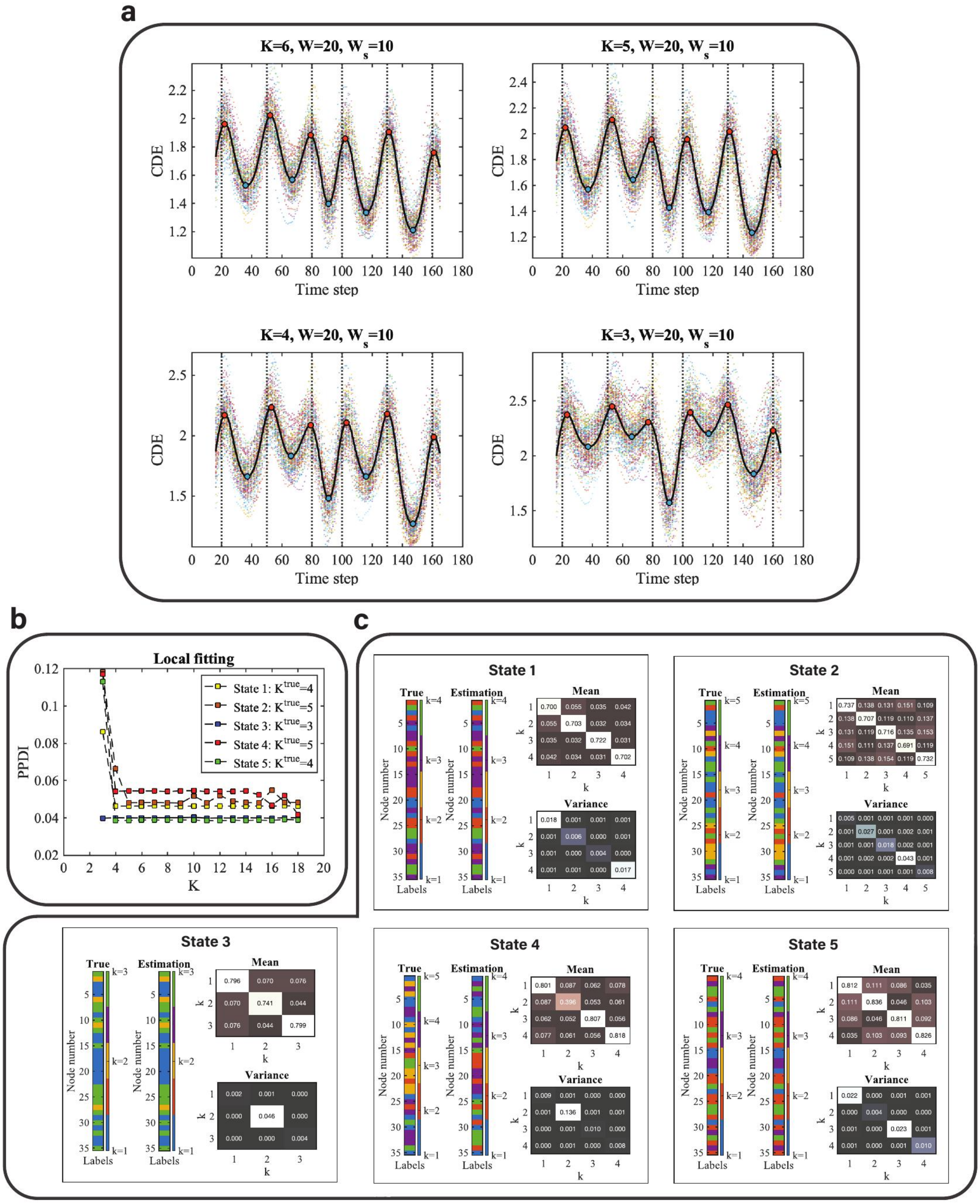

SI Figure 1. **Results of method validation using synthetic data with SNR = 10 dB.** **a** CDE of the multivariate Gaussian data with SNR = 10 dB using different models (K = 6, 5, 4, and 3). The sliding window size for converting from time series to correlation matrices sequence is W = 20, whereas (for smoothing) the sliding window size for converting from PPDI to CDE is W_s = 10. The vertical dashed lines are the locations of the true change-points (t = 20, 50, 80, 100, 130, and 160). The colored scatterplots in the figures are the CDEs of individual (virtual) subjects and the black curve is the group CDE (averaged CDE over 100 subjects). The red dots are the local maxima and the blue dots are the local minima. **b** Local fitting with different models (from K = 3 to 18) for synthetic data (SNR = 10 dB). Different colors represent the PPDI values of different states with the true number of communities K^{true}. **c** The estimation of community constituents for SNR = 10 dB at each discrete state: t = 36, 67, 91, 116, 147 for brain states 1 to 5, respectively. The estimations of the latent label vectors (**Estimation**) and the label vectors (**True**) that determine the covariance matrix in the generative model are shown as bar graphs. The strength and variation of the connectivity within and between communities are represented by the block mean and variance matrices within each panel.

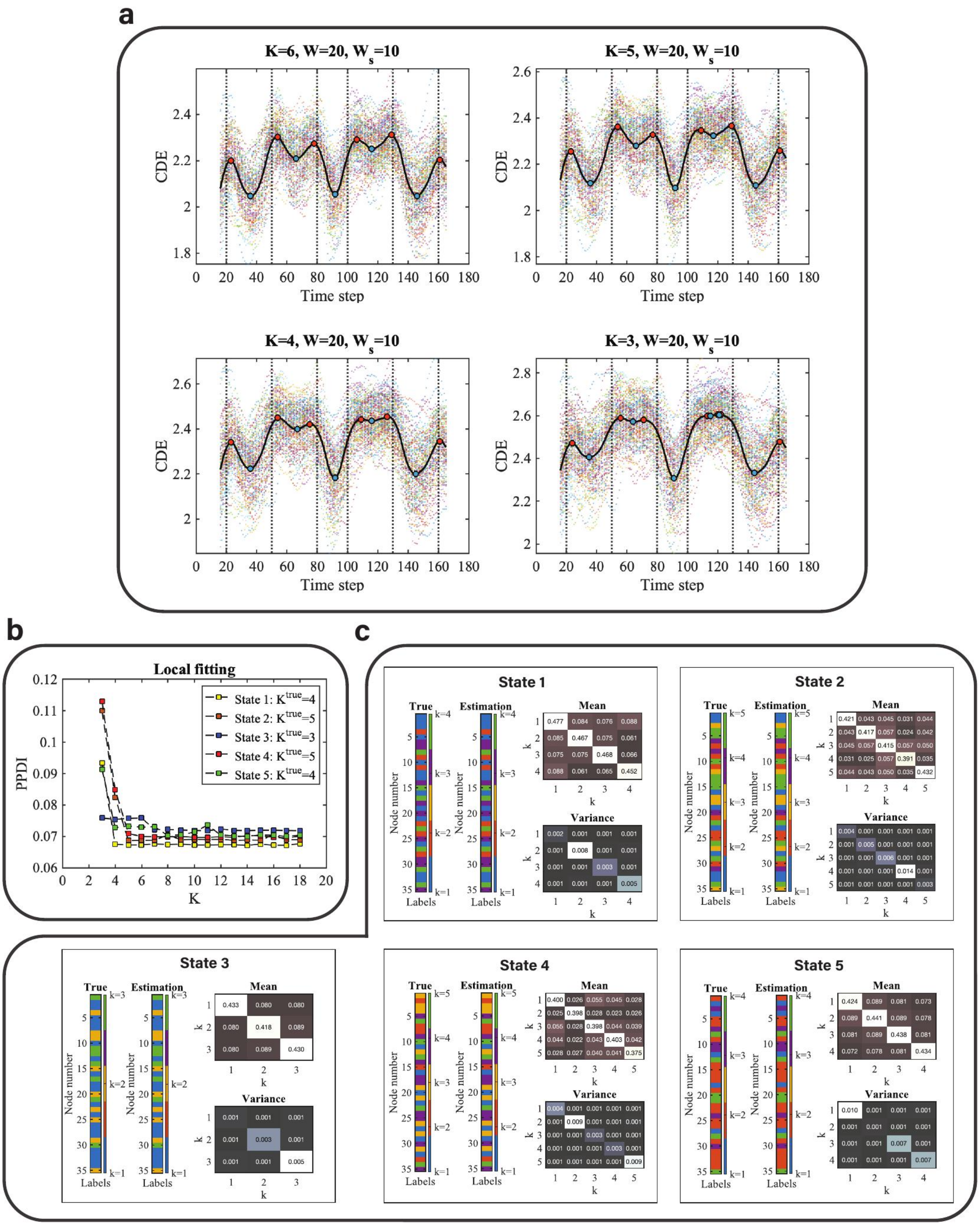

SI Figure 2. **Results of method validation using synthetic data with SNR $= 0$ dB.** **a** This figure is in the same format as the **SI Figure** 1 above only that it is for SNR $= 0$ dB. **b** Local fitting with different models (from $K = 3$ to 18) for synthetic data (SNR $= 0$ dB). **c** The estimation of community constituents for SNR $= 0$ dB at each discrete state: $t = 36$, 66, 92, 116, 146 for brain states 1 to 5, respectively.

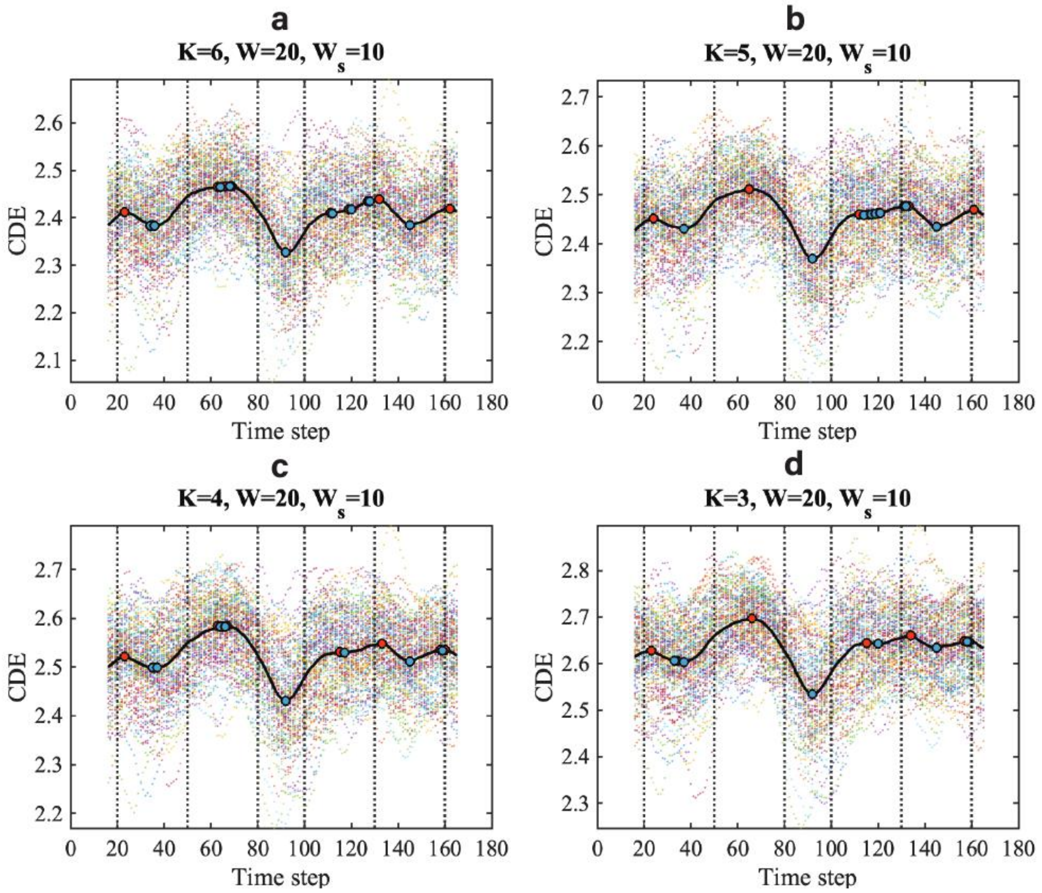

SI Figure 3. CDE of the multivariate Gaussian data with SNR = -5 dB. Different models (K = 6, 5, 4, and 3 in **a** to **d**) were used for global fitting. Change-point detection did not work in this case, hence the brain states can not be identified here.

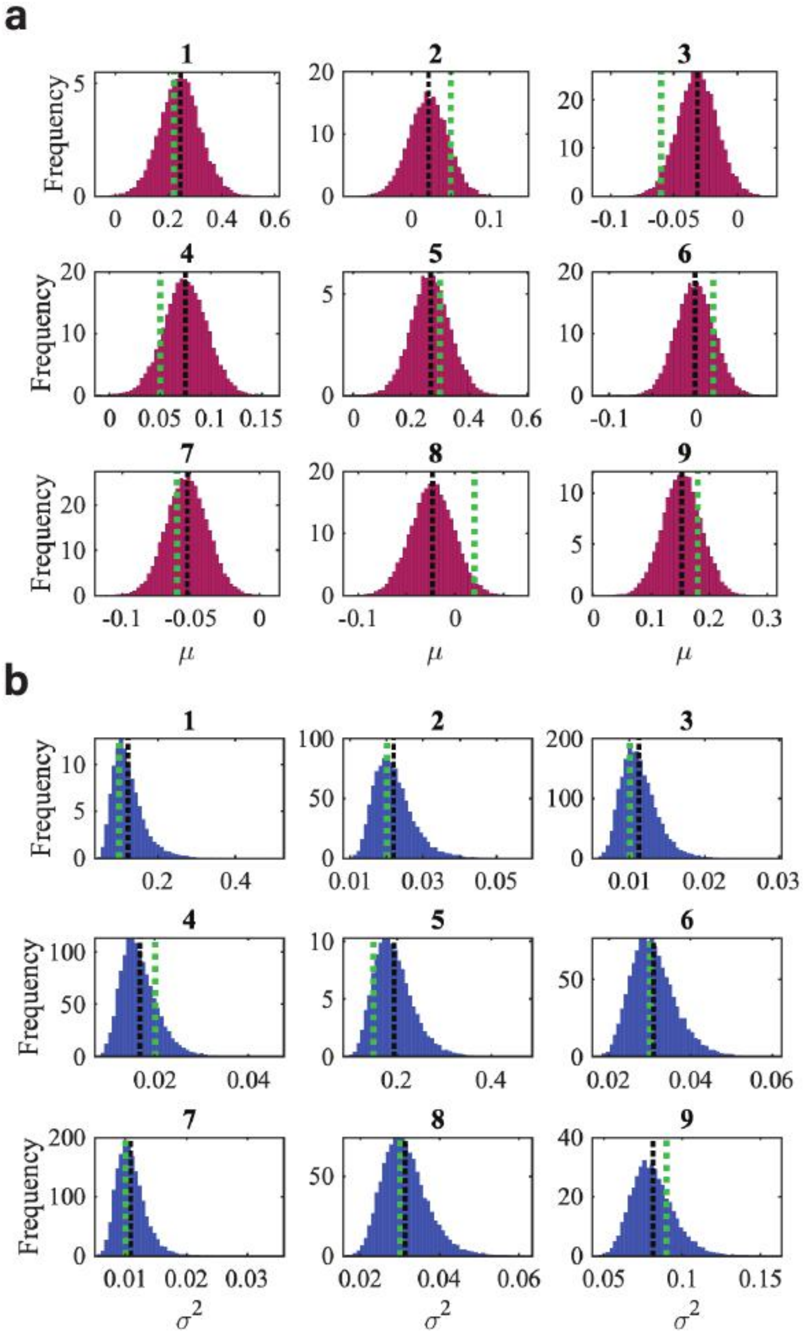

SI Figure 4. Validation of sampling the model parameters. **a** The histograms of the sampled block mean, and **b** the histograms of the sampled block variance for the case $K = 3$. We denote the block kl sequentially (for example, the block for $k = 2, l = 3$ is denoted as block 6; the block for $k = 3, l = 3$ is denoted as block 9). The green dashed lines are the true values and the black dashed lines are the estimates. In order to validate the algorithm for sampling the model parameters, we simulate a synthetic adjacency matrix from a mixture of Gaussian distributions with ground truth of $K = 3$, the true latent label vector (3, 2, 1, 1, 2, 3, 3, 3, 2, 2, 1, 3, 1, 2, 2, 2, 1, 3, 3, 3, 3), the true block mean matrix (0.22, 0.05, -0.06; 0.05, 0.30, 0.02; -0.06, 0.02, 0.18) and the true block variance matrix (0.1, 0.02, 0.01; 0.002, 0.15, 0.03; 0.01, 0.03, 0.09). Given this generated adjacency matrix as an observation, we draw samples of the block mean and variance from the posterior $p(\boldsymbol{\pi}|\mathbf{x}, \mathbf{z})$ conditional on $\mathbf{z}$. The shape of the histogram of mean is consistent with a Normal distribution and the histogram of variance is consistent with an Inverse-Gamma distribution. The figure shows that the estimations of the block mean and variance closely match the ground truth values.

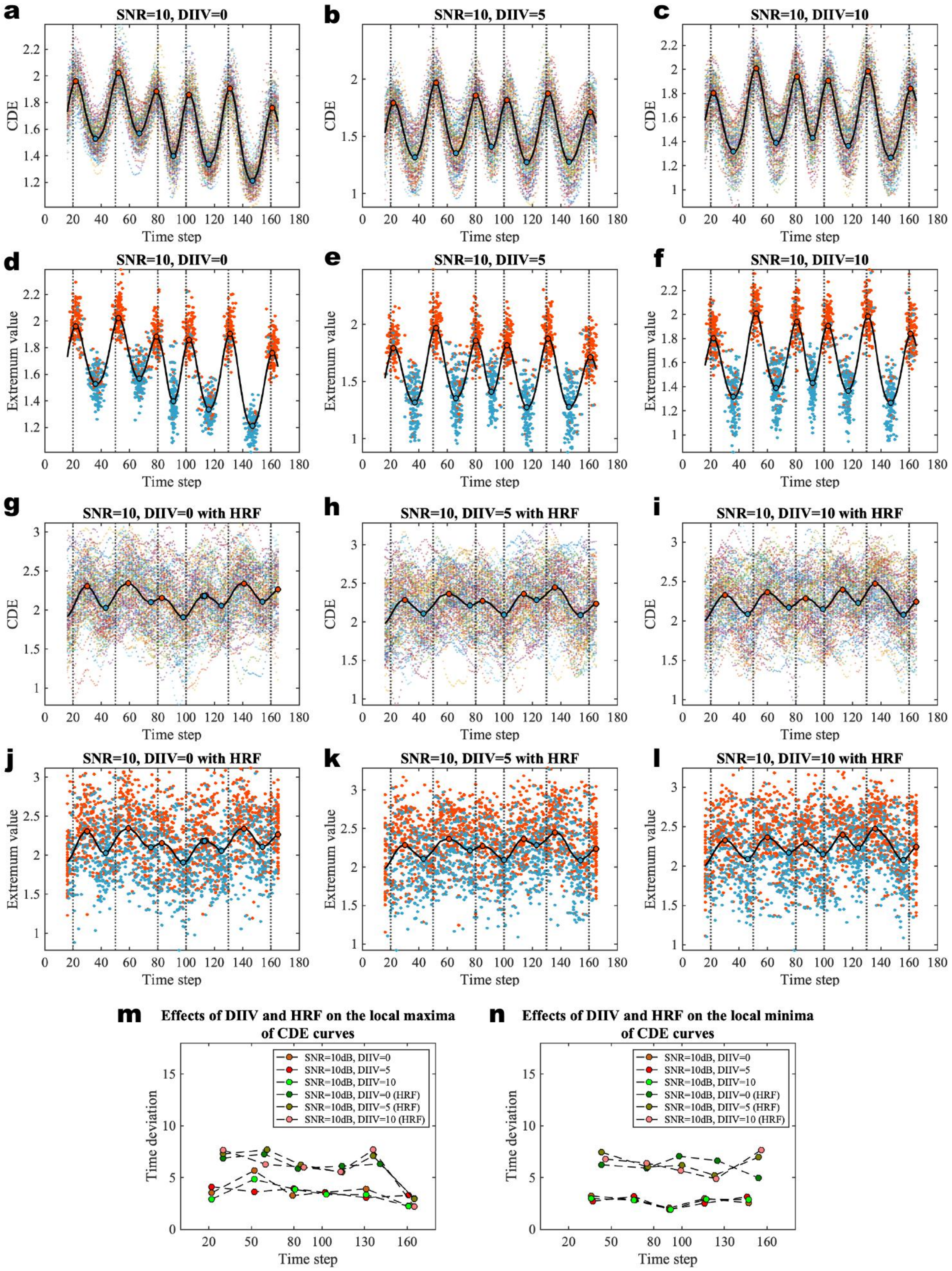

SI Figure 5. **Effects of DIIV and HRF on the inter-individual variations of CDE curves for SNR = 10 dB.** **a-c** CDE of the multivariate Gaussian data with DIIV = 0, 5, and 10 respectively. The number of communities $K = 6$ for all of the experiments. **d-f** The extrema of the individual-level CDE curves with different levels of DIIV. The red dots are the local maxima and the blue dots are the local minima of 100 virtual subjects. **g-i** CDE curves of the multivariate Gaussian data applied with haemodynamic response function (HRF). **j-l** The extrema of the individual-level CDE curves with HRF. **m** The time deviation of local maxima of individual-level CDE curves compared to the local maximum of the group-averaged CDE curve with different levels of DIIV and HRF. **n** The time deviation of local minima of individual-level CDE curves compared to the local minimum of the group-averaged CDE curve with different levels of DIIV and HRF.

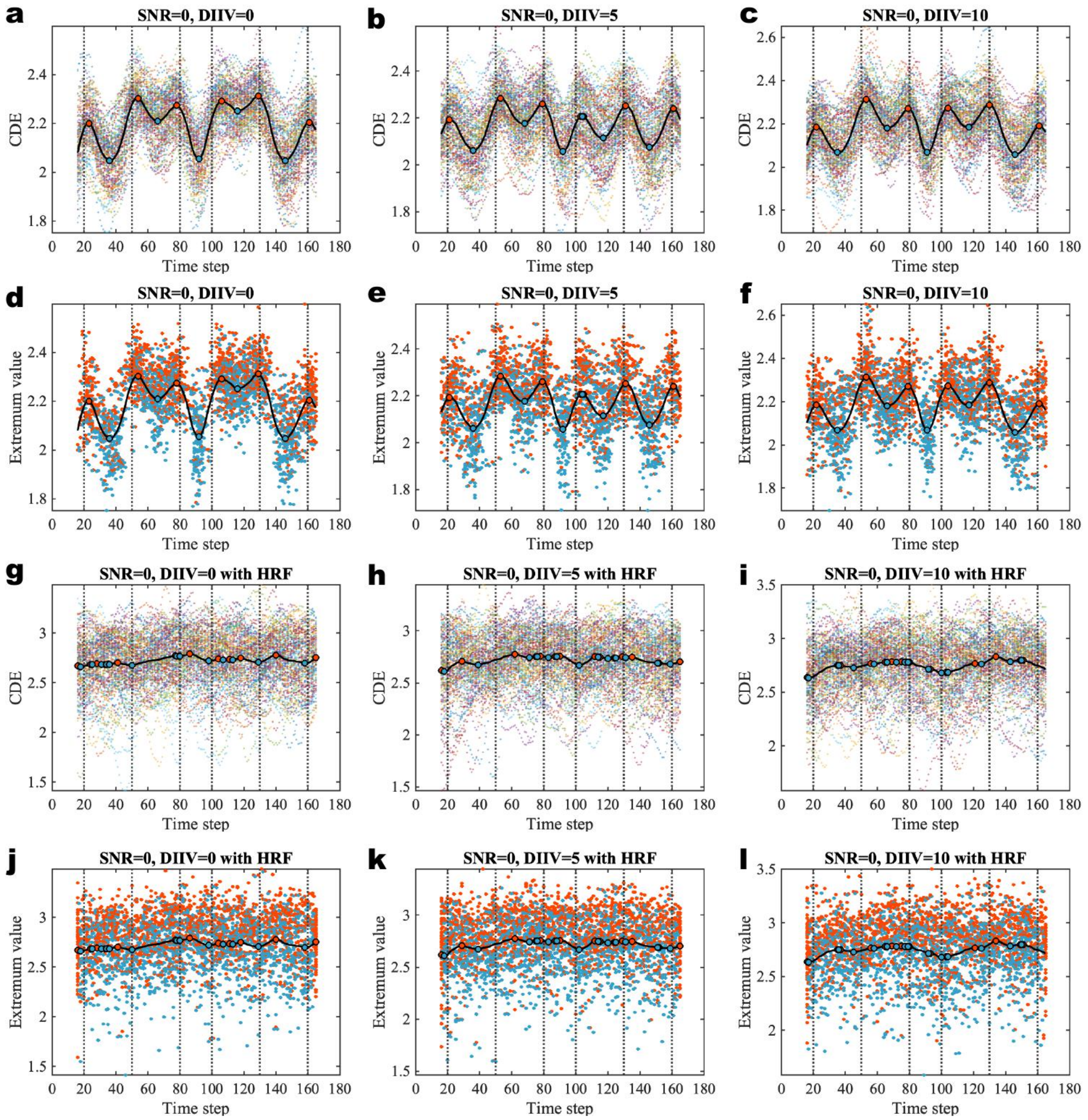

SI Figure 6. Effects of DIIV and HRF on the inter-individual variations of CDE curves for SNR = 0 dB. **a-c** CDE of the multivariate Gaussian data with DIIV = 0, 5, and 10 respectively. The number of communities $K = 6$ for all of the experiments. **d-f** The extrema of the individual-level CDE curves with different levels of DIIV. The red dots are the local maxima and the blue dots are the local minima of 100 virtual subjects. **g-i** CDE curves of the multivariate Gaussian data applied with haemodynamic response function (HRF). **j-l** The extrema of the individual-level CDE curves with HRF. We find the change-point detection fails for the dataset with SNR = 0 dB and with HRF.

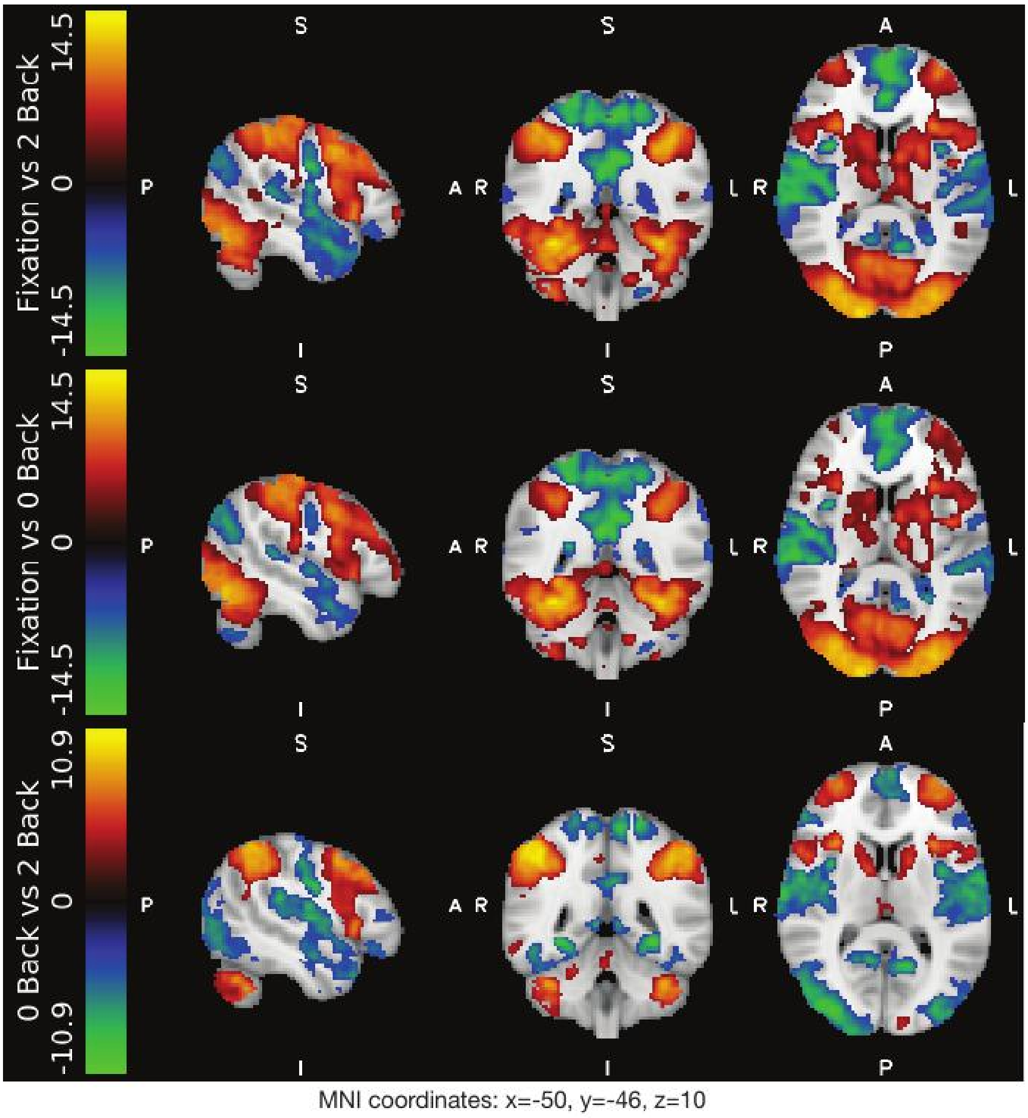

SI Figure 7. Task activation maps (thresholded Z-MAX maps) for group analysis. Contrasts of 2-back vs fixation, 0-back vs fixation and 2-back vs 0-back for MNI coordinates (x = -50, y = -46, z = 10). For running 1st-level GLM-based FEAT (Woolrich et al., 2001) in FSL, we added the confound predictors files released by HCP to the design matrix of the model for each individual. We then set up a 2nd-level design matrix for the contrast of 2-back, 0-back, and fixation. For the 3rd-level (the group-level GLM analysis (Woolrich et al., 2004)), we applied cluster-wise inference and set up the cluster defining threshold (CDT) to be $Z = 3.1$ ($P = 0.001$) to avoid cluster failure problems as described in (Eklund et al., 2016), with a family-wise error-corrected threshold of $P = 0.05$. Maps are viewed by looking upward from the feet of the subject and the coordinate directions are denoted as Anterior (A), Posterior (P), Superior (S), Inferior (I), Left (L), and Right (R).

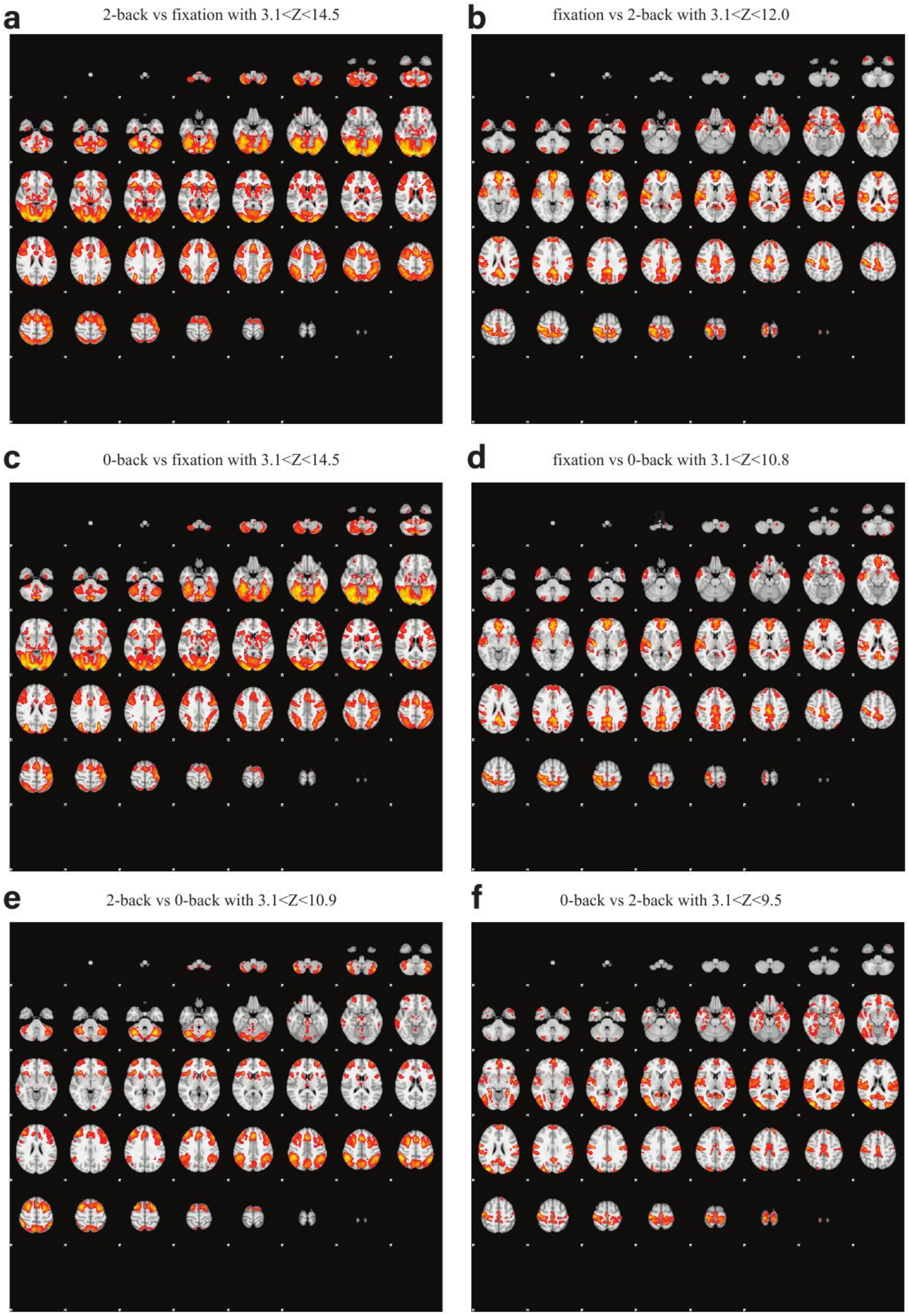

SI Figure 8. **The light box views of thresholded local maximum Z statistic with different contrasts.**

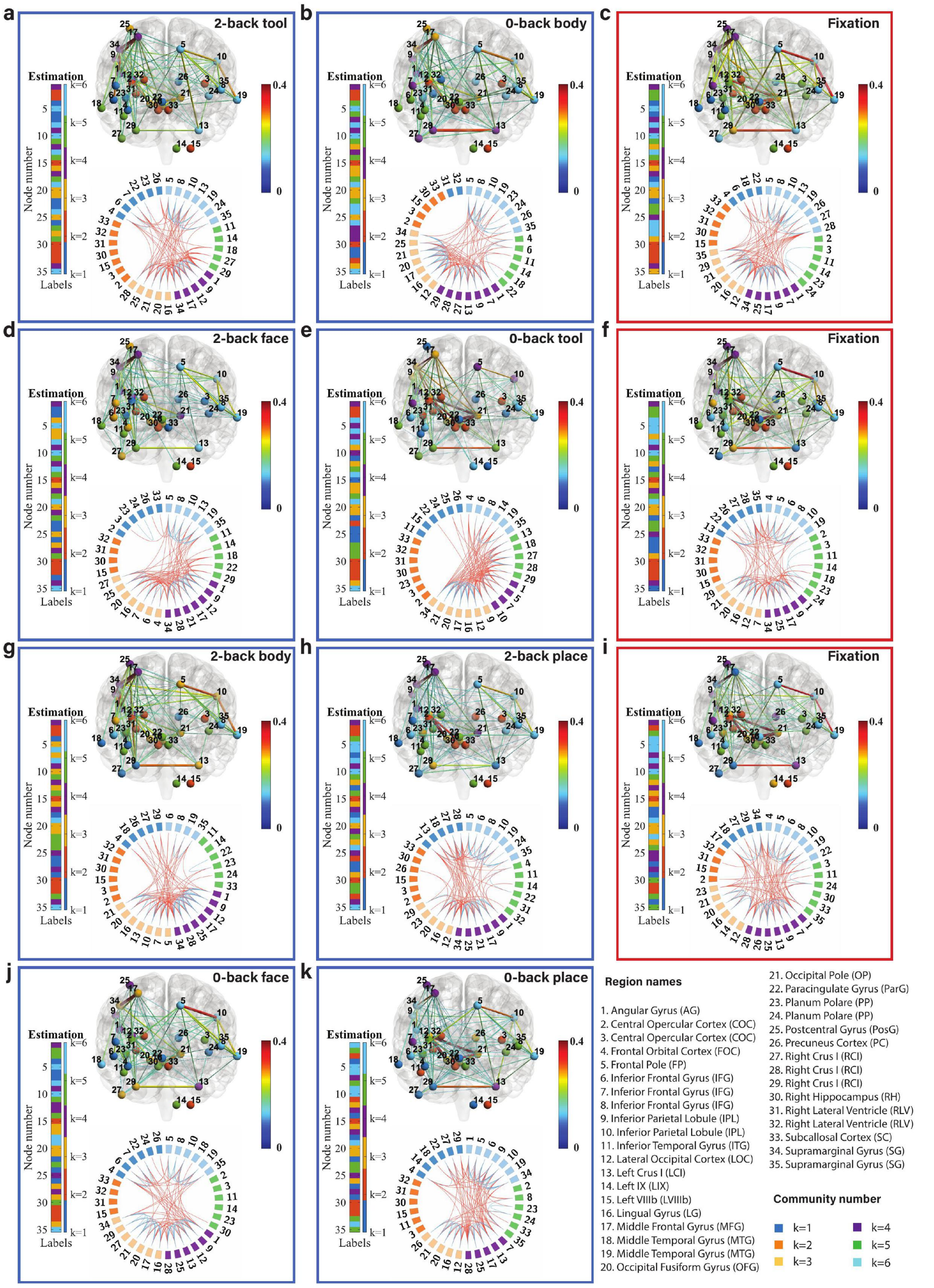

SI Figure 9. Community structure of the discrete brain states with sparsity level of 20% (session 1, LR). The figures with blue frames represent brain states corresponding to working memory tasks (2-back tool at $t = 41$; 0-back body at $t = 76$; 2-back face at $t = 140$; 0-back tool at $t = 175$; 2-back body at $t = 239$; 2-back place at $t = 278$; 0-back face at $t = 334$; and 0-back place at $t = 375$ in **a-k**) and those with red frames represent brain states corresponding to fixation (fixation at $t = 107, 206,$ and 306 in **c**, **f**, and **i**). Each brain state shows connectivity at a sparsity level of 20%. The different colors of the labels represent community memberships. The strength of the connectivity is represented by the colors shown in the bar at the right of each frame. In Circos maps, nodes in the same community are adjacent and have the same color. Node numbers and abbreviations of the corresponding brain regions are shown around the circles. In each frame, different colors represent different community numbers. The connectivity above the sparsity level is represented by arcs. The blue links represent connectivity within communities and the red links represent connectivity between communities.

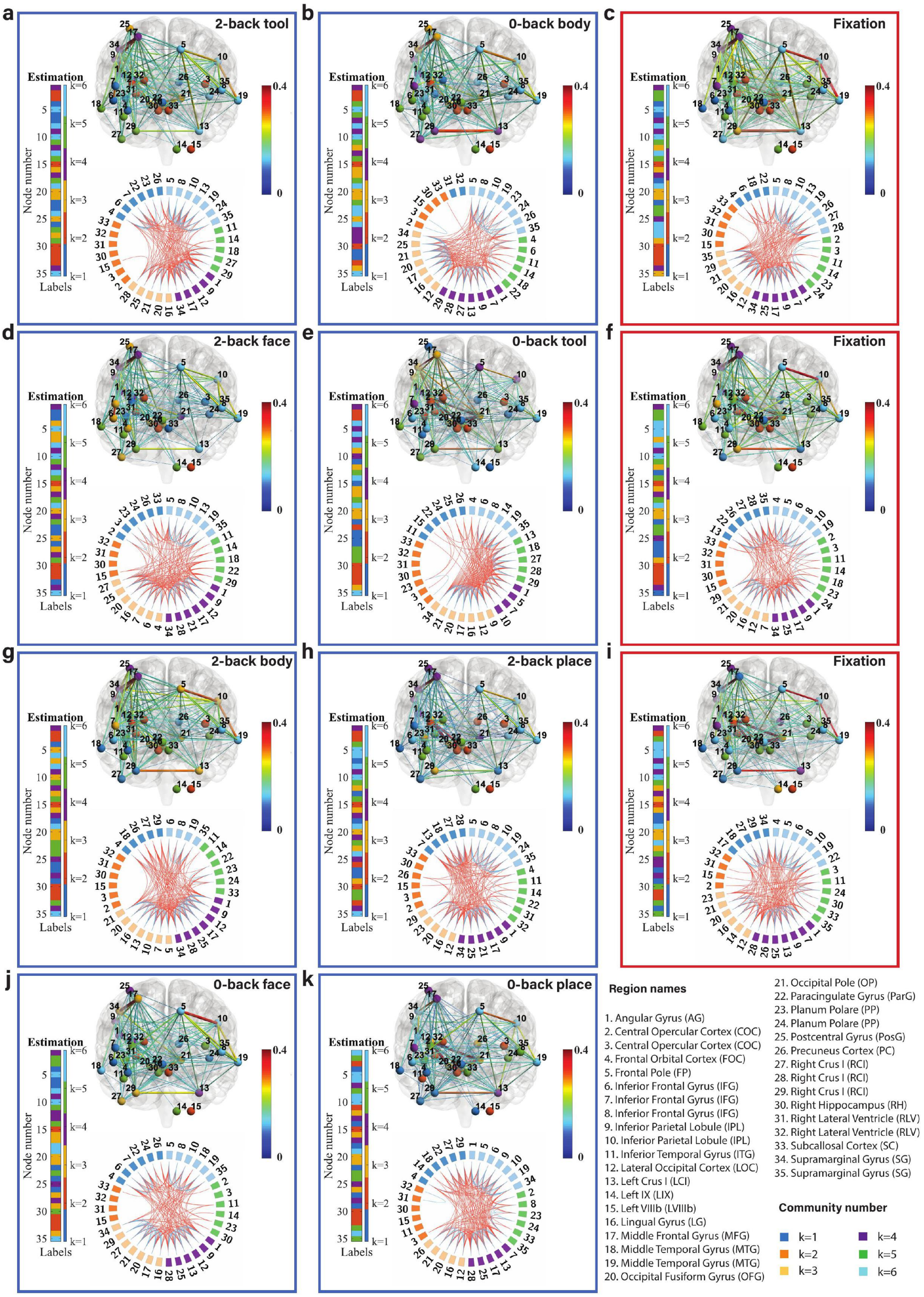

SI Figure 10. Community structure of the discrete brain states with sparsity level of 30% (session 1, LR). This figure is in the same format as the **SI Figure** 9 above only that it is for sparsity level of 30%.

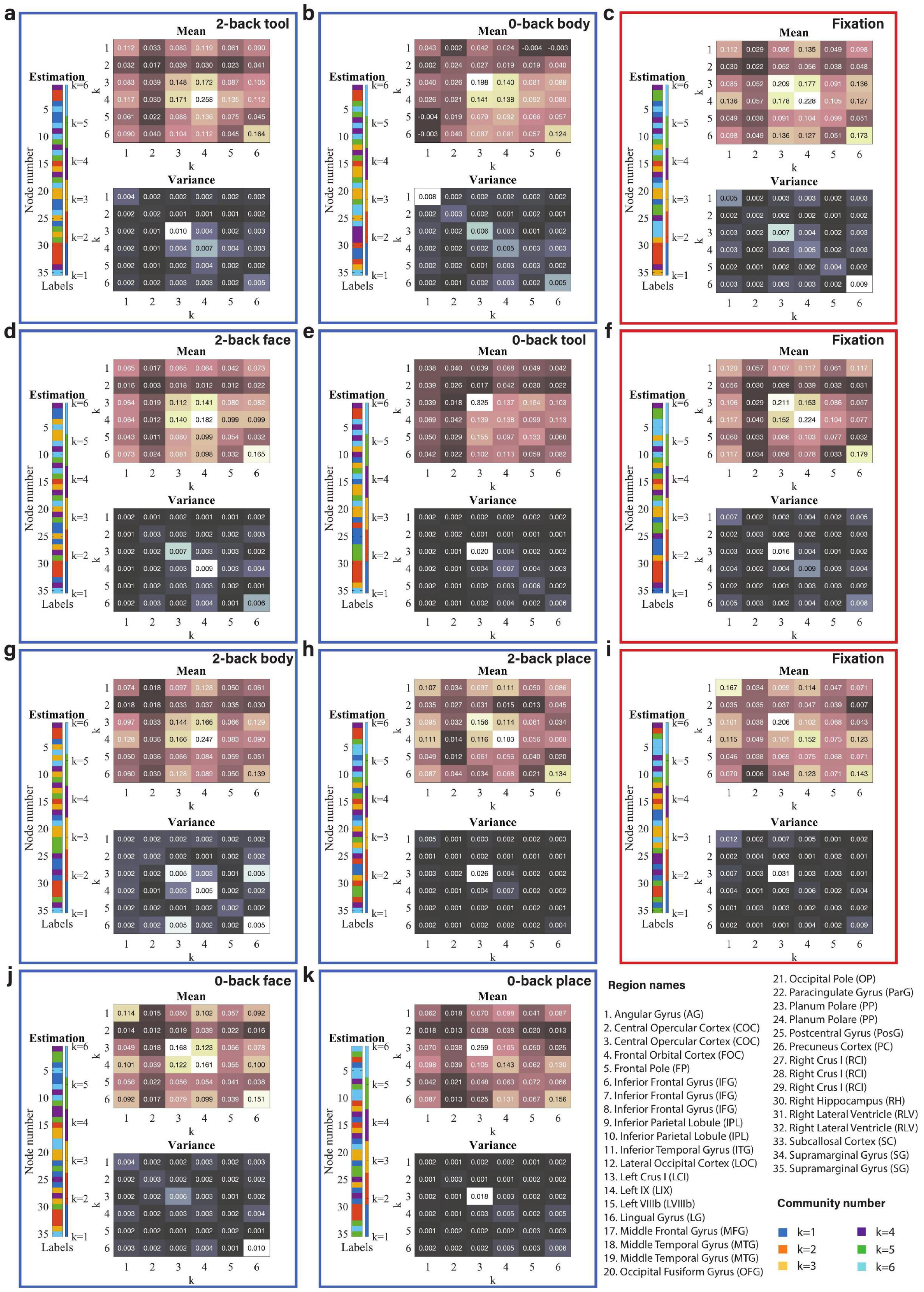

SI Figure 11. Estimated mean and variance matrices of the blocks for brain states (session 1, LR). The figures with blue frames represent brain states corresponding to working memory tasks (2-back tool at $t = 41$; 0-back body at $t = 76$; 2-back face at $t = 140$; 0-back tool at $t = 175$; 2-back body at $t = 239$; 2-back place at $t = 278$; 0-back face at $t = 334$; and 0-back place at $t = 375$ in **a-k**) and those with red frames represent brain states corresponding to fixation (fixation at $t = 107, 206$, and 306 in **c**, **f**, and **i**). The different colors of the labels represent community memberships. The density and variation of connectivity within and between communities are shown in the estimated block mean matrix and block variance matrix.

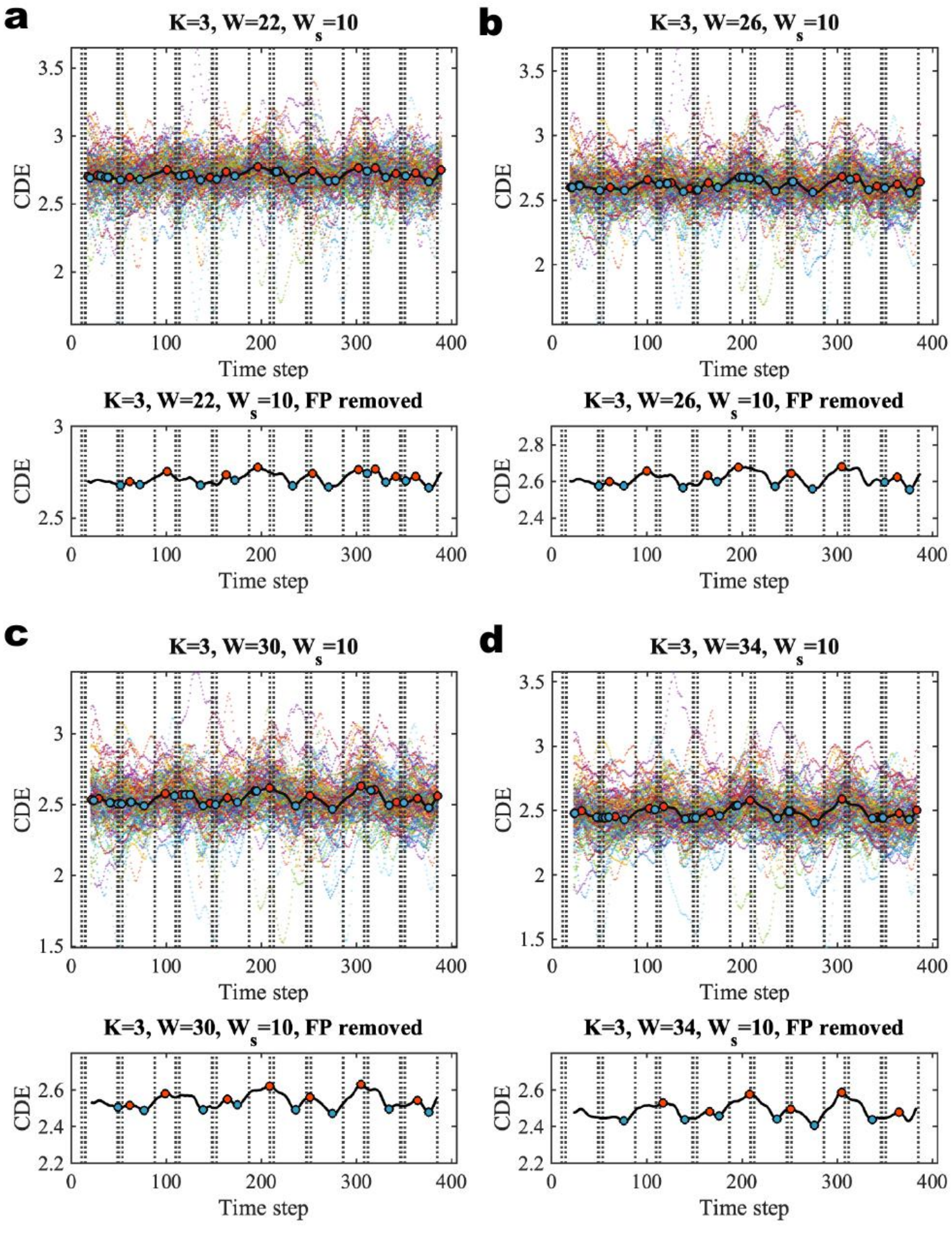

SI Figure 12. Results of Bayesian change-point detection for working memory tfMRI data (session 2, RL). The upper panels show the cumulative discrepancy energy (CDE) with different sliding window sizes (**a** $W = 22$, **b** $W = 26$, **c** $W = 30$, and **d** $W = 34$ under the model $K = 3$). W_s is the sliding window used for converting from PPDI to CDE. The vertical dashed lines are the times of onset of the stimuli, which are provided in the EV.txt files in the released data. The colourful scatterplots in the figures represent the CDEs of individual subjects and the black curve is the group-level CDE (averaged CDE over 100 subjects). The red dots are the local maxima, which are taken to be the locations of change-points, and the blue dots are the local minima, which are used for local inference of the discrete brain states. The bottom panels show the estimated group-averaged CDE where false positives (FP) are removed using time distance threshold $\tau = 9$.

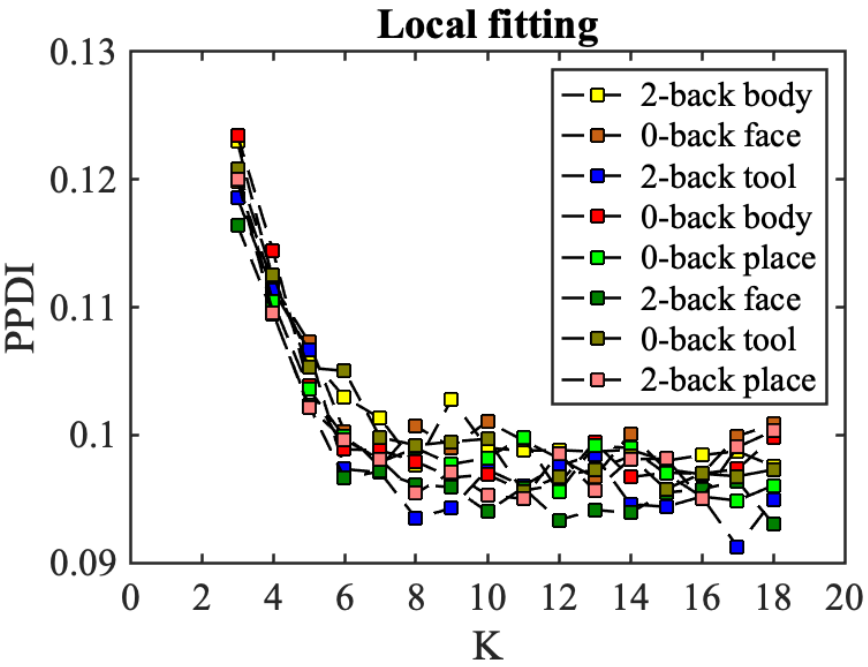

SI Figure 13. **Local fitting (session 2, RL) between averaged adjacency matrix and models from $K = 3$ to** $K = 18$. Different colours represent the PPDI values of different brain states.

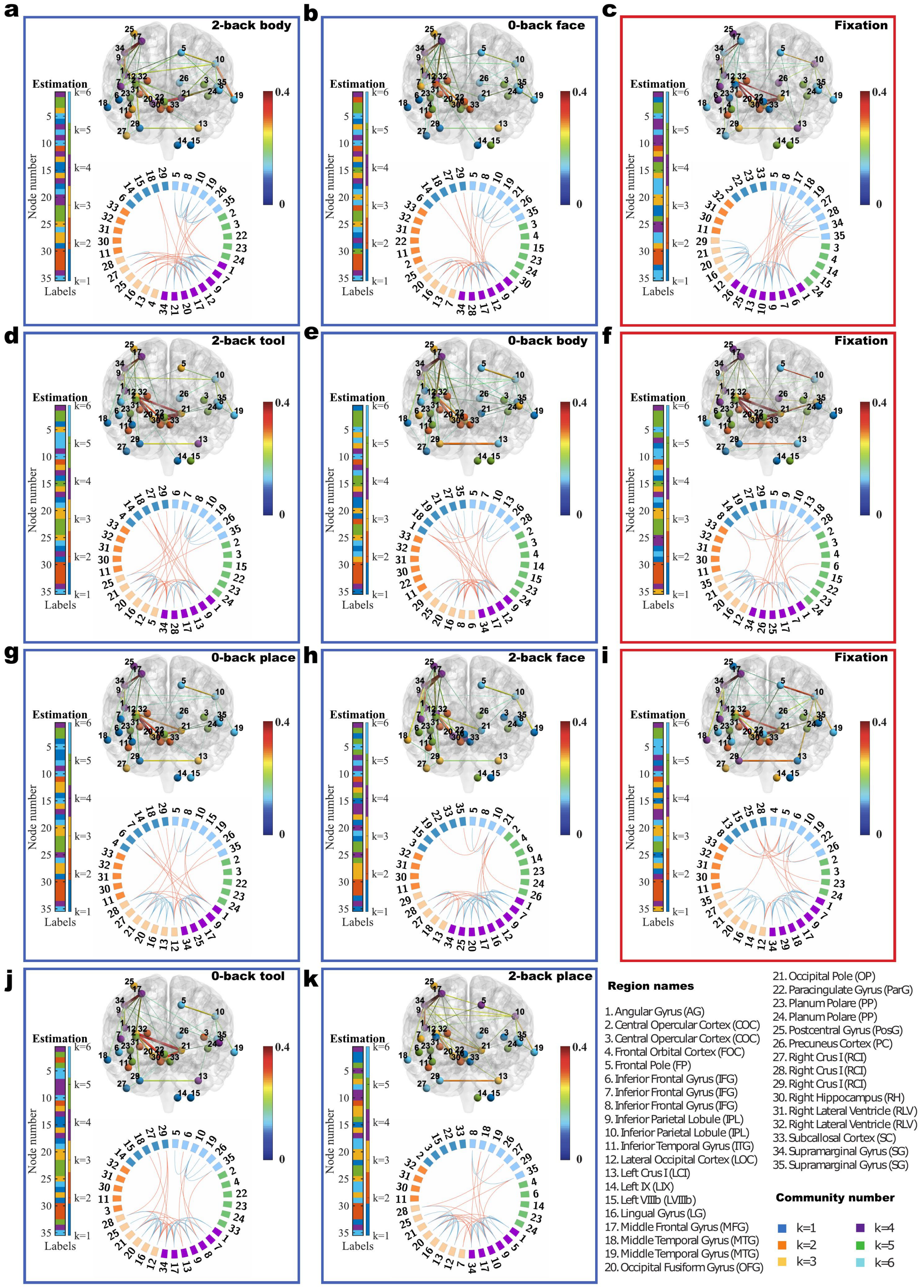

SI Figure 14. Community structure of the discrete brain states with sparsity level of 10% (session 2, RL). The figures with blue frames represent brain states corresponding to working memory tasks (2-back body at $t = 49$; 0-back face at $t = 77$; 2-back tool at $t = 139$; 0-back body at $t = 175$; 0-back place at $t = 236$; 2-back face at $t = 275$; 0-back tool at $t = 334$; and 2-back place at $t = 376$ in **a**-**k**) and those with red frames represent brain states corresponding to fixation (fixation at $t = 99$, 209, and 306 in **c**, **f**, and **i**).

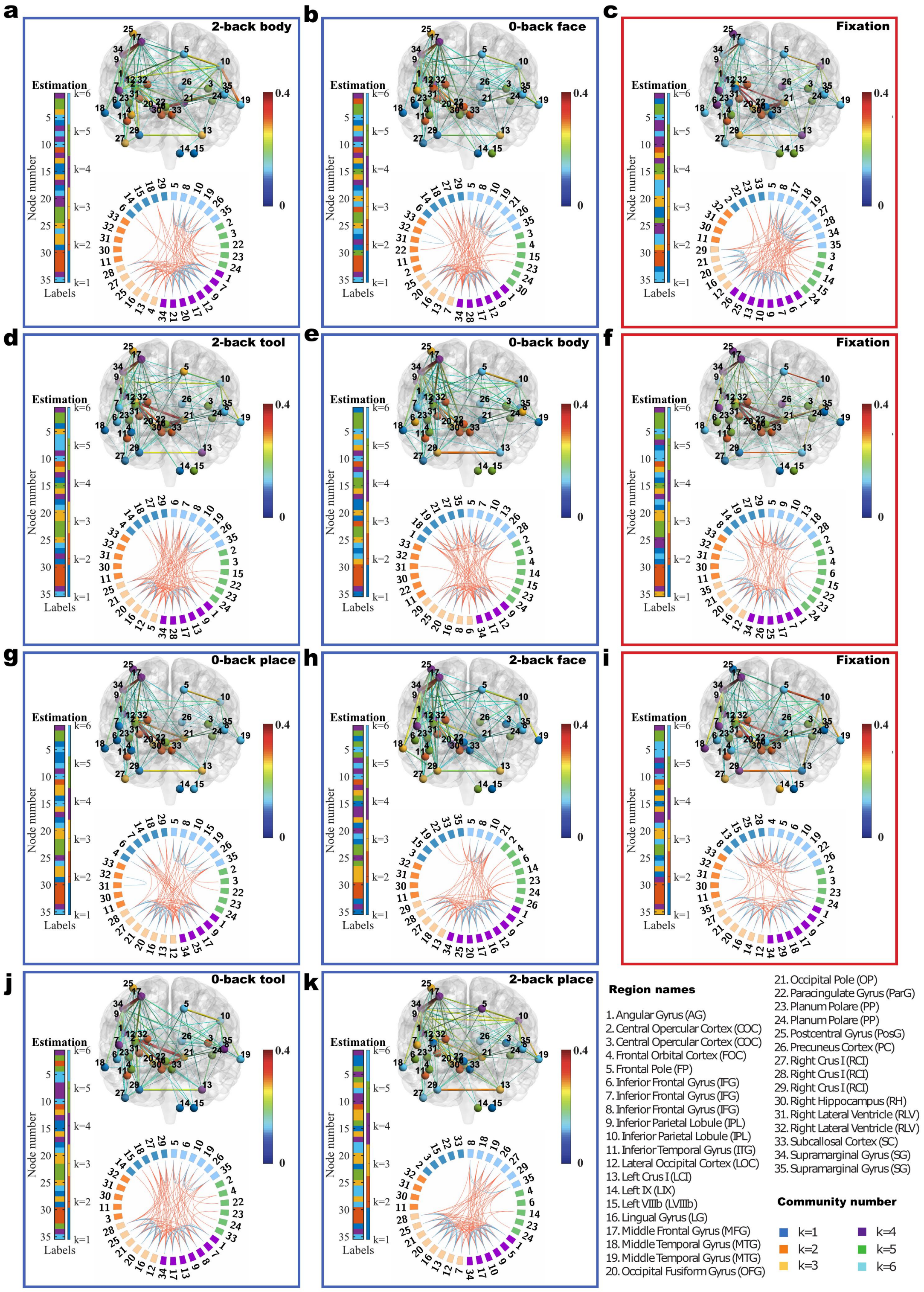

SI Figure 15. **Community structure of the discrete brain states with sparsity level of 20% (session 2, RL).** This figure is in the same format as the **SI Figure** 14 above only that it is for sparsity level of 20%.

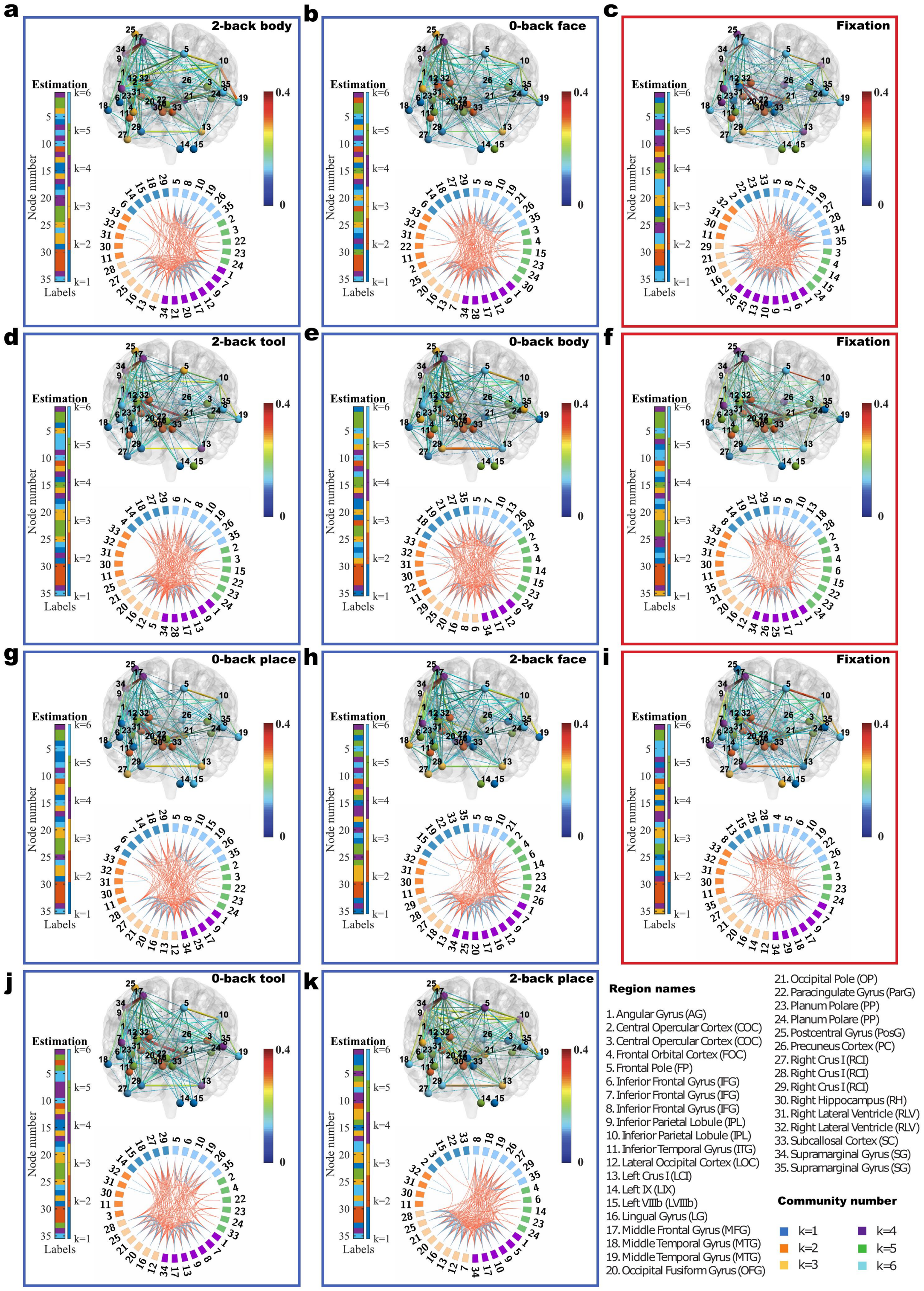

SI Figure 16. **Community structure of the discrete brain states with sparsity level of 30% (session 2, RL).** This figure is in the same format as the **SI Figure** 14 above only that it is for sparsity level of 30%.

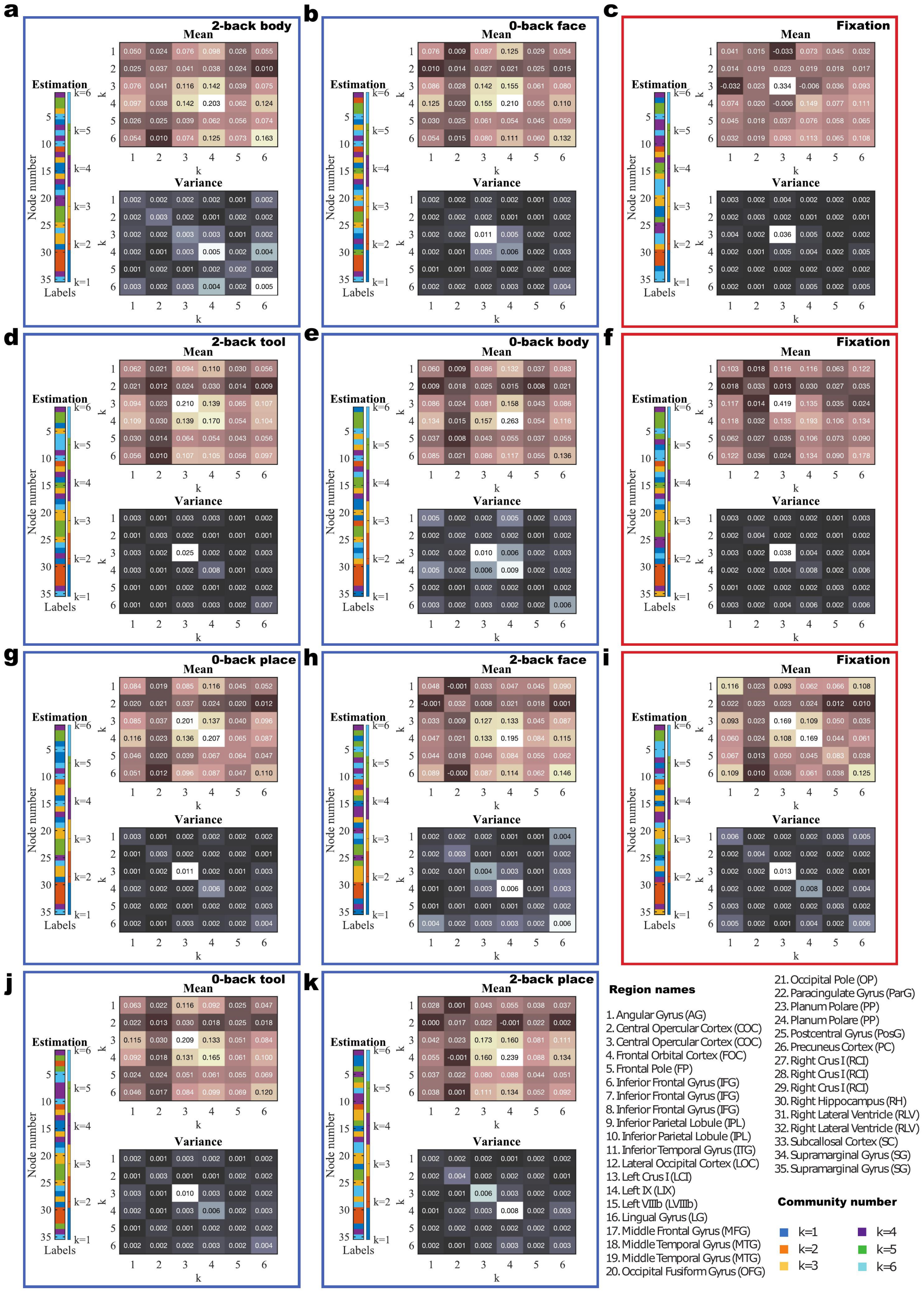

SI Figure 17. **Estimated mean and variance matrices of the blocks for brain states (session 2, RL).** This figure is in the same format as the **SI Figure** 11. The figures with blue frames represent brain states corresponding to working memory tasks (2-back body at $t = 49$; 0-back face at $t = 77$; 2-back tool at $t = 139$; 0-back body at $t = 175$; 0-back place at $t = 236$; 2-back face at $t = 275$; 0-back tool at $t = 334$; and 2-back place at $t = 376$ in **a**-**k**) and those with red frames represent brain states corresponding to fixation (fixation at $t = 99$, 209, and 306 in **c**, **f**, and **i**).

References

Carpaneto, G. and Toth, P. (1980). Algorithm 548: Solution of the assignment problem [H]. *ACM Transactions on Mathematical Software (TOMS)*, 6(1):104–111.

Eklund, A., Nichols, T. E., and Knutsson, H. (2016). Cluster failure: Why fMRI inferences for spatial extent have inflated false-positive rates. *PNAS*, 113(28):7900–7905.

Hastings, W. (1970). Monte Carlo sampling methods using Markov chains and their applications. *Biometrika*, 57(1):97–109.

MacDaid, A. F., Murphy, T. B., Friel, N., and Hurley, N. J. (2012). Improved Bayesian inference for the stochastic block model with application to large networks. *Computational Statistics and Data Analysis*, 60:12–31.

Nobile, A. and Fearnside, A. T. (2007). Bayesian finite mixtures with an unknown number of components: The allocation sampler. *Statistics and Computing*, 17:147–162.

Stephens, M. (2000). Dealing with label switching in mixture models. *Journal of the Royal Statistical Society. Series B (Statistical Methodology)*, 62(4):795–809.

Woolrich, M. W., Behrens, T. E., Beckmann, C. F., Jenkinson, M., and Smith, S. M. (2004). Multilevel linear modelling for FMRI group analysis using Bayesian inference. *NeuroImage*, 21(4):1732–1747.

Woolrich, M. W., Ripley, B. D., Brady, M., and Smith, S. M. (2001). Temporal autocorrelation in univariate linear modeling of FMRI data. *NeuroImage*, 14(6):1370–1386.

Wyse, J. and Friel, N. (2012). Block clustering with collapsed latent block models. *Statistics and Computing*, 22:415–428.
